# Supplementary material for: Novel Genes Required for the Fitness of Streptococcus pyogenes in Human Saliva
Source: mSphere. 2017 Nov 1;2(6):e00460-17. doi: 10.1128/mSphereDirect.00460-17 (PMC5663985; doi:10.1128/mSphereDirect.00460-17)
Supplement: TABLE S1 [file sph006172393st2.pdf]

TABLE S1 Essential genes identified in the genome of serotype M1 GAS strain MGAS2221 under the conditions tested.

No.	locus_tag	gene_name	start	end	strand	read_count	ins_index	gene_length	ins_count	fcn
1	M5005_Spy0001	dnaA	232	1587	1	66	0.002949853	1356	4	chromosomal replication initiator protein
2	M5005_Spy0002	dnaN	1742	2878	1	3	0.001759015	1137	2	DNA polymerase III beta chain
3	M5005_Spy0005	pth	4665	5234	1	0	0	570	0	peptidyl-tRNA hydrolase
4	M5005_Spy0008	divIC	9161	9532	1	77	0.010752688	372	4	cell division protein
5	M5005_Spy0011	tilS	10950	12236	1	1	0.000777001	1287	1	tRNA(Ile)-lysidine synthetase
6	M5005_Spy0012	M5005_Spy0012	12241	12783	1	1	0.001841621	543	1	hypoxanthine-guanine phosphoribosyltransferase
7	M5005_Spy0013	ftsH	12805	14784	1	1523	0.005050505	1980	10	cell division protein
8	M5005_Spy0017	sibA	31164	32360	1	49	0.000835422	1197	1	secreted protein
9	M5005_Spy0018	prsA2	32613	33575	1	186	0.007268951	963	7	ribose-phosphate pyrophosphokinase
10	M5005_Spy0020	plsX	34619	35626	1	3	0.000992063	1008	1	fatty acid/phospholipid synthesis protein
11	M5005_Spy0021	acpP2	35619	35861	1	0	0	243	0	acyl carrier protein
12	M5005_Spy0035	ruvB	54461	55459	1	21	0.009009009	999	9	holliday junction DNA helicase
13	M5005_Spy0037	M5005_Spy0037	56057	56458	1	0	0	402	0	putative membrane associated protein
14	M5005_Spy0038	M5005_Spy0038	56455	58230	1	18	0.001126126	1776	2	acyltransferase family
15	M5005_Spy0043	rpsJ	64330	64638	1	0	0	309	0	SSU ribosomal protein S10P
16	M5005_Spy0044	rplC	64854	65480	1	0	0	627	0	LSU ribosomal protein L3P
17	M5005_Spy0045	rplD	65504	66127	1	1	0.001602564	624	1	LSU ribosomal protein L1E
18	M5005_Spy0046	rplW	66127	66423	1	0	0	297	0	LSU ribosomal protein L23P
19	M5005_Spy0047	rplB	66441	67274	1	0	0	834	0	LSU ribosomal protein L2P
20	M5005_Spy0048	rpsS	67413	67691	1	0	0	279	0	SSU ribosomal protein S19P
21	M5005_Spy0049	rplV	67707	68051	1	0	0	345	0	LSU ribosomal protein L22P
22	M5005_Spy0050	rpsC	68064	68717	1	1	0.001529052	654	1	SSU ribosomal protein S3P
23	M5005_Spy0051	rplP	68721	69134	1	1	0.002415459	414	1	LSU ribosomal protein L16P
24	M5005_Spy0052	rpmC	69144	69350	1	0	0	207	0	LSU ribosomal protein L29P
25	M5005_Spy0053	rpsQ	69376	69636	1	1	0.003831418	261	1	SSU ribosomal protein S17P
26	M5005_Spy0054	rplN	69661	70029	1	3	0.005420054	369	2	LSU ribosomal protein L14P
27	M5005_Spy0055	rplX	70108	70413	1	0	0	306	0	LSU ribosomal protein L24P
28	M5005_Spy0056	rplE	70437	70979	1	1	0.001841621	543	1	LSU ribosomal protein L5P
29	M5005_Spy0057	rpsN	70995	71180	1	0	0	186	0	SSU ribosomal protein S14P
30	M5005_Spy0058	rpsH	71331	71729	1	0	0	399	0	SSU ribosomal protein S8P
31	M5005_Spy0059	rplF	71932	72468	1	0	0	537	0	LSU ribosomal protein L6P
32	M5005_Spy0060	rplR	72573	72929	1	0	0	357	0	LSU ribosomal protein L18P
33	M5005_Spy0061	rpsE	72948	73442	1	1	0.002020202	495	1	SSU ribosomal protein S5P
34	M5005_Spy0062	rpmD	73457	73639	1	1	0.005464481	183	1	LSU ribosomal protein L30P
35	M5005_Spy0063	rplO	73853	74293	1	0	0	441	0	LSU ribosomal protein L15P
36	M5005_Spy0064	secY	74310	75614	1	0	0	1305	0	protein translocase subunit
37	M5005_Spy0065	adk	75764	76402	1	276	0.00312989	639	2	adenylate kinase
38	M5005_Spy0066	infA	76520	76738	1	0	0	219	0	bacterial protein translation initiation factor 1
39	M5005_Spy0067	rpmJ	76764	76880	1	0	0	117	0	LSU ribosomal protein L36P
40	M5005_Spy0068	rpsM	76898	77263	1	0	0	366	0	SSU ribosomal protein S13P
41	M5005_Spy0069	rpsK	77281	77664	1	1	0.002604167	384	1	SSU ribosomal protein S11P
42	M5005_Spy0070	rpoA	77710	78648	1	1	0.001064963	939	1	DNA-directed RNA polymerase alpha chain
43	M5005_Spy0071	rplQ	78663	79049	1	0	0	387	0	LSU ribosomal protein L17P
44	M5005_Spy0081	tyrS	90247	91503	-1	2	0.00159109	1257	2	tyrosyl-tRNA synthetase
45	M5005_Spy0083	rpoB	94171	97737	1	1	0.000280348	3567	1	DNA-directed RNA polymerase beta chain
46	M5005_Spy0084	rpoC	97828	101469	1	1369	0.001922021	3642	7	DNA-directed RNA polymerase beta' chain
47	M5005_Spy0094	ackA	106806	108002	1	24	0.000835422	1197	1	acetate kinase
48	M5005_Spy0138	nusG	149282	149821	1	70	0.005555556	540	3	transcription antitermination protein
49	M5005_Spy0140	M5005_Spy0140	151552	152037	1	113	0.00617284	486	3	hypothetical protein
50	M5005_Spy0147	leuS	157881	160268	1	30	0.001675042	2388	4	leucyl-tRNA synthetase
51	M5005_Spy0159	polA	172165	174807	1	174	0.003783579	2643	10	DNA polymerase I
52	M5005_Spy0179	M5005_Spy0179	188408	188923	1	20	0.007751938	516	4	tRNA-specific adenosine deaminase
53	M5005_Spy0185	pgi	192329	193678	1	129	0.003703704	1350	5	glucose-6-phosphate isomerase
54	M5005_Spy0192	hasC2	198307	199206	-1	28	0.001111111	900	1	UTP--glucose-1-phosphate uridylyltransferase
55	M5005_Spy0194	gpsA	199239	200255	-1	0	0	1017	0	glycerol-3-phosphate dehydrogenase [NAD(P)+]
56	M5005_Spy0203	gltX	209080	210525	1	141	0.002074689	1446	3	glutamyl-tRNA synthetase
57	M5005_Spy0207	fasX	214830	215189	1	311	0.008333333	360	3	ribonuclease P protein component
58	M5005_Spy0211	rpmH	217223	217357	1	0	0	135	0	LSU ribosomal protein L34P
59	M5005_Spy0223	M5005_Spy0223	228230	229102	1	54	0.004581901	873	4	GTPase
60	M5005_Spy0224	rpe	229112	229774	1	755	0.013574661	663	9	ribulose-phosphate 3-epimerase
61	M5005_Spy0230	rpsL	236526	236939	1	0	0	414	0	SSU ribosomal protein S12P
62	M5005_Spy0231	rpsG	236960	237430	1	74	0.004246285	471	2	SSU ribosomal protein S7P
63	M5005_Spy0232	fus	237797	239875	1	221	0.001924002	2079	4	protein translation elongation factor G
64	M5005_Spy0233	plr	240223	241233	1	312	0.002967359	1011	3	glyceraldehyde 3-phosphate dehydrogenase
65	M5005_Spy0235	M5005_Spy0235	241717	242457	-1	1	0.001349528	741	1	amino acid transport ATP-binding protein
66	M5005_Spy0236	M5005_Spy0236	242450	244018	-1	0	0	1569	0	ABC transporter amino acid-binding protein/amino acid ABC transporter permease protein
67	M5005_Spy0239	mecA	247165	247926	1	115	0.007874016	762	6	negative regulator of genetic competence
68	M5005_Spy0240	M5005_Spy0240	248077	249102	1	3288	0.010721248	1026	11	undecaprenyl-phosphate alpha-N-acetylglucosaminephosphotransferase
69	M5005_Spy0260	M5005_Spy0260	272715	273299	1	393	0.003418803	585	2	putative lipase
70	M5005_Spy0261	M5005_Spy0261	273299	274417	1	1	0.000893655	1119	1	GTP-binding protein
71	M5005_Spy0263	nadD	274819	275451	1	0	0	633	0	nicotinate-nucleotide adenylyltransferase
72	M5005_Spy0264	M5005_Spy0264	275448	276041	1	38	0.005050505	594	3	hydrolase
73	M5005_Spy0270	M5005_Spy0270	279970	280812	1	341	0.008303677	843	7	ABC transporter substrate-binding protein
74	M5005_Spy0276	M5005_Spy0276	286920	287594	-1	145	0.002962963	675	2	potassium uptake protein
75	M5005_Spy0277	M5005_Spy0277	287604	288995	-1	0	0	1392	0	potassium uptake protein
76	M5005_Spy0281	M5005_Spy0281	291662	292195	1	11	0.007490637	534	4	putative cytosolic protein
77	M5005_Spy0282	covR	292462	293148	1	3	0.004366812	687	3	response regulator
78	M5005_Spy0283	covS	293291	294655	1	264	0.002197802	1365	3	transmembrane histidine kinase
79	M5005_Spy0284	M5005_Spy0284	294870	295364	1	453	0.014141414	495	7	putative regulatory protein
80	M5005_Spy0285	dnaB	295348	296523	1	1	0.00085034	1176	1	replicative DNA helicase
81	M5005_Spy0286	dnaI	296524	297426	1	1	0.00110742	903	1	primosomal protein
82	M5005_Spy0287	pgdA	297489	298799	1	1	0.000762777	1311	1	GTP-binding protein
83	M5005_Spy0289	M5005_Spy0289	302347	302949	1	0	0	603	0	putative cytosolic protein
84	M5005_Spy0290	murC	302989	304317	1	187	0.003009782	1329	4	UDP-N-acetylmuramate--alanine ligase
85	M5005_Spy0292	M5005_Spy0292	304963	306531	1	178	0.007648184	1569	12	aminodeoxychorismate lyase family
86	M5005_Spy0295	M5005_Spy0295	307709	308632	-1	332	0.014069264	924	13	60 kDa inner membrane protein YIDC
87	M5005_Spy0302	M5005_Spy0302	312725	312967	1	0	0	243	0	putative exported protein
88	M5005_Spy0303	glr	313145	313939	1	0	0	795	0	glutamate racemase
89	M5005_Spy0319	ppaC	325505	326440	1	85	0.00534188	936	5	manganese-dependent inorganic pyrophosphatase
90	M5005_Spy0325	murE	331240	332685	-1	5	0.002074689	1446	3	UDP-N-acetylmuramoyl-L-alanyl-D-glutamate-- lysine ligase
91	M5005_Spy0326	M5005_Spy0326	332773	334407	1	1623	0.001223242	1635	2	export protein for polysaccharides and teichoic acids
92	M5005_Spy0328	clpP	335428	336018	1	232	0.013536379	591	8	ATP-dependent Clp protease proteolytic subunit
93	M5005_Spy0330	tmk	337034	337669	1	19	0.003144654	636	2	thymidylate kinase
94	M5005_Spy0331	dnaX	337687	338562	1	215	0.006849315	876	6	DNA polymerase III
95	M5005_Spy0334	M5005_Spy0334	339225	339548	1	27	0.00617284	324	2	hypothetical protein
96	M5005_Spy0345	metS	351409	353406	1	28	0.003003003	1998	6	methionyl-tRNA synthetase
97	M5005_Spy0362	gcaD	367792	369174	1	315	0.002169197	1383	3	glucosamine-1-phosphate acetyltransferase/UDP-N-acetylglucosamine pyrophosphorylase
98	M5005_Spy0365	pfs	370030	370725	1	271	0.001436782	696	1	5'-methylthioadenosine nucleosidase/S-adenosylhomocysteine nucleosidase
99	M5005_Spy0368	mtsA	372123	373043	1	5	0.004343105	921	4	manganese-binding protein
100	M5005_Spy0369	mtsB	373107	373832	1	2	0.002754821	726	2	manganese transport system ATP-binding protein
101	M5005_Spy0370	mtsC	373833	374687	1	16	0.007017544	855	6	manganese transport system membrane protein
102	M5005_Spy0372	ftsK	375858	378263	1	444	0.00166251	2406	4	cell division protein
103	M5005_Spy0374	rplK	378930	379355	1	0	0	426	0	LSU ribosomal protein L11P
104	M5005_Spy0375	rplA	379461	380150	1	4	0.001449275	690	1	LSU ribosomal protein L1P
105	M5005_Spy0377	pyrH	381799	382527	1	42	0.005486968	729	4	uridylate kinase
106	M5005_Spy0380	rrf	382556	383113	1	11	0.001792115	558	1	ribosome recycling factor
107	M5005_Spy0388	M5005_Spy0388	390224	390772	1	0	0	549	0	putative metal-binding protein
108	M5005_Spy0389	dgk	390753	391160	1	0	0	408	0	diacylglycerol kinase
109	M5005_Spy0390	era	391280	392176	1	15	0.002229654	897	2	GTP-binding protein-like protein
110	M5005_Spy0409	M5005_Spy0409	401625	402305	1	54	0.010279001	681	7	dephospho-CoA kinase
111	M5005_Spy0413	secG	405501	405737	1	24	0.012658228	237	3	protein translocase subunit
112	M5005_Spy0424	ccpA	415424	416425	1	0	0	1002	0	catabolite control protein A
113	M5005_Spy0425	M5005_Spy0425	416556	417554	1	0	0	999	0	glycosyltransferase
114	M5005_Spy0426	M5005_Spy0426	417556	418890	1	1	0.000749064	1335	1	1,2-diacylglycerol 3-glucosyltransferase
115	M5005_Spy0427	thrS	419312	421255	1	550	0.007201646	1944	14	threonyl-tRNA synthetase
116	M5005_Spy0435	vicR	428344	429054	1	1	0.00140647	711	1	two-component response regulator
117	M5005_Spy0438	acpA	431656	432348	1	47	0.007215007	693	5	ribonuclease III
118	M5005_Spy0456	M5005_Spy0456	450349	450651	1	153	0.01320132	303	4	plasmid stabilization system antitoxin protein
119	M5005_Spy0472	ftsY	460643	462193	1	3595	0.007092199	1551	11	cell division protein

120	M5005_Spy0484	ptsK	473608	474633	1	484	0.011695906	1026	12	hpr(ser) kinase
121	M5005_Spy0488	M5005_Spy0488	476418	476702	-1	0	0	285	0	hypothetical protein
122	M5005_Spy0495	lysS	479999	481492	-1	0	0	1494	0	lysyl-tRNA synthetase
123	M5005_Spy0506	ftsW	492532	493806	1	203	0.007058824	1275	9	cell division protein
124	M5005_Spy0508	M5005_Spy0508	494160	495356	1	1	0.000835422	1197	1	protein translation elongation factor Tu
125	M5005_Spy0509	tpi	495597	496355	1	392	0.005270092	759	4	triosephosphate isomerase
126	M5005_Spy0510	M5005_Spy0510	496454	497689	-1	224	0.003236246	1236	4	factor essential for expression of methicillin resistance
127	M5005_Spy0511	murM	497676	498902	-1	2	0.001629992	1227	2	UDP-N-acetylmuramoylpentapeptide-lysine N(6)-alanyltransferase/UDP-N-acetylmuramoylpentapeptide- lysine N(6)-seryltransferase
128	M5005_Spy0512	M5005_Spy0512	498902	499711	-1	1	0.001234568	810	1	hydrolase
129	M5005_Spy0516	pacL	502167	504848	1	153	0.00186428	2682	5	calcium-transporting ATPase
130	M5005_Spy0530	prfB	518136	519149	1	2	0.001972387	1014	2	bacterial peptide chain release factor 2
131	M5005_Spy0531	ftsE	519168	519860	1	0	0	693	0	cell division ATP-binding protein
132	M5005_Spy0532	ftsX	519853	520782	1	76	0.003225806	930	3	cell division protein
133	M5005_Spy0538	asnS	526880	528226	1	7	0.00074239	1347	1	asparaginyl-tRNA synthetase
134	M5005_Spy0546	rpmE	536826	537086	-1	6	0.003831418	261	1	LSU ribosomal protein L31P
135	M5005_Spy0547	M5005_Spy0547	537201	538142	-1	17	0.007430998	942	7	phosphoesterase
136	M5005_Spy0548	M5005_Spy0548	538536	538985	1	178	0.008888889	450	4	flavodoxin
137	M5005_Spy0551	rplS	540815	541162	1	0	0	348	0	LSU ribosomal protein L19P
138	M5005_Spy0552	M5005_Spy0552	542165	542734	1	3	0.005263158	570	3	DNA gyrase related protein
139	M5005_Spy0553	gyrB	542735	544687	1	0	0	1953	0	DNA gyrase subunit B
140	M5005_Spy0554	M5005_Spy0554	545055	546779	1	100	0.002898551	1725	5	septation ring formation regulator
141	M5005_Spy0556	eno	547596	548903	1	297	0.003058104	1308	4	enolase
142	M5005_Spy0573	lig	572301	574259	1	318	0.001020929	1959	2	NAD-dependent DNA ligase
143	M5005_Spy0574	M5005_Spy0574	574273	575295	1	231	0.010752688	1023	11	diacylglycerol kinase family
144	M5005_Spy0575	atpE	575688	575885	1	0	0	198	0	ATP synthase C chain
145	M5005_Spy0576	atpB	575920	576636	1	0	0	717	0	ATP synthase A chain
146	M5005_Spy0577	atpF	576654	577148	1	0	0	495	0	ATP synthase B chain
147	M5005_Spy0578	atpH	577148	577684	1	0	0	537	0	ATP synthase delta chain
148	M5005_Spy0579	atpA	577700	579208	1	0	0	1509	0	ATP synthase alpha chain
149	M5005_Spy0580	atpG	579224	580099	1	4	0.002283105	876	2	ATP synthase gamma chain
150	M5005_Spy0581	atpD	580261	581667	1	3	0.001421464	1407	2	ATPase
151	M5005_Spy0582	atpC	581680	582096	1	20	0.011990408	417	5	ATP synthase epsilon chain
152	M5005_Spy0587	pheS	585005	586048	1	0	0	1044	0	phenylalanyl-tRNA synthetase alpha chain
153	M5005_Spy0588	pheT	586243	588663	1	3	0.000826105	2421	2	phenylalanyl-tRNA synthetase beta chain
154	M5005_Spy0595	rexA	595702	599370	1	432	0.013355138	3669	49	ATP-dependent nuclease subunit A
155	M5005_Spy0597	M5005_Spy0597	600433	600639	1	0	0	207	0	SSU ribosomal protein S21P
156	M5005_Spy0599	dnaG	601260	603074	1	0	0	1815	0	DNA primase
157	M5005_Spy0600	rpoD	603083	604192	1	125	0.000900901	1110	1	RNA polymerase sigma factor
158	M5005_Spy0602	rmlD	604904	605758	1	0	0	855	0	dTDP-4-dehydrorhamnose reductase
159	M5005_Spy0603	rgpAc	605877	607031	1	1428	0.001731602	1155	2	alpha-(1,2)-rhamnosyltransferase
160	M5005_Spy0604	rgpBc	607021	607953	1	1428	0.002143623	933	2	alpha-L-Rha alpha-1,3-L-rhamnosyltransferase
161	M5005_Spy0605	rgpCc	607956	608759	1	0	0	804	0	polysaccharide export ABC transporter permease protein
162	M5005_Spy0606	rgpDc	608759	609964	1	202	0.002487562	1206	3	polysaccharide export ATP-binding protein
163	M5005_Spy0607	rgpEc	609989	610996	1	1	0.000992063	1008	1	glycosyltransferase
164	M5005_Spy0608	rgpFc	610993	612738	1	342	0.005154639	1746	9	alpha-1,3-L-rhamnosyltransferase
165	M5005_Spy0613	M5005_Spy0613	617686	619182	1	676	0.005344021	1497	8	putative membrane spanning protein
166	M5005_Spy0615	ebsA	620540	621028	1	5	0.008179959	489	4	pore forming protein
167	M5005_Spy0616	M5005_Spy0616	621015	621212	-1	7	0.005050505	198	1	ferredoxin
168	M5005_Spy0617	M5005_Spy0617	621261	621737	1	5	0.002096436	477	1	putative membrane associated protein
169	M5005_Spy0619	infC	622594	623124	1	0	0	531	0	bacterial protein translation initiation factor 3
170	M5005_Spy0620	rpl36	623166	623363	1	0	0	198	0	LSU ribosomal protein L35P
171	M5005_Spy0621	rplT	623422	623781	1	0	0	360	0	LSU ribosomal protein L20P
172	M5005_Spy0622	M5005_Spy0622	624072	626243	-1	349	0.001841621	2172	4	phosphoglycerol transferase
173	M5005_Spy0633	rplU	637444	637758	1	0	0	315	0	LSU ribosomal protein L21P
174	M5005_Spy0634	M5005_Spy0634	637770	638096	1	0	0	327	0	putative ribosome-associated protein
175	M5005_Spy0635	rpmA	638124	638417	1	1	0.003401361	294	1	LSU ribosomal protein L27P
176	M5005_Spy0638	M5005_Spy0638	640124	641014	1	38	0.01010101	891	9	ribosomal large subunit pseudouridine synthase D
177	M5005_Spy0648	rpsP	654264	654536	1	20	0.010989011	273	3	SSU ribosomal protein S16P
178	M5005_Spy0655	rimM	661070	661588	1	0	0	519	0	16S rRNA processing protein
179	M5005_Spy0656	trmD	661578	662309	1	596	0.00273224	732	2	tRNA (guanine-N(1)-)-methyltransferase
180	M5005_Spy0657	trxB	662309	663301	1	123	0.013091641	993	13	thioredoxin reductase
181	M5005_Spy0671	M5005_Spy0671	673722	674102	1	0	0	381	0	transposase
182	M5005_Spy0672	M5005_Spy0672	674102	674950	1	99	0.011778563	849	10	degV family protein
183	M5005_Spy0673	papS	675075	676283	1	601	0.00165426	1209	2	poly(A) polymerase/tRNA nucleotidyltransferase
184	M5005_Spy0682	mvaK1	684614	685507	1	0	0	894	0	mevalonate kinase
185	M5005_Spy0683	mvaD	685489	686433	1	0	0	945	0	diphosphomevalonate decarboxylase
186	M5005_Spy0684	mvaK2	686426	687433	1	1	0.000992063	1008	1	phosphomevalonate kinase
187	M5005_Spy0685	M5005_Spy0685	687426	688415	1	5	0.001010101	990	1	isopentenyl-diphosphate delta-isomerase
188	M5005_Spy0686	M5005_Spy0686	688645	689922	-1	44	0.001564945	1278	2	3-hydroxy-3-methylglutaryl-coenzyme A reductase
189	M5005_Spy0687	mvaS1	689909	691084	-1	1	0.00085034	1176	1	hydroxymethylglutaryl-CoA synthase
190	M5005_Spy0688	thyA	691293	692132	1	1	0.001190476	840	1	thymidylate synthase
191	M5005_Spy0689	dyr	692212	692709	1	5	0.004016064	498	2	dihydrofolate reductase
192	M5005_Spy0692	M5005_Spy0692	694268	694867	1	172	0.003333333	600	2	GTP-binding protein
193	M5005_Spy0695	rpiA	698294	698977	1	14	0.00877193	684	6	ribose 5-phosphate isomerase
194	M5005_Spy0696	deoB	699054	700265	1	20	0.00990099	1212	12	phosphopentomutase
195	M5005_Spy0698	punA	700708	701517	1	28	0.009876543	810	8	purine nucleoside phosphorylase
196	M5005_Spy0710	M5005_Spy0710	712627	713268	-1	0	0	642	0	putative membrane spanning protein
197	M5005_Spy0711	parE	713403	715352	1	9	0.001025641	1950	2	topoisomerase IV subunit B
198	M5005_Spy0712	parC	715443	717902	1	185	0.00203252	2460	5	topoisomerase IV subunit A
199	M5005_Spy0714	M5005_Spy0714	719111	719341	1	3	0.008658009	231	2	putative cytosolic protein
200	M5005_Spy0715	M5005_Spy0715	719732	720937	1	25	0.00331675	1206	4	SSU ribosomal protein S1P
201	M5005_Spy0725	elaC	728127	729056	1	952	0.004301075	930	4	metal-dependent hydrolase
202	M5005_Spy0729	dnaD	732777	733460	1	0	0	684	0	DNA replication protein
203	M5005_Spy0731	M5005_Spy0731	734185	734871	1	49	0.002911208	687	2	para-aminobenzoate synthase component I/anthranilate synthase component I
204	M5005_Spy0734	cpsFO	736853	737722	1	0	0	870	0	glucose-1-phosphate thymidylyltransferase
205	M5005_Spy0735	cpsFP	737722	738315	1	1	0.001683502	594	1	dTDP-4-dehydrorhamnose 3,5-epimerase
206	M5005_Spy0736	cpsFQ	738559	739599	1	537	0.006724304	1041	7	dTDP-glucose 4,6-dehydratase
207	M5005_Spy0747	M5005_Spy0747	748009	749670	1	26	0.001203369	1662	2	Zn-dependent hydrolase
208	M5005_Spy0753	acoC	755392	756801	1	33	0.009929078	1410	14	dihydrolipoamide acetyltransferase component of pyruvate dehydrogenase complex
209	M5005_Spy0759	M5005_Spy0759	763313	764104	-1	195	0.008838384	792	7	putative membrane associated protein
210	M5005_Spy0760	M5005_Spy0760	764104	765447	-1	1	0.000744048	1344	1	UDP-N-acetylmuramoylalanyl-D-glutamate--2, 6-diaminopimelate ligase
211	M5005_Spy0761	M5005_Spy0761	765554	766405	1	1	0.001173709	852	1	putative membrane spanning protein
212	M5005_Spy0763	femD	767412	768767	1	1416	0.005162242	1356	7	phosphoglucosamine mutase
213	M5005_Spy0792	M5005_Spy0792	797080	797682	1	24	0.013266998	603	8	NAD(P)H-dependent quinone reductase
214	M5005_Spy0794	thdF	799284	800660	-1	6	0.002178649	1377	3	tRNA (5-carboxymethylaminomethyl-2-thiouridylate) synthase
215	M5005_Spy0795	rplJ	800897	801493	1	1	0.001675042	597	1	LSU ribosomal protein L10P
216	M5005_Spy0796	rplL	801558	801923	1	0	0	366	0	LSU ribosomal protein L12P
217	M5005_Spy0820	folC1	815143	816420	1	18	0.00312989	1278	4	folylpolyglutamate synthase/dihydrofolate synthase
218	M5005_Spy0821	folE	816467	817033	1	0	0	567	0	GTP cyclohydrolase I
219	M5005_Spy0822	folP	817042	817842	1	9	0.001248439	801	1	dihydropteroate synthase
220	M5005_Spy0823	folQ	817849	818208	1	0	0	360	0	dihydroneopterin aldolase
221	M5005_Spy0825	murB	818855	819742	1	110	0.003378378	888	3	UDP-N-acetylenolpyruvoylglucosamine reductase
222	M5005_Spy0842	M5005_Spy0842	835819	836463	-1	2	0.003100775	645	2	redox-sensitive transcriptional regulator Rex
223	M5005_Spy0844	nifS2	837052	838179	-1	0	0	1128	0	cysteine desulfhydrase
224	M5005_Spy0845	M5005_Spy0845	838176	839156	-1	0	0	981	0	ribose-phosphate pyrophosphokinase
225	M5005_Spy0848	M5005_Spy0848	840608	841444	1	650	0.004778973	837	4	ATP-NAD kinase
226	M5005_Spy0863	prfA	853206	854285	1	14	0.000925926	1080	1	bacterial peptide chain release factor 1
227	M5005_Spy0865	M5005_Spy0865	855108	855698	1	2	0.003384095	591	2	SUA5 protein
228	M5005_Spy0874	gyrA	865400	867886	1	669	0.004423	2487	11	DNA gyrase subunit A
229	M5005_Spy0875	M5005_Spy0875	867906	868655	1	196	0.008	750	6	sortase
230	M5005_Spy0880	M5005_Spy0880	871211	871861	-1	69	0.013824885	651	9	conserved membrane protein
231	M5005_Spy0882	M5005_Spy0882	872517	873365	1	101	0.003533569	849	3	GTP-binding protein
232	M5005_Spy0891	satD	879910	880575	1	900	0.012012012	666	8	SatD
233	M5005_Spy0913	xerD	899160	900230	-1	6	0.003734827	1071	4	recombinase
234	M5005_Spy0915	ffh	901514	903076	-1	266	0.01087652	1563	17	signal recognition particle
235	M5005_Spy0916	ylxM	903091	903432	-1	89	0.00877193	342	3	signal recognition particle associated protein
236	M5005_Spy0919	guaA	905902	907464	1	41	0.006397953	1563	10	GMP synthase (glutamine-hydrolyzing)
237	M5005_Spy0927	fhs1	916269	917939	-1	24	0.001795332	1671	3	formate--tetrahydrofolate ligase
238	M5005_Spy0935	dpfB	924409	925101	1	171	0.008658009	693	6	phosphopantothenate--cysteine ligase
239	M5005_Spy0936	dfp	925094	925639	1	177	0.014652015	546	8	phosphopantothenoylcysteine decarboxylase
240	M5005_Spy0937	M5005_Spy0937	925697	926266	1	491	0.014035088	570	8	transporter
241	M5005_Spy0938	pgmA	926442	928160	1	1938	0.005817336	1719	10	phosphoglucomutase/phosphomannomutase

242	M5005_Spy0945	coaA	935108	936028	1	15	0.002171553	921	2	pantothenate kinase
243	M5005_Spy0950	phoU	941520	942173	-1	15	0.009174312	654	6	phosphate transport system protein
244	M5005_Spy0959	M5005_Spy0959	949030	949434	-1	0	0	405	0	arsenate reductase family protein
245	M5005_Spy0960	mreA	949477	950409	-1	0	0	933	0	riboflavin kinase/FMN adenylyltransferase
246	M5005_Spy0977	pcrA	962240	964558	-1	38	0.005174644	2319	12	DNA helicase II
247	M5005_Spy0978	M5005_Spy0978	965087	966409	1	164	0.012093726	1323	16	Na(+)-linked D-alanine glycine permease
248	M5005_Spy0979	M5005_Spy0979	964926	965096	-1	0	0	171	0	hypothetical protein
249	M5005_Spy0986	glmS	972045	973859	-1	0	0	1815	0	glucosamine--fructose-6-phosphate aminotransferase (isomerizing)
250	M5005_Spy0988	pyk	974810	976312	-1	0	0	1503	0	pyruvate kinase
251	M5005_Spy0989	pfk	976375	977388	-1	3	0.001972387	1014	2	non-allosteric 6-phosphofructokinase
252	M5005_Spy0990	dnaE	977468	980578	-1	3	0.00064288	3111	2	DNA polymerase III alpha subunit
253	M5005_Spy0991	M5005_Spy0991	980763	981134	1	53	0.008064516	372	3	transcriptional regulator
254	M5005_Spy0994	M5005_Spy0994	982754	983368	-1	0	0	615	0	putative membrane-associated alkaline phosphatase
255	M5005_Spy1070	dltD	1042897	1044147	-1	116	0.003996803	1251	5	protein precursor
256	M5005_Spy1071	M5005_Spy1071	1044140	1044379	-1	0	0	240	0	D-alanyl carrier protein
257	M5005_Spy1072	M5005_Spy1072	1044397	1045653	-1	6	0.00159109	1257	2	protein DltB
258	M5005_Spy1073	dltA	1045650	1047188	-1	2	0.000649773	1539	1	D-alanine-activating enzyme
259	M5005_Spy1074	M5005_Spy1074	1047200	1047343	-1	0	0	144	0	hypothetical protein
260	M5005_Spy1076	glnH	1049791	1051965	1	1	0.00045977	2175	1	transporter
261	M5005_Spy1077	glnQ2	1051965	1052705	1	0	0	741	0	glutamine transport ATP-binding protein
262	M5005_Spy1088	obg	1061432	1062745	-1	2	0.00152207	1314	2	GTP-binding protein OBG family
263	M5005_Spy1089	M5005_Spy1089	1062802	1062930	-1	9	0.007751938	129	1	hypothetical protein
264	M5005_Spy1103	map	1076325	1077185	-1	8	0.00116144	861	1	methionine aminopeptidase
265	M5005_Spy1108	metK2	1081717	1082913	-1	213	0.005847953	1197	7	S-adenosylmethionine synthetase
266	M5005_Spy1110	birA	1086032	1086973	1	848	0.011677282	942	11	biotin operon repressor/biotin--[acetyl-CoA-carboxylase] synthetase
267	M5005_Spy1112	M5005_Spy1112	1087246	1088916	-1	323	0.002393776	1671	4	DNA polymerase III subunit gamma/tau
268	M5005_Spy1113	M5005_Spy1113	1088916	1089413	-1	101	0.008032129	498	4	GAF domain-containing protein
269	M5005_Spy1119	gapN	1094052	1095479	-1	0	0	1428	0	NADP-dependent glyceraldehyde-3-phosphate dehydrogenase
270	M5005_Spy1120	pstI	1095664	1097397	-1	32	0.007497116	1734	13	phosphoenolpyruvate-protein phosphotransferase
271	M5005_Spy1121	ptsH	1097402	1097665	-1	0	0	264	0	phosphocarrier protein HPr
272	M5005_Spy1122	nrdH	1098058	1098276	1	0	0	219	0	glutaredoxin
273	M5005_Spy1123	nrdE2	1098296	1100455	1	1	0.000462963	2160	1	ribonucleoside-diphosphate reductase alpha chain
274	M5005_Spy1124	nrdF2	1100788	1101747	1	32	0.003125	960	3	ribonucleoside-diphosphate reductase beta chain
275	M5005_Spy1132	alaS	1106469	1109087	-1	5	0.001145475	2619	3	alanyl-tRNA synthetase
276	M5005_Spy1145	sodA	1121336	1121941	-1	649	0.00990099	606	6	superoxide dismutase
277	M5005_Spy1146	M5005_Spy1146	1122038	1123078	-1	102	0.002881844	1041	3	DNA polymerase III
278	M5005_Spy1149	M5005_Spy1149	1126235	1126975	-1	529	0.004048583	741	3	1-acyl-sn-glycerol-3-phosphate acyltransferase
279	M5005_Spy1157	murF	1134782	1136173	-1	0	0	1392	0	UDP-N-acetylmuramoylalanine-D-glutamyl-lysine-- D-alanyl-D-alanine ligase
280	M5005_Spy1158	ddlA	1136364	1137410	-1	313	0.003820439	1047	4	D-alanine--D-alanine ligase
281	M5005_Spy1164	gpmA	1141140	1141835	-1	7	0.002873563	696	2	phosphoglycerate mutase
282	M5005_Spy1219	M5005_Spy1219	1176548	1176895	1	290	0.002873563	348	1	phage transcriptional regulator
283	M5005_Spy1230	M5005_Spy1230	1185007	1185834	-1	54	0.006038647	828	5	hemolysin
284	M5005_Spy1231	fps	1185827	1186699	-1	114	0.011454754	873	10	dimethylallyltransferase/geranyltranstransferase
285	M5005_Spy1234	folD	1188385	1189239	-1	21	0.003508772	855	3	methylenetetrahydrofolate dehydrogenase (NADP+)/methenyltetrahydrofolate cyclohydrolase
286	M5005_Spy1243	ileS	1198435	1201236	-1	744	0.002141328	2802	6	isoleucyl-tRNA synthetase
287	M5005_Spy1244	divIVAS	1201509	1202267	-1	0	0	759	0	cell division initiation protein
288	M5005_Spy1245	M5005_Spy1245	1202277	1203068	-1	0	0	792	0	RNA binding protein
289	M5005_Spy1246	M5005_Spy1246	1203068	1203322	-1	0	0	255	0	integral membrane protein
290	M5005_Spy1247	M5005_Spy1247	1203327	1203995	-1	0	0	669	0	putative cytosolic protein
291	M5005_Spy1248	M5005_Spy1248	1203995	1204666	-1	0	0	672	0	pyridoxal-5'-phosphate family protein
292	M5005_Spy1249	ftsZ	1204669	1205988	-1	0	0	1320	0	cell division protein
293	M5005_Spy1250	ftsA	1206012	1207376	-1	0	0	1365	0	cell division protein
294	M5005_Spy1251	divIB	1207588	1208736	-1	2	0.001740644	1149	2	cell division protein
295	M5005_Spy1252	murG	1208737	1209819	-1	1	0.000923361	1083	1	UDP-N-acetylglucosamine--N-acetylmuramyl- (pentapeptide) pyrophosphoryl-undecaprenol N-acetylglucosamine transferase
296	M5005_Spy1253	murD	1209819	1211177	-1	4	0.002943341	1359	4	UDP-N-acetylmuramoylalanine--D-glutamate ligase
297	M5005_Spy1255	typA	1211920	1213761	-1	49	0.002171553	1842	4	GTP-binding protein
298	M5005_Spy1267	M5005_Spy1267	1221692	1222183	-1	3	0.00203252	492	1	phosphopantetheine adenylyltransferase
299	M5005_Spy1292	valS	1248383	1250788	-1	2	0.000415628	2406	1	valyl-tRNA synthetase
300	M5005_Spy1318	rocA	1279496	1280851	-1	62	0.010324484	1356	14	sensory transduction protein kinase
301	M5005_Spy1321	M5005_Spy1321	1283842	1284375	1	4	0.001872659	534	1	putative cytosolic protein
302	M5005_Spy1330	M5005_Spy1330	1296432	1296809	-1	626	0.007936508	378	3	S1-type RNA-binding domain
303	M5005_Spy1332	yvqC	1298246	1298887	-1	237	0.014018692	642	9	two-component response regulator
304	M5005_Spy1335	M5005_Spy1335	1300696	1302594	-1	14	0.000526593	1899	1	serine/threonine protein kinase
305	M5005_Spy1336	pppL	1302591	1303331	-1	1	0.001349528	741	1	protein phosphatase 2C
306	M5005_Spy1338	M5005_Spy1338	1304681	1305616	-1	54	0.002136752	936	2	methionyl-tRNA formyltransferase
307	M5005_Spy1339	priA	1305678	1308062	-1	8	0.001677149	2385	4	primosomal protein N
308	M5005_Spy1340	M5005_Spy1340	1308127	1308444	-1	49	0.012578616	318	4	DNA-directed RNA polymerase omega chain
309	M5005_Spy1341	gmk	1308460	1309095	-1	0	0	636	0	guanylate kinase
310	M5005_Spy1342	M5005_Spy1342	1309205	1310812	-1	50	0.007462687	1608	12	hydrolase
311	M5005_Spy1352	M5005_Spy1352	1320559	1320885	-1	1	0.003058104	327	1	cell division initiation protein
312	M5005_Spy1353	M5005_Spy1353	1321007	1321522	-1	46	0.009689922	516	5	putative cytosolic protein
313	M5005_Spy1354	recU	1321603	1322202	1	3	0.001666667	600	1	recombination protein
314	M5005_Spy1355	pbp1A	1322189	1324354	1	2745	0.012003693	2166	26	multimodular transpeptidase-transglycosylase
315	M5005_Spy1357	M5005_Spy1357	1326343	1327167	-1	82	0.002424242	825	2	NH(3)-dependent NAD(+) synthetase
316	M5005_Spy1358	nadE	1327169	1328623	-1	20	0.002061856	1455	3	nicotinate phosphoribosyltransferase
317	M5005_Spy1360	M5005_Spy1360	1330342	1331259	-1	428	0.010893246	918	10	thioredoxin reductase
318	M5005_Spy1362	M5005_Spy1362	1331651	1332397	-1	245	0.005354752	747	4	transporter
319	M5005_Spy1363	M5005_Spy1363	1332394	1333197	-1	0	0	804	0	amino acid ABC transporter permease protein
320	M5005_Spy1364	M5005_Spy1364	1333392	1334735	-1	6	0.001488095	1344	2	ATP-dependent RNA helicase
321	M5005_Spy1365	M5005_Spy1365	1334893	1335903	-1	2	0.001978239	1011	2	phospho-N-acetylmuramoyl-pentapeptide- transferase
322	M5005_Spy1366	M5005_Spy1366	1335905	1338160	-1	2	0.000886525	2256	2	division specific D
323	M5005_Spy1367	ftsL	1338164	1338487	-1	33	0.00308642	324	1	cell division protein
324	M5005_Spy1368	mraW	1338492	1339505	-1	9	0.004930966	1014	5	S-adenosyl-methyltransferase
325	M5005_Spy1383	M5005_Spy1383	1355848	1356105	-1	214	0.011627907	258	3	putative cytosolic protein
326	M5005_Spy1384	glyS	1356259	1358298	-1	6	0.000980392	2040	2	glycyl-tRNA synthetase beta chain
327	M5005_Spy1385	glyQ	1358676	1359593	-1	1	0.001089325	918	1	glycyl-tRNA synthetase alpha chain
328	M5005_Spy1391	M5005_Spy1391	1365541	1366383	-1	75	0.013048636	843	11	degV family protein
329	M5005_Spy1409	infB	1379779	1382640	-1	0	0	2862	0	bacterial protein translation initiation factor 2
330	M5005_Spy1410	M5005_Spy1410	1382660	1382962	-1	0	0	303	0	LSU ribosomal protein L7AE
331	M5005_Spy1411	M5005_Spy1411	1382955	1383251	-1	0	0	297	0	putative cytosolic protein
332	M5005_Spy1412	nusA	1383267	1384424	-1	1	0.000863558	1158	1	N utilization substance protein A
333	M5005_Spy1413	M5005_Spy1413	1384599	1385135	-1	1	0.001862197	537	1	putative cytosolic protein
334	M5005_Spy1455	M5005_Spy1455	1420921	1421124	-1	3	0.009803922	204	2	phage protein
335	M5005_Spy1456	M5005_Spy1456	1421121	1421273	-1	17	0.013071895	153	2	phage protein
336	M5005_Spy1457	M5005_Spy1457	1421270	1421656	-1	2	0.005167959	387	2	phage protein
337	M5005_Spy1458	M5005_Spy1458	1421653	1421856	-1	0	0	204	0	phage protein
338	M5005_Spy1459	M5005_Spy1459	1421849	1422019	-1	1	0.005847953	171	1	phage protein
339	M5005_Spy1460	M5005_Spy1460	1422016	1422291	-1	1	0.003623188	276	1	phage protein
340	M5005_Spy1461	M5005_Spy1461	1422353	1422568	-1	0	0	216	0	phage protein
341	M5005_Spy1463	M5005_Spy1463	1423010	1423165	-1	3	0.006410256	156	1	phage protein
342	M5005_Spy1464	M5005_Spy1464	1423440	1423841	1	182	0.004975124	402	2	phage transcriptional regulator
343	M5005_Spy1469	M5005_Spy1469	1426829	1427620	-1	2	0.001262626	792	1	phosphotransferase enzyme family
344	M5005_Spy1476	M5005_Spy1476	1432183	1432644	-1	107	0.006493506	462	3	ATP/GTP hydrolase
345	M5005_Spy1483	serS	1439369	1440646	1	606	0.006259781	1278	8	seryl-tRNA synthetase
346	M5005_Spy1484	accD	1440868	1441638	-1	0	0	771	0	acetyl-coenzyme A carboxylase carboxyl transferase subunit alpha
347	M5005_Spy1485	accA	1441635	1442501	-1	0	0	867	0	acetyl-coenzyme A carboxylase carboxyl transferase subunit beta
348	M5005_Spy1486	accC	1442510	1443874	-1	1	0.000732601	1365	1	biotin carboxylase
349	M5005_Spy1487	fabZ	1443906	1444328	-1	0	0	423	0	(3R)-hydroxymyristoyl-[acyl carrier protein] dehydratase
350	M5005_Spy1488	accB	1444325	1444825	-1	4	0.003992016	501	2	biotin carboxyl carrier protein of acetyl-CoA carboxylase
351	M5005_Spy1489	fabF	1444827	1446059	-1	1	0.00081103	1233	1	3-oxoacyl-[acyl-carrier-protein] synthase
352	M5005_Spy1490	M5005_Spy1490	1446074	1446808	-1	1	0.001360544	735	1	3-oxoacyl-[acyl-carrier protein] reductase
353	M5005_Spy1491	fabD	1446798	1447736	-1	6	0.002129925	939	2	malonyl-CoA-[acyl-carrier-protein] transacylase
354	M5005_Spy1492	fabK	1447761	1448732	-1	0	0	972	0	enoyl-[acyl-carrier protein] reductase (NADH)
355	M5005_Spy1493	acpP	1448934	1449158	-1	0	0	225	0	acyl carrier protein
356	M5005_Spy1494	fabH	1449219	1450193	-1	0	0	975	0	3-oxoacyl-[acyl-carrier-protein] synthase III
357	M5005_Spy1495	M5005_Spy1495	1450194	1450628	-1	1	0.002298851	435	1	transcriptional regulator
358	M5005_Spy1496	phaB	1450705	1451496	-1	0	0	792	0	enoyl-CoA hydratase
359	M5005_Spy1497	dnaJ	1451716	1452873	-1	2394	0.006908463	1158	8	chaperone protein
360	M5005_Spy1498	dnaK	1453154	1454980	-1	14	0.001094691	1827	2	chaperone protein
361	M5005_Spy1499	grpE	1455161	1455694	-1	0	0	534	0	GrpE
362	M5005_Spy1500	hrcA	1455736	1456770	-1	31	0.004830918	1035	5	heat-inducible transcription repressor
363	M5005_Spy1506	gatB	1459972	1461411	-1	0	0	1440	0	aspartyl/glutamyl-tRNA(Asn/Gln) amidotransferase subunit B

364	M5005_Spy1507	gatA	1461411	1462877	-1	0	0	1467	0	aspartyl/glutamyl-tRNA(Asn/Gln) amidotransferase subunit A
365	M5005_Spy1508	gatC	1462877	1463179	-1	0	0	303	0	glutamyl-tRNA(Gln) amidotransferase subunit C
366	M5005_Spy1512	codY	1464875	1465657	-1	147	0.003831418	783	3	transcription pleiotropic repressor
367	M5005_Spy1532	acpS	1490763	1491863	-1	0	0	1101	0	alanine racemase
368	M5005_Spy1533	alr	1491860	1492216	-1	1	0.00280112	357	1	holo-[acyl-carrier protein] synthase
369	M5005_Spy1534	secA	1492332	1494851	-1	1	0.000396825	2520	1	protein translocase subunit
370	M5005_Spy1543	scrB	1503840	1505279	1	976	0.004166667	1440	6	sucrose-6-phosphate hydrolase
371	M5005_Spy1545	nusB	1506390	1506842	-1	14	0.008830022	453	4	N utilization substance protein B
372	M5005_Spy1546	M5005_Spy1546	1506835	1507224	-1	0	0	390	0	general stress protein
373	M5005_Spy1547	efp	1507270	1507827	-1	0	0	558	0	protein translation elongation factor P
374	M5005_Spy1551	corA	1512638	1513582	1	1	0.001058201	945	1	magnesium and cobalt transport protein
375	M5005_Spy1553	rpsR	1514504	1514743	-1	0	0	240	0	SSU ribosomal protein S18P
376	M5005_Spy1554	ssb3	1514908	1515399	-1	20	0.004065041	492	2	phage single-strand DNA binding protein
377	M5005_Spy1555	rpsF	1515421	1515711	-1	1	0.003436426	291	1	SSU ribosomal protein S6P
378	M5005_Spy1559	trx	1518214	1518528	-1	22	0.012698413	315	4	thioredoxin
379	M5005_Spy1564	M5005_Spy1564	1522614	1523516	1	1	0.00110742	903	1	ribonuclease HIII
380	M5005_Spy1565	spi	1523527	1524120	1	101	0.001683502	594	1	signal peptidase I
381	M5005_Spy1573	glpF2	1534322	1535170	-1	118	0.014134276	849	12	aquaporin
382	M5005_Spy1591	M5005_Spy1591	1549275	1550303	-1	1	0.000971817	1029	1	O-sialoglycoprotein endopeptidase
383	M5005_Spy1593	M5005_Spy1593	1550720	1551418	-1	2	0.001430615	699	1	glycoprotease protein family
384	M5005_Spy1594	M5005_Spy1594	1551703	1551933	1	0	0	231	0	putative transcriptional regulator
385	M5005_Spy1595	M5005_Spy1595	1551935	1553617	1	1	0.000594177	1683	1	Zn-dependent hydrolase
386	M5005_Spy1596	glnA	1553844	1555190	-1	477	0.01484781	1347	20	glutamine synthetase
387	M5005_Spy1597	M5005_Spy1597	1555228	1555599	-1	39	0.010752688	372	4	transcriptional regulator
388	M5005_Spy1599	pgk	1556480	1557676	-1	3	0.002506266	1197	3	phosphoglycerate kinase
389	M5005_Spy1602	M5005_Spy1602	1560080	1561744	-1	36	0.001801802	1665	3	putative kinase related to hydroxyacetone kinase
390	M5005_Spy1603	asp	1561744	1562109	-1	1	0.00273224	366	1	putative alkaline-shock protein
391	M5005_Spy1607	fba	1564157	1565038	-1	3	0.003401361	882	3	fructose-bisphosphate aldolase
392	M5005_Spy1616	thiD	1572679	1573443	-1	50	0.002614379	765	2	phosphomethylpyrimidine kinase/hydroxymethylpyrimidine kinase
393	M5005_Spy1646	rpsI	1606320	1606712	-1	0	0	393	0	SSU ribosomal protein S9P
394	M5005_Spy1647	rplM	1606733	1607179	-1	0	0	447	0	LSU ribosomal protein L13P
395	M5005_Spy1655	cysS	1611913	1613256	-1	543	0.005952381	1344	8	cysteinyl-tRNA synthetase
396	M5005_Spy1660	M5005_Spy1660	1615328	1617460	-1	226	0.009845288	2133	21	polyribonucleotide nucleotidyltransferase
397	M5005_Spy1666	M5005_Spy1666	1622576	1622845	-1	97	0.011111111	270	3	SSU ribosomal protein S15P
398	M5005_Spy1669	def	1623654	1624268	1	15	0.003252033	615	2	peptide deformylase
399	M5005_Spy1672	polC	1625513	1629910	-1	1	0.000227376	4398	1	DNA polymerase III alpha subunit
400	M5005_Spy1673	proS	1630165	1632021	-1	607	0.002154012	1857	4	prolyl-tRNA synthetase
401	M5005_Spy1674	M5005_Spy1674	1632219	1633478	-1	337	0.008730159	1260	11	pheromone-processing membrane metalloprotease
402	M5005_Spy1675	cdsA	1633551	1634345	-1	0	0	795	0	phosphatidate cytidylyltransferase
403	M5005_Spy1676	uppS	1634358	1635107	-1	0	0	750	0	undecaprenyl pyrophosphate synthetase
404	M5005_Spy1686	relA	1646250	1648469	-1	9	0.001801802	2220	4	GTP pyrophosphokinase/guanosine-3',5'-bis(diphosphate) 3'-pyrophosphohydrolase
405	M5005_Spy1690	M5005_Spy1690	1650149	1650631	1	285	0.014492754	483	7	nrdI.1
406	M5005_Spy1734	spi	1696679	1696984	-1	127	0.009803922	306	3	streptopain protease inhibitor
407	M5005_Spy1752	M5005_Spy1752	1711754	1711906	-1	0	0	153	0	LSU ribosomal protein L33P
408	M5005_Spy1761	groEL	1720962	1722593	-1	3	0.00122549	1632	2	60 kDa chaperonin
409	M5005_Spy1762	groES	1722629	1722961	-1	1	0.003003003	333	1	10 kDa chaperonin
410	M5005_Spy1765	csp	1726198	1726407	-1	0	0	210	0	cold shock protein
411	M5005_Spy1780	rpsB	1746110	1746877	1	2	0.001302083	768	1	SSU ribosomal protein S2P
412	M5005_Spy1781	M5005_Spy1781	1747011	1748051	1	18	0.003842459	1041	4	protein translation elongation factor Ts
413	M5005_Spy1796	M5005_Spy1796	1764579	1764998	-1	0	0	420	0	endonuclease involved in recombination
414	M5005_Spy1798	M5005_Spy1798	1765377	1765775	-1	1	0.002506266	399	1	arsenate reductase family protein
415	M5005_Spy1802	ruvA	1769201	1769797	-1	55	0.008375209	597	5	holliday junction DNA helicase
416	M5005_Spy1808	argS	1776862	1778451	1	2292	0.001257862	1590	2	arginyl-tRNA synthetase
417	M5005_Spy1812	M5005_Spy1812	1780687	1781628	-1	64	0.012738854	942	12	putative membrane spanning protein
418	M5005_Spy1813	aspS	1781621	1783369	-1	8	0.002858776	1749	5	aspartyl-tRNA synthetase
419	M5005_Spy1814	hisS	1783707	1784987	-1	1	0.00078064	1281	1	histidyl-tRNA synthetase
420	M5005_Spy1815	rpmF	1785207	1785389	1	33	0.010928962	183	2	LSU ribosomal protein L32P
421	M5005_Spy1831	rpsD	1797339	1797950	-1	2	0.001633987	612	1	SSU ribosomal protein S4P
422	M5005_Spy1834	M5005_Spy1834	1800006	1800278	-1	17	0.007326007	273	2	hypothetical protein
423	M5005_Spy1835	holB	1800295	1801662	-1	4	0.001461988	1368	2	replicative DNA helicase
424	M5005_Spy1838	gidA	1804208	1806106	-1	147	0.002106372	1899	4	glucose inhibited division protein A
425	M5005_Spy1840	trmU	1807324	1808445	-1	21	0.001782531	1122	2	tRNA (5-methylaminomethyl-2-thiouridylate)-methyltransferase
426	M5005_Spy1844	cbiQ	1811697	1812497	-1	1	0.001248439	801	1	cobalt transport protein
427	M5005_Spy1845	cbiO2	1812490	1813332	-1	0	0	843	0	cobalt transport ATP-binding protein
428	M5005_Spy1846	cbiO1	1813308	1814198	-1	0	0	891	0	cobalt transport ATP-binding protein
429	M5005_Spy1847	M5005_Spy1847	1814149	1814691	-1	2	0.001841621	543	1	CDP-diacylglycerol--glycerol-3-phosphate 3-phosphatidyltransferase
430	M5005_Spy1848	M5005_Spy1848	1814705	1815730	-1	0	0	1026	0	putative membrane associated protein
431	M5005_Spy1858	trsA	1827083	1828105	-1	57	0.002932551	1023	3	tryptophanyl-tRNA synthetase
432	M5005_Spy1859	M5005_Spy1859	1828197	1828322	-1	0	0	126	0	hypothetical protein
