# Supplementary material for: Novel Genes Required for the Fitness of Streptococcus pyogenes in Human Saliva
Source: mSphere. 2017 Nov 1;2(6):e00460-17. doi: 10.1128/mSphereDirect.00460-17 (PMC5663985; doi:10.1128/mSphereDirect.00460-17)
Supplement: TABLE S2 [file sph006172393st3.pdf]

TABLE S2 Gene mutations conferring decreased fitness after 12, 24 and 48 hour incubation with human saliva.
Columns I, j, K: comparing with other related genome-wide studies.
(a) Highlighted gene are found to be expressed in vivo in the monkey oropharynx. (Virtaneva et al, 2005)
(b) Homologous of highlighted genes are found to be required for Pneumococcus fitness in saliva. (Verhagen et al, 2014)
(c) Highlighted genes are found to be required for GAS fitness in human blood. (Le Breton, et al, 2013)

				Fitness in saliva, 12h	Fitness in saliva, 24h	Fitness in saliva, 48h			
**No.**	**locus_tag**	**gene_name**	**function**	**Fold-change**	**Fold-change**	**Fold-change**	**Expressed in NHP (a)**	**Pneumo fitness gene in saliva (b)**	**GAS fitness gene in blood (c)**
**1**	M5005_Spy0004	M5005_Spy0004	GTP-binding protein	-1.578970785	-1.58557353	-1.742569733	YES	YES	
**2**	M5005_Spy0019	recO	DNA repair protein			-1.754292769	YES		
**3**	M5005_Spy0085	M5005_Spy0085	putative DNA binding protein			-2.299023255	YES		
**4**	M5005_Spy0104	M5005_Spy0104	tRNA-dihydrouridine synthase			-3.304617837	YES	YES	
**5**	M5005_Spy0105	M5005_Spy0105	33 kDa chaperonin			-2.072901471	YES	YES	
**6**	M5005_Spy0122	M5005_Spy0122	putative DNA-binding protein			-2.341099429	YES		
**7**	M5005_Spy0249	oppA	oligopeptide-binding protein			-1.737837023	YES	YES	
**8**	M5005_Spy0251	oppC	oligopeptide transport system permease protein			-2.004743036	YES	YES	
**9**	M5005_Spy0253	oppF	oligopeptide transport ATP-binding protein			-1.626716488		YES	
**10**	M5005_Spy0301	M5005_Spy0301	integral membrane protein			-1.557764268			
**11**	M5005_Spy0327	upp	uracil phosphoribosyltransferase		-1.742587365	-13.55537654	YES		
**12**	M5005_Spy0382	msrA2	peptide methionine sulfoxide reductase		-1.695205174		YES		
**13**	M5005_Spy0401	M5005_Spy0401	putative cytosolic protein		-1.514687005	-1.676926824			
**14**	M5005_Spy0436	vicK	two-component sensor histidine kinase	-1.700538087	-1.684383807		YES		
**15**	M5005_Spy0448	M5005_Spy0448	hypothetical protein			-1.821000251	YES		
**16**	M5005_Spy0485	lgt	prolipoprotein diacylglyceryl transferase			-2.856429687	YES	YES	
**17**	M5005_Spy0486	M5005_Spy0486	hypothetical protein		-1.611614426	-2.522773501			
**18**	M5005_Spy0566	sagE	streptolysin S putative self-immunity protein			-1.848273909			
**19**	M5005_Spy0567	sagF	streptolysin S biosynthesis protein			-1.564898859	YES		
**20**	M5005_Spy0568	sagG	streptolysin S export ATP-binding protein	-13.89049981	-7.982236796	-12.05706834	YES		
**21**	M5005_Spy0569	sagH	streptolysin S export transmembrane protein	-11.5309895	-13.50127029	-750.0033792	YES		
**22**	M5005_Spy0570	sagI	streptolysin S export transmembrane protein	-10.44822569	-28.60167119	-11.59878722	YES		
**23**	M5005_Spy0611	M5005_Spy0611	hypothetical protein			-1.671569461	YES		
**24**	M5005_Spy0630	nifS1	cysteine desulfhydrase		-1.723983908	-22.42314591	YES		
**25**	M5005_Spy0631	thiI	thiamine biosynthesis protein		-1.855500353	-24.08682761	YES		
**26**	M5005_Spy0642	carA	carbamoyl-phosphate synthase small chain			-1.549580679	YES		YES
**27**	M5005_Spy0643	carB	carbamoyl-phosphate synthase large chain			-1.631651082	YES		YES
**28**	M5005_Spy0644	M5005_Spy0644	periplasmic component of efflux system	-2.252987968	-2.29658991	-2.910849618			
**29**	M5005_Spy0645	M5005_Spy0645	ABC transporter ATP-binding protein	-1.87635273	-2.005171629	-2.721826223	YES		
**30**	M5005_Spy0646	M5005_Spy0646	ABC transporter permease protein	-2.16470395	-2.115199827	-2.999056121	YES		
**31**	M5005_Spy0665	M5005_Spy0665	transposase			-1.577993436			
**32**	M5005_Spy0693	M5005_Spy0693	hypothetical protein			-1.605451025	YES		
**33**	M5005_Spy0718	M5005_Spy0718	hypothetical protein			-1.588522263	YES		
**34**	M5005_Spy0722	miaA	tRNA delta(2)-isopentenylpyrophosphate transferase			-1.862764877	YES		
**35**	M5005_Spy0805	srtK	nisin biosynthesis sensor protein			-1.508429525	YES		
**36**	M5005_Spy0810	srtG	lantibiotic transport permease protein			-1.661863515			
**37**	M5005_Spy0817	dacA1	D-alanyl-D-alanine carboxypeptidase			-1.565288324	YES		
**38**	M5005_Spy0826	potA	spermidine/putrescine transport system ATP-binding protein	-1.521799626		-3.088251227	YES		
**39**	M5005_Spy0829	M5005_Spy0829	spermidine/putrescine-binding protein			-1.946753612			YES
**40**	M5005_Spy0841	M5005_Spy0841	glutamine amidotransferase			-1.633038038	YES		
**41**	M5005_Spy0847	M5005_Spy0847	GTP pyrophosphokinase	-1.697639654	-1.556951387				
**42**	M5005_Spy0860	apbE	thiamine biosynthesis lipoprotein	-1.535387549	-2.002338933		YES		
**43**	M5005_Spy0864	hemK	peptide release factor-glutamine N5-methyltransferase	-1.725261326			YES		
**44**	M5005_Spy0872	nox	NADH oxidase H2O-forming			-1.514291312	YES		
**45**	M5005_Spy0926	M5005_Spy0926	cardiolipin synthetase	-3.061011447	-5.999314076	-22.92056556	YES	YES	
**46**	M5005_Spy0944	M5005_Spy0944	16S rRNA m(2)G 1207 methyltransferase			-1.537389119	YES		
**47**	M5005_Spy0951	pstB	phosphate transport ATP-binding protein		-3.030859923	-7.879985046		YES	
**48**	M5005_Spy0952	pstB2	phosphate transport ATP-binding protein	-1.636657929	-3.627287832	-8.219908713	YES	YES	
**49**	M5005_Spy0953	pstA	phosphate transport system permease protein	-1.64675359	-3.71529891	-12.6715751		YES	
**50**	M5005_Spy0954	pstC	phosphate transport system permease protein	-1.702665738	-3.822470575	-13.28351681	YES	YES	
**51**	M5005_Spy0955	pstS	phosphate-binding protein	-1.609345238	-4.081100056	-10.69078939	YES	YES	
**52**	M5005_Spy0971	M5005_Spy0971	general stress protein, Gls24 family		-1.658159678	-2.48787223	YES		
**53**	M5005_Spy0973	M5005_Spy0973	general stress protein, Gls24 family		-1.736815972	-1.903457746	YES		
**54**	M5005_Spy0975	M5005_Spy0975	hypothetical protein	-1.770147517	-1.904237938	-3.605469009	YES		
**55**	M5005_Spy0987	sipC	signal peptidase I		-1.669798494	-3.583806216	YES		
**56**	M5005_Spy0992	M5005_Spy0992	ABC transporter ATP-binding protein	-2.35058252	-2.001534524	-2.847001093			
**57**	M5005_Spy0993	M5005_Spy0993	ABC transporter permease protein	-2.794310569	-2.144272007	-2.784840361	YES		
**58**	M5005_Spy1021	M5005_Spy1021	phage protein			-1.541202391			
**59**	M5005_Spy1023	M5005_Spy1023	terminase large subunit			-1.553884241			
**60**	M5005_Spy1067	malX	maltose/maltodextrin-binding protein			-1.896662761	YES		
**61**	M5005_Spy1068	M5005_Spy1068	transposase		-1.511512107	-1.886032564			
**62**	M5005_Spy1075	uvrB	excinuclease ABC subunit B		-1.530782126		YES		
**63**	M5005_Spy1107	murZ	UDP-N-acetylglucosamine 1-carboxyvinyl transferase	-1.64501845			YES		
**64**	M5005_Spy1139	nagB	glucosamine-6-phosphate isomerase	-1.845643238	-4.268789759	-18.29865104	YES		
**65**	M5005_Spy1182	M5005_Spy1182	phage protein			-1.660660452	YES		
**66**	M5005_Spy1226	M5005_Spy1226	degV family protein			-1.548113507		YES	
**67**	M5005_Spy1242	M5005_Spy1242	putative cytosolic protein	-2.396844911	-14.18022438	-195.1506992	YES		
**68**	M5005_Spy1273	arcB	ornithine carbamoyltransferase			-2.494984006	YES		
**69**	M5005_Spy1325	M5005_Spy1325	ribosome-associated factor Y			-2.14292978			
**70**	M5005_Spy1375	tkt	transketolase	-3.496674695	-5.730245502	-15.59097809	YES	YES	
**71**	M5005_Spy1381	glpK	glycerol kinase	-1.535859342			YES		
**72**	M5005_Spy1388	nagA	N-acetylglucosamine-6-phosphate deacetylase	-1.930321315	-7.819520774	-148.3093522	YES	YES	
**73**	M5005_Spy1402	lacR1	lactose phosphotransferase system repressor			-2.57840735	YES		
**74**	M5005_Spy1418	M5005_Spy1418	phage protein		-1.501932821				
**75**	M5005_Spy1439	M5005_Spy1439	portal protein		-2.064830788	-2.713178276	YES		
**76**	M5005_Spy1468	M5005_Spy1468	tRNA (m(7)G46) methyltransferase	-1.588241472	-23.12331138	-8197.940314	YES		
**77**	M5005_Spy1472	hit	bis(5'-nucleosyl)-tetraphosphatase (asymmetrical)		-1.681582108	-2.315088687	YES		
**78**	M5005_Spy1473	M5005_Spy1473	hypothetical protein			-1.965495543			
**79**	M5005_Spy1479	manL	PTS system			-1.627000908	YES		
**80**	M5005_Spy1480	manM	PTS system			-1.915777282			
**81**	M5005_Spy1481	manN	PTS system			-1.788676679	YES		YES
**82**	M5005_Spy1519	recG	ATP-dependent DNA helicase	-2.785545672	-2.291381227	-2.168312974	YES		
**83**	M5005_Spy1560	M5005_Spy1560	phosphatidylglycerophosphatase B		-1.51728074	-2.310177493	YES		
**84**	M5005_Spy1719	emm1.0	M protein			-1.523545262	YES		
**85**	M5005_Spy1737	rgg	transcriptional regulator			-1.524186201			
**86**	M5005_Spy1753	pbp2A	multimodular transpeptidase-transglycosylase	-2.939159969	-2.808870479	-3.251429026	YES	YES	
**87**	M5005_Spy1769	ahpF	peroxiredoxin reductase (NAD(P)H)			-3.048317251			
**88**	M5005_Spy1770	hutI	imidazolonepropionase			-1.578855117	YES		
**89**	M5005_Spy1783	dexS	trehalose-6-phosphate hydrolase			-2.283622667	YES	YES	
**90**	M5005_Spy1810	M5005_Spy1810	putative membrane spanning protein			-3.920793404	YES		
**91**	M5005_Spy1823	M5005_Spy1823	integral membrane protein			-1.887367852	YES		
**92**	M5005_Spy1837	M5005_Spy1837	phosphoesterase	-3.74576977	-18.70421192	-163.915988			
							Sum = 68	Sum = 18	Sum = 4
