# Supplementary material for: Novel Genes Required for the Fitness of Streptococcus pyogenes in Human Saliva
Source: mSphere. 2017 Nov 1;2(6):e00460-17. doi: 10.1128/mSphereDirect.00460-17 (PMC5663985; doi:10.1128/mSphereDirect.00460-17)
Supplement: TABLE S3 [file sph006172393st4.pdf]

TABLE S3 Gene mutations conferring increased fitness after 12, 24 and 48 hour incubation with human saliva.

No.	locus_tag	gene name	function	Fitness in saliva, 12h Fold-change	Fitness in saliva, 24h Fold-change	Fitness in saliva, 48h Fold-change
1	M5005_Spy0024	purF	amidophosphoribosyltransferase		1.546287816	1.620231323
2	M5005_Spy0077	adcR	transcriptional regulator, MarR family			1.866940197
3	M5005_Spy0153	araD	L-ribulose-5-phosphate 4-epimerase			1.560436139
4	M5005_Spy0160	M5005_Spy0160	CoA binding protein			1.514312702
5	M5005_Spy0196	M5005_Spy0196	multidrug resistance ABC transporter ATP-binding and permease protein		1.660198805	2.046981293
6	M5005_Spy0197	M5005_Spy0197	multidrug resistance ABC transporter ATP-binding and permease protein		1.501380503	1.650296122
7	M5005_Spy0201	M5005_Spy0201	carbonic anhydrase		1.520079201	1.84580509
8	M5005_Spy0218	M5005_Spy0218	N-acetylmannosamine kinase			1.517044391
9	M5005_Spy0247	M5005_Spy0247	D-alanyl-D-alanine carboxypeptidase			1.714854011
10	M5005_Spy0249	oppA	oligopeptide-binding protein		1.502346643	
11	M5005_Spy0250	oppB	oligopeptide transport system permease protein	1.524493622		
12	M5005_Spy0252	oppD	oligopeptide transport ATP-binding protein		1.62410597	
13	M5005_Spy0271	M5005_Spy0271	ABC transporter substrate-binding protein			1.634038266
14	M5005_Spy0272	M5005_Spy0272	ABC transporter ATP-binding protein			1.504571957
15	M5005_Spy0273	M5005_Spy0273	ABC transporter permease protein			1.526467992
16	M5005_Spy0300	M5005_Spy0300	hydrolase, HAD superfamily			2.329319634
17	M5005_Spy0408	fpg	formamidopyrimidine-DNA glycosylase			1.706845388
18	M5005_Spy0414	M5005_Spy0414	exoribonuclease II			1.57886081
19	M5005_Spy0418	M5005_Spy0418	permease		1.65667103	2.290059255
20	M5005_Spy0536	dinG	ATP-dependent helicase			1.815809616
21	M5005_Spy0539	M5005_Spy0539	ATP-binding protein		1.616159273	2.323902508
22	M5005_Spy0701	cpsY	transcriptional regulator, LysR family		1.55009938	
23	M5005_Spy0751	acoA	pyruvate dehydrogenase E1 component alpha subunit	2.626460759	2.543422225	
24	M5005_Spy0752	acoB	pyruvate dehydrogenase E1 component beta subunit	2.246309539	2.463080347	
25	M5005_Spy0781	ptsB	PTS system, mannose/fructose family IIB component			1.528029417
26	M5005_Spy0872	nox	NADH oxidase H2O-forming	1.585734715		
27	M5005_Spy0939	M5005_Spy0939	nucleoside transport system permease protein	1.648182329	1.696950246	2.682402799
28	M5005_Spy0940	M5005_Spy0940	nucleoside transport system permease protein	1.651275744	1.751156511	2.872360691
29	M5005_Spy0941	M5005_Spy0941	nucleoside transport ATP-binding protein	1.661844082	1.671303782	2.764041275
30	M5005_Spy0942	M5005_Spy0942	nucleoside-binding protein			2.163432682
31	M5005_Spy0943	cdd	cytidine deaminase		1.523048766	2.209350977
32	M5005_Spy1056	malM	4-alpha-glucanotransferase			1.914275724
33	M5005_Spy1061	M5005_Spy1061	transcriptional regulator, LacI family			1.534836719
34	M5005_Spy1307	M5005_Spy1307	putative membrane spanning protein			1.79063151
35	M5005_Spy1399	M5005_Spy1399	PTS system, galactose-specific IIC component		1.954230725	1.723014989
36	M5005_Spy1400	M5005_Spy1400	PTS system, galactose-specific IIB component		1.70092435	
37	M5005_Spy1401	M5005_Spy1401	PTS system, galactose-specific IIA component		1.680429205	
38	M5005_Spy1431	M5005_Spy1431	phage protein			1.596824012
39	M5005_Spy1527	M5005_Spy1527	ferrichrome transport system permease protein			1.534785447
40	M5005_Spy1538	pmi	mannose-6-phosphate isomerase			1.546254532
41	M5005_Spy1544	scrR	sucrose operon repressor	1.665180925		
42	M5005_Spy1612	ropA	trigger factor, ppiase			1.625575913
43	M5005_Spy1682	msmK	multiple sugar transport ATP-binding protein			1.738151628
44	M5005_Spy1683	lrp	leucine rich protein			1.667179221
45	M5005_Spy1692	M5005_Spy1692	PTS system, glucose-specific IIABC component			1.512690523
46	M5005_Spy1699	M5005_Spy1699	ATPase, AAA family			1.60618087
47	M5005_Spy1726	M5005_Spy1726	ABC transporter permease protein			1.620490287
48	M5005_Spy1727	M5005_Spy1727	ABC transporter ATP-binding protein			1.510127286
49	M5005_Spy1728	M5005_Spy1728	periplasmic component of efflux system		1.512973768	1.77877113
50	M5005_Spy1754	M5005_Spy1754	translation initiation inhibitor		1.601589967	
51	M5005_Spy1807	argR2	arginine repressor			1.661303945
52	M5005_Spy1818	cadC	cadmium efflux system accessory protein		1.501812564	
53	M5005_Spy1857	guaB	inosine-5'-monophosphate dehydrogenase			1.843457623
