# Supplementary material for: Novel Genes Required for the Fitness of Streptococcus pyogenes in Human Saliva
Source: mSphere. 2017 Nov 1;2(6):e00460-17. doi: 10.1128/mSphereDirect.00460-17 (PMC5663985; doi:10.1128/mSphereDirect.00460-17)
Supplement: TABLE S4 [file sph006172393st5.pdf]

TABLE S4 Fitness score of each gene in the M1 GAS genome after 12 hour saliva incubation.

locus_tag	gene_name	function	Fitness score*	q.value
M5005_Spy0004	M5005_Spy0004	GTP-binding protein	-0.658984478	0.004613522
M5005_Spy0006	trcF	transcription-repair coupling factor	0.152731351	0.022813858
M5005_Spy0014	M5005_Spy0014	amino acid permease	-0.244870632	0.002004011
M5005_Spy0023	M5005_Spy0023	phosphoribosylformylglycinamidine synthase	0.136661559	0.08100213
M5005_Spy0024	purF	amidophosphoribosyltransferase	0.142213903	0.086681958
M5005_Spy0026	purN	phosphoribosylglycinamide formyltransferase	0.231280796	0.056654458
M5005_Spy0027	M5005_Spy0027	phosphoribosylaminoimidazolecarboxamide formyltransferase/IMP cyclohydrolase	0.194261249	0.008249929
M5005_Spy0029	purD	phosphoribosylamine--glycine ligase	0.220085098	0.005805314
M5005_Spy0033	purB	adenylosuccinate lyase	0.190920835	0.031026832
M5005_Spy0035	ruvB	holliday junction DNA helicase	-5.355174669	0.001023002
M5005_Spy0077	adcR	transcriptional regulator, MarR family	0.326748966	0.088595213
M5005_Spy0082	pbp1b	multimodular transpeptidase-transglycosylase PBP 1B	-0.493376495	0.081891422
M5005_Spy0093	M5005_Spy0093	adenine-specific methyltransferase	0.169261005	0.016052162
M5005_Spy0096	proC	pyrroline-5-carboxylate reductase	0.182919464	0.081163703
M5005_Spy0097	pepA	glutamyl aminopeptidase	0.183742746	0.038729426
M5005_Spy0103	M5005_Spy0103	deoxyadenosine kinase/deoxyguanosine kinase	0.211792279	0.044760874
M5005_Spy0109	M5005_Spy0109	fibronectin-binding protein	0.156437696	0.019843125
M5005_Spy0113	M5005_Spy0113	transposase	0.177952721	0.061179099
M5005_Spy0114	M5005_Spy0114	sortase	0.154083309	0.099426552
M5005_Spy0122	M5005_Spy0122	putative DNA-binding protein	0.271197351	0.009825821
M5005_Spy0124	sloR	transcriptional regulator	0.185873602	0.013162572
M5005_Spy0213	M5005_Spy0213	N-acetylneuraminate-binding protein	0.197142087	0.062727687
M5005_Spy0224	rpe	ribulose-phosphate 3-epimerase	-0.69581883	0.016276004
M5005_Spy0225	M5005_Spy0225	thiamin pyrophosphokinase	-0.883112446	1.36926E-06
M5005_Spy0238	bacA	putative undecaprenol kinase	0.218714424	0.079609375
M5005_Spy0240	M5005_Spy0240	undecaprenyl-phosphate alpha-N-acetylglucosaminephosphotransferase	-0.999169025	1.16426E-18
M5005_Spy0241	rgpG	putative cytosolic protein	-0.696209772	3.49051E-10
M5005_Spy0245	nifU	iscU protein	-0.315974812	0.08530506
M5005_Spy0246	M5005_Spy0246	ABC transporter-associated protein	-0.217922814	0.094749046
M5005_Spy0247	M5005_Spy0247	D-alanyl-D-alanine carboxypeptidase	0.514367681	5.69884E-13
M5005_Spy0249	oppA	oligopeptide-binding protein	0.538718209	0.000276915
M5005_Spy0250	oppB	oligopeptide transport system permease protein	0.608330115	0.000267604
M5005_Spy0252	oppD	oligopeptide transport ATP-binding protein	0.529175008	0.002308473
M5005_Spy0265	M5005_Spy0265	iojap protein family	0.250462218	0.035573155
M5005_Spy0288	snf	SWF/SNF family helicase	-0.436535236	1.10495E-07
M5005_Spy0292	M5005_Spy0292	aminodeoxychorismate lyase family	-1.398021069	0.008792642
M5005_Spy0297	M5005_Spy0297	transposase	0.187312822	0.090939043
M5005_Spy0308	M5005_Spy0308	putative cytosolic protein	-0.577351766	0.000134024
M5005_Spy0317	hlyX	putative hemolysin	-0.208183855	0.023585478
M5005_Spy0319	ppaC	manganese-dependent inorganic pyrophosphatase	-2.077421416	0.000704562
M5005_Spy0339	exoA	exodeoxyribonuclease III	-0.510757625	0.000185678
M5005_Spy0362	gcaD	glucosamine-1-phosphate acetyltransferase/UDP-N-acetylglucosamine pyrophosphorylase	-0.713616392	0.049213846
M5005_Spy0364	M5005_Spy0364	putative membrane associated protein	-0.618042214	0.013513682
M5005_Spy0384	M5005_Spy0384	surface antigen	-0.225638512	0.008249929
M5005_Spy0420	M5005_Spy0420	glucosyltransferase	-0.271151013	0.008775124
M5005_Spy0436	vicK	two-component sensor histidine kinase	-0.765991318	1.83718E-08
M5005_Spy0439	smc	chromosome partition protein	-0.182969047	0.002909828
M5005_Spy0470	M5005_Spy0470	hydrolase, HAD superfamily	0.144496461	0.099312082
M5005_Spy0486	M5005_Spy0486	hypothetical protein	-0.278131347	0.000105206
M5005_Spy0499	M5005_Spy0499	thiamine transporter	0.582643028	0.002374227
M5005_Spy0516	pacL	calcium-transporting ATPase	-1.832712959	0.010438289
M5005_Spy0532	ftsX	cell division protein	-3.095206603	0.000249808
M5005_Spy0554	M5005_Spy0554	septation ring formation regulator	1.073526865	0.026587383
M5005_Spy0568	sagG	streptolysin S export ATP-binding protein	-3.796026607	1.51349E-38
M5005_Spy0569	sagH	streptolysin S export transmembrane protein	-3.527444414	1.62459E-32
M5005_Spy0570	sagI	streptolysin S export transmembrane protein	-3.385186061	1.63633E-22
M5005_Spy0609	M5005_Spy0609	phosphoglycerol transferase	-0.303195648	2.22767E-05
M5005_Spy0623	M5005_Spy0623	methyltransferase	-0.311312085	0.026274714
M5005_Spy0624	aroD	3-dehydroquinate dehydratase	-0.893875389	0.006605639
M5005_Spy0628	folC2	folylpolyglutamate synthase/dihydrofolate synthase	0.263048013	0.016342543
M5005_Spy0642	carA	carbamoyl-phosphate synthase small chain	-0.308985613	8.46644E-06
M5005_Spy0643	carB	carbamoyl-phosphate synthase large chain	-0.281185035	3.26425E-08
M5005_Spy0644	M5005_Spy0644	periplasmic component of efflux system	-1.171839609	7.71762E-93
M5005_Spy0645	M5005_Spy0645	ABC transporter ATP-binding protein	-0.907931061	2.42106E-41
M5005_Spy0646	M5005_Spy0646	ABC transporter permease protein	-1.114169732	5.3171E-92
M5005_Spy0657	trxB	thioredoxin reductase	-0.950555933	0.08100213
M5005_Spy0660	fruR	fructose repressor	-0.221569848	0.063452252
M5005_Spy0661	fruB	1-phosphofructokinase	-0.400752833	0.016577104
M5005_Spy0662	fruA	PTS system, fructose-specific IIABC component	-0.254036683	0.099426552
M5005_Spy0663	mur11	autolysin	-0.180057752	0.053747552
M5005_Spy0679	M5005_Spy0679	GTP pyrophosphokinase	-0.188759942	0.095871803
M5005_Spy0693	M5005_Spy0693	hypothetical protein	0.284637649	4.69155E-08
M5005_Spy0715	M5005_Spy0715	SSU ribosomal protein S1P	1.756768776	0.031196792
M5005_Spy0728	apt	adenine phosphoribosyltransferase	1.346037774	3.37325E-15
M5005_Spy0740	fbp	fibronectin-binding protein	-0.22699608	0.016396718
M5005_Spy0743	M5005_Spy0743	ABC transporter substrate-binding protein	-0.181541268	0.038801584
M5005_Spy0750	M5005_Spy0750	ABC transporter ATP-binding protein	-0.195195149	0.003735527
M5005_Spy0751	acoA	pyruvate dehydrogenase E1 component alpha subunit	1.39312003	1.51782E-12
M5005_Spy0752	acoB	pyruvate dehydrogenase E1 component beta subunit	1.167556743	1.20726E-05
M5005_Spy0758	lplB	lipoate-protein ligase A	0.389265511	0.06630619
M5005_Spy0763	femD	phosphoglucosamine mutase	0.380717368	0.070516093
M5005_Spy0767	M5005_Spy0767	4-nitrophenylphosphatase	0.233209459	0.03960479

M5005_Spy0791	uvrC	excinuclease ABC subunit C	-0.174380248	0.016052162
M5005_Spy0817	dacA1	D-alanyl-D-alanine carboxypeptidase	-0.240331633	0.003926208
M5005_Spy0818	M5005_Spy0818	polysaccharide deacetylase	0.180919149	0.041299878
M5005_Spy0826	potA	spermidine/putrescine transport system ATP-binding protein	-0.605778413	0.099312082
M5005_Spy0838	M5005_Spy0838	lipase/acylhydrolase family protein	0.277051007	0.000180968
M5005_Spy0847	M5005_Spy0847	GTP pyrophosphokinase	-0.763530261	0.021222749
M5005_Spy0860	apbE	thiamine biosynthesis lipoprotein	-0.618602854	0.08270092
M5005_Spy0864	hemK	peptide release factor-glutamine N5-methyltransferase	-0.786814904	0.099739058
M5005_Spy0869	M5005_Spy0869	antigen	-0.521323456	0.073355276
M5005_Spy0872	nox	NADH oxidase H2O-forming	0.665151436	2.07049E-18
M5005_Spy0898	M5005_Spy0898	2-(5''-triphosphoribosyl)-3'-dephosphocoenzyme-A synthase	0.158426349	0.061166736
M5005_Spy0906	citE	citrate lyase beta chain/citryl-CoA lyase subunit	0.201673656	0.002822296
M5005_Spy0907	citF	citrate lyase alpha chain/citrate CoA-transferase	0.24846206	0.001334037
M5005_Spy0925	rnhB	anaerobic ribonucleoside-triphosphate reductase	-0.294058452	0.028749554
M5005_Spy0926	M5005_Spy0926	cardiolipin synthetase	-1.61400844	4.79854E-06
M5005_Spy0934	M5005_Spy0934	lipoate-protein ligase A	0.174130379	0.056654458
M5005_Spy0937	M5005_Spy0937	transporter	-0.838221881	0.01157833
M5005_Spy0939	M5005_Spy0939	nucleoside transport system permease protein	0.720875848	6.61781E-13
M5005_Spy0940	M5005_Spy0940	nucleoside transport system permease protein	0.723581053	3.27732E-14
M5005_Spy0941	M5005_Spy0941	nucleoside transport ATP-binding protein	0.732785031	9.71289E-16
M5005_Spy0942	M5005_Spy0942	nucleoside-binding protein	0.548132533	1.64562E-07
M5005_Spy0943	cdd	cytidine deaminase	0.555968977	4.60928E-10
M5005_Spy0949	pepN	lysyl aminopeptidase/alanine aminopeptidase	0.152310323	0.052092414
M5005_Spy0950	phoU	phosphate transport system protein	1.724668039	0.026587383
M5005_Spy0951	pstB	phosphate transport ATP-binding protein	-0.570522025	4.16896E-16
M5005_Spy0952	pstB2	phosphate transport ATP-binding protein	-0.710752821	4.12889E-12
M5005_Spy0953	pstA	phosphate transport system permease protein	-0.719624695	1.35441E-24
M5005_Spy0954	pstC	phosphate transport system permease protein	-0.767795237	1.10315E-15
M5005_Spy0955	pstS	phosphate-binding protein	-0.686473847	5.42317E-30
M5005_Spy0970	M5005_Spy0970	NAD-dependent K+ or Na+ uptake system component	-1.010507847	0.005041791
M5005_Spy0974	M5005_Spy0974	small integral membrane protein	-0.616659397	0.099426552
M5005_Spy0975	M5005_Spy0975	hypothetical protein	-0.823869594	9.9717E-06
M5005_Spy0987	sipC	signal peptidase I	-0.521183455	3.60956E-15
M5005_Spy0992	M5005_Spy0992	ABC transporter ATP-binding protein	-1.233018329	6.58789E-19
M5005_Spy0993	M5005_Spy0993	ABC transporter permease protein	-1.482492376	1.16426E-18
M5005_Spy1007	M5005_Spy1007	phage protein	-0.13421468	0.062727687
M5005_Spy1023	M5005_Spy1023	terminase large subunit	0.16483824	0.051754079
M5005_Spy1032	M5005_Spy1032	phage protein	-0.520937834	0.029146179
M5005_Spy1044	M5005_Spy1044	phage protein	0.26785544	0.028749554
M5005_Spy1050	M5005_Spy1050	phage transcriptional repressor	-0.81426741	0.00104902
M5005_Spy1055	glgP	glycogen phosphorylase	-0.464464399	2.53003E-20
M5005_Spy1056	malM	4-alpha-glucanotransferase	0.190946321	0.000943514
M5005_Spy1057	malR	transcriptional regulator, LacI family	0.318576906	6.94701E-06
M5005_Spy1060	malG	maltose transport system permease protein	-0.158650552	0.030264184
M5005_Spy1066	amyB	neopullulanase/cyclomaltodextrinase/maltogenic alpha-amylase	-0.319985153	7.87989E-08
M5005_Spy1068	M5005_Spy1068	transposase	-0.283566789	0.009170186
M5005_Spy1070	dltD	protein precursor	-1.239407079	0.070029144
M5005_Spy1075	uvrB	excinuclease ABC subunit B	-0.434689982	5.03382E-06
M5005_Spy1095	M5005_Spy1095	putative cytosolic protein	0.290339619	3.13717E-05
M5005_Spy1099	M5005_Spy1099	transcriptional regulator, LytR family	-0.252337491	2.79164E-05
M5005_Spy1107	murZ	UDP-N-acetylglucosamine 1-carboxyvinyl transferase	-0.718103764	0.03062196
M5005_Spy1108	metK2	S-adenosylmethionine synthetase	-1.049545437	0.010766061
M5005_Spy1139	nagB	glucosamine-6-phosphate isomerase	-0.884123707	7.22728E-20
M5005_Spy1226	M5005_Spy1226	degV family protein	-0.211590973	0.049087055
M5005_Spy1229	argR1	arginine repressor	-0.310737406	0.013674358
M5005_Spy1233	M5005_Spy1233	exodeoxyribonuclease VII large subunit	-0.428534745	0.000490899
M5005_Spy1240	clpE	ATP-dependent clp protease ATP-binding subunit	-0.338893935	2.63157E-08
M5005_Spy1242	M5005_Spy1242	putative cytosolic protein	-1.261136562	3.05751E-07
M5005_Spy1266	M5005_Spy1266	ATP-dependent protease La	-0.853753259	0.09702059
M5005_Spy1272	M5005_Spy1272	arginine/ornithine antiporter	0.228811713	0.002463814
M5005_Spy1275	arcA	arginine deiminase	0.186307456	0.08270092
M5005_Spy1314	hyl	hyaluronoglucosaminidase	-0.175893731	0.035015208
M5005_Spy1316	M5005_Spy1316	hypothetical protein	-0.399198411	3.79037E-07
M5005_Spy1317	M5005_Spy1317	alpha-mannosidase	-0.463215344	6.44134E-11
M5005_Spy1319	M5005_Spy1319	tRNA (uracil-5-)-methyltransferase	0.12802925	0.099877694
M5005_Spy1326	comFC	COMF operon protein 3	-0.189249638	0.072289245
M5005_Spy1328	M5005_Spy1328	Xaa-Pro dipeptidase	0.193375186	0.057161218
M5005_Spy1333	yvqE	two-component sensor protein	0.232802719	0.081304282
M5005_Spy1348	M5005_Spy1348	D-beta-hydroxybutyrate permease	0.191391351	0.007044961
M5005_Spy1349	luxS	autoinducer-2 production protein	0.292587869	0.001995711
M5005_Spy1356	pepC	aminopeptidase C	0.215706779	0.00315439
M5005_Spy1375	tkt	transketolase	-1.805983584	3.53596E-17
M5005_Spy1380	glpO	alpha-glycerophosphate oxidase	0.192995821	0.053747552
M5005_Spy1381	glpK	glycerol kinase	-0.619046096	1.0811E-11
M5005_Spy1388	nagA	N-acetylglucosamine-6-phosphate deacetylase	-0.948841014	2.40063E-21
M5005_Spy1390	M5005_Spy1390	hypothetical protein	-0.318840945	0.09702059
M5005_Spy1399	M5005_Spy1399	PTS system, galactose-specific IIC component	0.466679867	8.21418E-11
M5005_Spy1400	M5005_Spy1400	PTS system, galactose-specific IIB component	0.583244158	1.16426E-18
M5005_Spy1401	M5005_Spy1401	PTS system, galactose-specific IIA component	0.445454846	3.82185E-06
M5005_Spy1402	lacR1	lactose phosphotransferase system repressor	0.258699263	0.000633075
M5005_Spy1421	M5005_Spy1421	phage infection protein	-0.186223164	0.016577104
M5005_Spy1423	M5005_Spy1423	hyaluronoglucosaminidase	-0.196297895	0.027136065
M5005_Spy1424	M5005_Spy1424	phage endopeptidase	-0.206008503	0.07644842
M5005_Spy1439	M5005_Spy1439	portal protein	-0.432535035	2.97587E-17
M5005_Spy1444	M5005_Spy1444	adenine-specific methyltransferase	-0.393676418	0.000108112

M5005_Spy1447	M5005_Spy1447	phage-related DNA helicase	-0.255635751	0.057161218
M5005_Spy1450	M5005_Spy1450	phage-encoded DNA polymerase	-0.412528601	0.005461935
M5005_Spy1452	M5005_Spy1452	phage protein	-0.870685732	0.072289245
M5005_Spy1453	M5005_Spy1453	phage protein	-0.320661979	0.038418056
M5005_Spy1464	M5005_Spy1464	phage transcriptional regulator, Cro/CI family	-1.254954762	0.006097375
M5005_Spy1468	M5005_Spy1468	tRNA (m(7)G46) methyltransferase	-0.667430273	4.42272E-07
M5005_Spy1475	M5005_Spy1475	acetyltransferase	-0.242288441	0.080203529
M5005_Spy1476	M5005_Spy1476	ATP/GTP hydrolase	-1.32669054	0.077911829
M5005_Spy1513	M5005_Spy1513	aspartate aminotransferase	-0.347847113	4.59772E-08
M5005_Spy1519	recG	ATP-dependent DNA helicase	-1.477959971	2.6649E-47
M5005_Spy1543	scrB	sucrose-6-phosphate hydrolase	0.773015774	3.91666E-06
M5005_Spy1544	scrR	sucrose operon repressor	0.735678937	3.55115E-25
M5005_Spy1560	M5005_Spy1560	phosphatidylglycerophosphatase B	-0.346662463	9.88302E-05
M5005_Spy1566	recD	exodeoxyribonuclease V alpha chain	-0.215257528	0.006097375
M5005_Spy1571	M5005_Spy1571	c3-degrading proteinase	0.215180204	0.001649004
M5005_Spy1586	nupC	nucleoside permease	0.161251567	0.029871029
M5005_Spy1589	crgR	transcriptional regulator, GntR family	-0.283055669	0.038801584
M5005_Spy1611	rpoE	DNA-directed RNA polymerase delta chain	0.444206192	1.56602E-08
M5005_Spy1617	M5005_Spy1617	tRNA pseudouridine synthase A	-1.410575605	0.000199844
M5005_Spy1639	lacR2	lactose phosphotransferase system repressor	0.313642889	1.95803E-07
M5005_Spy1682	msmK	multiple sugar transport ATP-binding protein	-0.200717373	0.056813636
M5005_Spy1683	lrp	leucine rich protein	-0.24189316	0.061166736
M5005_Spy1687	sclA	collagen-like surface protein A	-0.313629291	0.045201594
M5005_Spy1719	emm1.0	M protein	0.280897974	0.008249929
M5005_Spy1720	mga	trans-acting positive regulator	0.457217364	8.57288E-16
M5005_Spy1726	M5005_Spy1726	ABC transporter permease protein	0.179580586	0.039698971
M5005_Spy1727	M5005_Spy1727	ABC transporter ATP-binding protein	0.304511721	0.000305083
M5005_Spy1728	M5005_Spy1728	periplasmic component of efflux system	0.28159381	2.38165E-05
M5005_Spy1735	speB	streptococcal pyrogenic exotoxin B	0.296156622	9.54081E-08
M5005_Spy1736	M5005_Spy1736	hypothetical protein	0.51525382	0.007265281
M5005_Spy1737	rgg	transcriptional regulator	0.249339696	0.001945019
M5005_Spy1744	M5005_Spy1744	PTS system, cellobiose-specific IIC component	0.16668779	0.053747552
M5005_Spy1753	pbp2A	multimodular transpeptidase-transglycosylase	-1.555403882	3.24363E-59
M5005_Spy1754	M5005_Spy1754	translation initiation inhibitor	0.358163951	8.76587E-05
M5005_Spy1770	hutI	imidazolonepropionase	-0.349788278	0.000349395
M5005_Spy1779	M5005_Spy1779	transcriptional regulator, LuxR family	0.147536125	0.099312082
M5005_Spy1784	M5005_Spy1784	PTS system, trehalose-specific IIBC component	0.165139415	0.0738986
M5005_Spy1787	M5005_Spy1787	glyoxalase family protein	0.167518665	0.043153018
M5005_Spy1790	M5005_Spy1790	acetyltransferase	0.350863396	0.03960479
M5005_Spy1794	M5005_Spy1794	putative membrane spanning protein	-0.465929667	3.56245E-07
M5005_Spy1802	ruvA	holliday junction DNA helicase	-1.731745336	0.086681958
M5005_Spy1809	uviB	bacteriocin	0.190499834	0.08100213
M5005_Spy1818	cadC	cadmium efflux system accessory protein	0.283196614	0.010438289
M5005_Spy1828	M5005_Spy1828	phage infection protein	0.382742226	1.91016E-06
M5005_Spy1830	M5005_Spy1830	transcriptional regulator, TetR family	0.221277378	0.002013881
M5005_Spy1837	M5005_Spy1837	phosphoesterase, DHH family protein	-1.905262228	1.77982E-21
M5005_Spy1849	M5005_Spy1849	zinc protease	0.311997109	0.001023002
M5005_Spy1850	M5005_Spy1850	zinc protease	0.280126642	0.002013881
M5005_Spy1851	hasA	hyaluronan synthase	0.146594367	0.099575179
M5005_Spy1866	parB	chromosome partitioning protein	-0.264045187	0.003194235

* fitness score is defined as log2 fold changes in sequence read counts between input and output pools. Only genes with statistically significant change (q value < 0.1) are listed.
