# Supplementary material for: Novel Genes Required for the Fitness of Streptococcus pyogenes in Human Saliva
Source: mSphere. 2017 Nov 1;2(6):e00460-17. doi: 10.1128/mSphereDirect.00460-17 (PMC5663985; doi:10.1128/mSphereDirect.00460-17)
Supplement: TABLE S5 [file sph006172393st6.pdf]

TABLE S5 Fitness score of each gene in the M1 GAS genome after 24 hour saliva incubation.

locus_tag	gene_name	function	Fitness score*	q.value
M5005_Spy0004	M5005_Spy0004	GTP-binding protein	-0.665004783	0.010657001
M5005_Spy0013	ftsH	cell division protein	-0.540255124	0.012515326
M5005_Spy0014	M5005_Spy0014	amino acid permease	-0.373112573	1.99782E-12
M5005_Spy0017	sibA	secreted protein	-4.058691305	0.000996406
M5005_Spy0022	M5005_Spy0022	phosphoribosylaminoimidazole-succinocarboxamide synthase	0.244397101	0.004571132
M5005_Spy0024	purF	amidophosphoribosyltransferase	0.628808878	2.94011E-28
M5005_Spy0025	purM	phosphoribosylformylglycinamidine cyclo-ligase	0.315246875	6.01592E-06
M5005_Spy0029	purD	phosphoribosylamine--glycine ligase	0.15681278	0.057010926
M5005_Spy0031	purK	phosphoribosylaminoimidazole carboxylase ATPase subunit	0.184328333	0.070302886
M5005_Spy0033	purB	adenylosuccinate lyase	0.245133331	0.002537863
M5005_Spy0036	M5005_Spy0036	protein tyrosine phosphatase	1.156193535	0.041533509
M5005_Spy0040	adhA	alcohol dehydrogenase	0.315290017	0.00226187
M5005_Spy0041	M5005_Spy0041	Na+ driven multidrug efflux pump	0.185403687	0.006648581
M5005_Spy0075	M5005_Spy0075	4-diphosphocytidyl-2-C-methyl-D-erythritol kinase	0.307908538	0.000726122
M5005_Spy0077	adcR	transcriptional regulator, MarR family	0.493200724	0.006974333
M5005_Spy0084	rpoC	DNA-directed RNA polymerase beta' chain	0.487830148	0.029023215
M5005_Spy0086	comYA	comG operon protein 1	-0.135116642	0.059947646
M5005_Spy0093	M5005_Spy0093	adenine-specific methyltransferase	0.16477377	0.009073766
M5005_Spy0094	ackA	acetate kinase	-2.887494997	0.053003495
M5005_Spy0096	proC	pyrroline-5-carboxylate reductase	0.153119358	0.092715344
M5005_Spy0097	pepA	glutamyl aminopeptidase	0.156641254	0.028037604
M5005_Spy0101	M5005_Spy0101	tRNA binding domain protein	0.205864916	0.008029168
M5005_Spy0103	M5005_Spy0103	deoxyadenosine kinase/deoxyguanosine kinase	0.336216853	0.001389716
M5005_Spy0106	rofA	transcriptional regulator	-0.183928077	0.003940136
M5005_Spy0107	M5005_Spy0107	fibronectin-binding protein	0.186548615	0.005085303
M5005_Spy0110	eftLSLB	putative exported protein	-0.254260236	0.004130506
M5005_Spy0112	M5005_Spy0112	transposase	0.158869452	0.006853968
M5005_Spy0114	M5005_Spy0114	sortase	0.133647982	0.081416785
M5005_Spy0116	atoE	short-chain fatty acids transporter	0.163778128	0.015300755
M5005_Spy0117	M5005_Spy0117	transcriptional regulator, LysR family	0.143517086	0.021644626
M5005_Spy0119	M5005_Spy0119	acetyl-CoA acetyltransferase	0.302136411	5.58187E-06
M5005_Spy0120	atoD2	acetate CoA-transferase alpha subunit	0.253294588	0.012515326
M5005_Spy0121	M5005_Spy0121	acetyl-CoA:acetoacetyl-CoA transferase beta subunit	0.376502453	0.000945219
M5005_Spy0122	M5005_Spy0122	putative DNA-binding protein	-0.351892926	0.000946492
M5005_Spy0123	M5005_Spy0123	translation initiation inhibitor	0.42702981	0.030011302
M5005_Spy0124	sloR	transcriptional regulator	0.242733466	0.000420179
M5005_Spy0127	ntpK	V-type sodium ATP synthase subunit K	0.327164728	0.012515326
M5005_Spy0128	ntpE	V-type sodium ATP synthase subunit E	-0.122698651	0.079707588
M5005_Spy0132	ntpB	V-type sodium ATP synthase subunit B	0.225740798	0.003190623
M5005_Spy0143	M5005_Spy0143	hypothetical protein	0.461319239	0.0257664
M5005_Spy0144	M5005_Spy0144	hypothetical protein	-0.341250295	0.073122383
M5005_Spy0148	M5005_Spy0148	PTS system, 3-keto-L-gulonate specific IIC component	0.196173265	0.033706494
M5005_Spy0151	M5005_Spy0151	3-keto-L-gulonate-6-phosphate decarboxylase	-0.217634407	0.039844253
M5005_Spy0153	araD	L-ribulose-5-phosphate 4-epimerase	0.448641474	0.00226187
M5005_Spy0158	opuABC	glycine betaine-binding protein/glycine betaine transport system permease protein	0.24073878	0.013081521
M5005_Spy0161	perR	ferric uptake regulation protein	0.358291756	0.016923119
M5005_Spy0164	M5005_Spy0164	parB-like nuclease	-0.236067844	0.003656682
M5005_Spy0166	M5005_Spy0166	transposase	-0.213852034	0.098801441
M5005_Spy0168	M5005_Spy0168	transposase	-0.36264416	0.002961694
M5005_Spy0169	M5005_Spy0169	malonate permease	0.254525406	0.00011877
M5005_Spy0170	nadC	nicotinate-nucleotide pyrophosphorylase (carboxylating)	0.155264141	0.068987033
M5005_Spy0175	tgt	queuine tRNA-ribosyltransferase	0.188055286	0.051073256
M5005_Spy0178	M5005_Spy0178	metal-dependent hydrolase	0.239175921	0.01251374
M5005_Spy0180	M5005_Spy0180	S-layer homology domain	0.143875572	0.053003495
M5005_Spy0182	speG	exotoxin type G precursor	-0.207507899	0.020074681
M5005_Spy0183	M5005_Spy0183	putative membrane associated protein	0.288963975	0.030627964
M5005_Spy0196	M5005_Spy0196	multidrug resistance ABC transporter ATP-binding and permease protein	0.731356011	7.21887E-21
M5005_Spy0197	M5005_Spy0197	multidrug resistance ABC transporter ATP-binding and permease protein	0.586289653	4.70347E-08
M5005_Spy0201	M5005_Spy0201	carbonic anhydrase	0.604146494	0.000777963
M5005_Spy0208	M5005_Spy0208	60 kDa inner membrane protein YIDC	0.378482402	0.003755128
M5005_Spy0213	M5005_Spy0213	N-acetylneuraminate-binding protein	0.247023752	0.007658404
M5005_Spy0217	nanH	N-acetylneuraminate lyase	0.379657729	0.000337974
M5005_Spy0218	M5005_Spy0218	N-acetylmannosamine kinase	0.45662322	0.003280108
M5005_Spy0224	rpe	ribulose-phosphate 3-epimerase	-1.297249405	7.60141E-06
M5005_Spy0225	M5005_Spy0225	thiamin pyrophosphokinase	-1.155154563	9.11318E-08
M5005_Spy0229	prgA	surface exclusion protein	0.409144878	9.0459E-08
M5005_Spy0237	M5005_Spy0237	putative membrane spanning protein	0.14223694	0.017600519
M5005_Spy0238	bacA	putative undecaprenol kinase	0.374255847	0.000436339
M5005_Spy0240	M5005_Spy0240	undecaprenyl-phosphate alpha-N-acetylglucosaminephosphotransferase	-0.933389105	1.471E-11
M5005_Spy0241	rgpG	putative cytosolic protein	-0.479394132	0.001048597
M5005_Spy0247	M5005_Spy0247	D-alanyl-D-alanine carboxypeptidase	0.549705123	5.26841E-16
M5005_Spy0248	dacA2	D-alanyl-D-alanine carboxypeptidase	-0.235561863	0.001549324
M5005_Spy0249	oppA	oligopeptide-binding protein	0.58721773	0.012623158
M5005_Spy0250	oppB	oligopeptide transport system permease protein	0.583755139	0.000420179
M5005_Spy0251	oppC	oligopeptide transport system permease protein	0.540206486	0.044729181
M5005_Spy0252	oppD	oligopeptide transport ATP-binding protein	0.699645769	0.000160727
M5005_Spy0253	oppF	oligopeptide transport ATP-binding protein	0.445960022	0.035734291
M5005_Spy0266	M5005_Spy0266	methyltransferase	-0.193157971	0.015461712
M5005_Spy0267	M5005_Spy0267	putative cytosolic protein	0.239579573	0.000479484
M5005_Spy0268	M5005_Spy0268	hypothetical protein	-0.330037686	0.015976035

M5005_Spy0271	M5005_Spy0271	ABC transporter substrate-binding protein	0.398367517	3.80984E-13
M5005_Spy0272	M5005_Spy0272	ABC transporter ATP-binding protein	0.341832509	6.20946E-09
M5005_Spy0273	M5005_Spy0273	ABC transporter permease protein	0.307664351	1.4867E-06
M5005_Spy0274	braB	branched-chain amino acid transport system carrier protein	0.284865561	4.57064E-07
M5005_Spy0278	gidB	glucose inhibited division protein B	-0.253396474	0.055279479
M5005_Spy0279	lemA	LemA	0.650278118	0.053003495
M5005_Spy0280	M5005_Spy0280	heat shock protein	0.307480851	0.059241498
M5005_Spy0288	snf	SWF/SNF family helicase	-0.385826857	1.37911E-07
M5005_Spy0292	M5005_Spy0292	aminodeoxychorismate lyase family	-2.934037901	0.026263958
M5005_Spy0300	M5005_Spy0300	hydrolase, HAD superfamily	0.563197772	0.007630395
M5005_Spy0306	M5005_Spy0306	CBS domain containing protein	-0.40801628	0.07730174
M5005_Spy0308	M5005_Spy0308	putative cytosolic protein	-0.37270861	0.045937634
M5005_Spy0312	M5005_Spy0312	23S rRNA methyltransferase	0.187842942	0.073238915
M5005_Spy0313	M5005_Spy0313	riboflavin transporter	-1.078108144	0.00396604
M5005_Spy0321	fhuG	ferrichrome transport system permease protein	0.139219058	0.053029593
M5005_Spy0322	fhuB	ferrichrome transport system permease protein	0.225532045	0.009103104
M5005_Spy0326	M5005_Spy0326	export protein for polysaccharides and teichoic acids	-0.599705726	0.001387193
M5005_Spy0327	upp	uracil phosphoribosyltransferase	-0.801230988	1.329E-07
M5005_Spy0330	tmk	thymidylate kinase	-6.322129727	0.000518282
M5005_Spy0335	M5005_Spy0335	corrin/porphyrin methyltransferase	0.315595694	0.080103876
M5005_Spy0336	M5005_Spy0336	putative membrane associated protein	-0.161633945	0.076731256
M5005_Spy0339	exoA	exodeoxyribonuclease III	-0.352901389	0.015461712
M5005_Spy0340	lctO	L-lactate oxidase	-0.176318206	0.03022142
M5005_Spy0349	nrdE1	ribonucleoside-diphosphate reductase alpha chain	0.157979372	0.021035449
M5005_Spy0350	M5005_Spy0350	hypothetical protein	-0.523338202	0.019221914
M5005_Spy0354	M5005_Spy0354	hypothetical protein	-0.243266706	0.018478687
M5005_Spy0355	M5005_Spy0355	hypothetical protein	-0.341615166	0.025342832
M5005_Spy0356	speJ	exotoxin type J precursor	-0.326584202	0.003845103
M5005_Spy0357	M5005_Spy0357	hypothetical protein	-0.205475642	0.067724702
M5005_Spy0367	scaR	iron-dependent repressor	-0.372659988	0.008985218
M5005_Spy0373	M5005_Spy0373	integral membrane protein	-1.168078319	0.006902019
M5005_Spy0374	rplK	LSU ribosomal protein L11P	4.968094849	0.060683246
M5005_Spy0382	msrA2	peptide methionine sulfoxide reductase	-0.761459896	5.49792E-13
M5005_Spy0383	M5005_Spy0383	D-alanyl-D-alanine carboxypeptidase	-0.219921483	0.053029593
M5005_Spy0386	phoH	phoH protein	0.132041334	0.092715344
M5005_Spy0392	M5005_Spy0392	hypothetical protein	-0.265215692	0.084768133
M5005_Spy0401	M5005_Spy0401	putative cytosolic protein	-0.599019706	0.022101334
M5005_Spy0404	M5005_Spy0404	hypothetical protein	0.216724338	0.080364615
M5005_Spy0408	fpg	formamidopyrimidine-DNA glycosylase	0.494796502	0.001830271
M5005_Spy0410	M5005_Spy0410	ATPase	0.243982751	0.000702035
M5005_Spy0411	M5005_Spy0411	multidrug resistance protein B	0.210506606	0.010945467
M5005_Spy0414	M5005_Spy0414	exoribonuclease II	0.274822071	0.004737113
M5005_Spy0418	M5005_Spy0418	permease	0.728287151	0.028561733
M5005_Spy0420	M5005_Spy0420	glucosyltransferase	-0.322616694	0.000965194
M5005_Spy0421	gloA	lactoylglutathione lyase	-0.25417388	0.055886711
M5005_Spy0427	thrS	threonyl-tRNA synthetase	0.730878554	0.010952329
M5005_Spy0430	M5005_Spy0430	ABC transporter permease protein	0.415981979	0.000502737
M5005_Spy0431	M5005_Spy0431	dihydroxyacetone kinase	-0.365752544	0.092922588
M5005_Spy0432	M5005_Spy0432	acetyl-CoA acetyltransferase	0.361952329	0.007609484
M5005_Spy0433	M5005_Spy0433	long-chain-fatty-acid--CoA ligase	0.196125091	0.045247482
M5005_Spy0436	vicK	two-component sensor histidine kinase	-0.752220911	3.56529E-06
M5005_Spy0439	smc	chromosome partition protein	-0.169939454	0.006974333
M5005_Spy0442	M5005_Spy0442	putative cytosolic protein	-0.2822698	0.03026461
M5005_Spy0447	M5005_Spy0447	glycosyltransferase involved in cell wall biogenesis	-0.15902536	0.080103876
M5005_Spy0460	M5005_Spy0460	hypothetical protein	0.229072648	0.048354292
M5005_Spy0462	M5005_Spy0462	hypothetical protein	-0.269348546	0.009526976
M5005_Spy0463	M5005_Spy0463	putative cytosolic protein	-0.229154019	0.038014411
M5005_Spy0466	M5005_Spy0466	hypothetical protein	-0.418950118	5.16552E-06
M5005_Spy0471	M5005_Spy0471	hydrolase, HAD superfamily	0.191271445	0.047828105
M5005_Spy0472	ftsY	cell division protein	-0.369328675	0.009752698
M5005_Spy0478	M5005_Spy0478	putative membrane spanning protein	-0.18644643	0.012654748
M5005_Spy0480	M5005_Spy0480	transcription accessory protein	0.170608423	0.022216871
M5005_Spy0483	M5005_Spy0483	stress-responsive transcriptional regulator	1.767840586	0.032316602
M5005_Spy0485	lgt	prolipoprotein diacylglyceryl transferase	-0.333569372	0.049555241
M5005_Spy0486	M5005_Spy0486	hypothetical protein	-0.688506625	7.42415E-25
M5005_Spy0491	M5005_Spy0491	peptidase family U32	0.204144541	0.002362146
M5005_Spy0498	M5005_Spy0498	transcriptional regulator	0.250906561	0.030466932
M5005_Spy0513	M5005_Spy0513	putative cytosolic protein	-0.481820775	1.26156E-09
M5005_Spy0517	regR	transcriptional regulator, LacI family	-0.313439269	6.30121E-06
M5005_Spy0532	ftsX	cell division protein	-1.810786977	0.03511752
M5005_Spy0536	dinG	ATP-dependent helicase	0.345617357	2.82347E-12
M5005_Spy0539	M5005_Spy0539	ATP-binding protein	0.692569383	0.00966813
M5005_Spy0542	pepD	dipeptidase	0.123574802	0.078176188
M5005_Spy0545	agaS	galactosamine-6-phosphate deaminase (isomerizing)	0.198373773	0.003561777
M5005_Spy0553	gyrB	DNA gyrase subunit B	5.490126719	0.03292876
M5005_Spy0554	M5005_Spy0554	septation ring formation regulator	0.917687423	0.089936639
M5005_Spy0560	M5005_Spy0560	transcriptional regulator	-0.168989421	0.013959393
M5005_Spy0563	sagB	streptolysin S biosynthesis protein	-0.233676538	0.001549324
M5005_Spy0567	sagF	streptolysin S biosynthesis protein	-0.292561784	0.00022828
M5005_Spy0568	sagG	streptolysin S export ATP-binding protein	-2.996793078	2.80862E-12
M5005_Spy0569	sagH	streptolysin S export transmembrane protein	-3.755023247	2.38518E-19
M5005_Spy0570	sagI	streptolysin S export transmembrane protein	-4.838027541	3.35922E-06
M5005_Spy0573	lig	NAD-dependent DNA ligase	-0.962227389	0.063056115

M5005_Spy0591	M5005_Spy0591	ABC transporter permease protein	-0.271919708	0.000118696
M5005_Spy0592	M5005_Spy0592	ABC transporter ATP-binding protein	0.223107764	0.080103876
M5005_Spy0608	rgpFc	alpha-L-Rha alpha-1,2-L-rhamnosyltransferase/alpha-L-Rha alpha-1,3-L-rhamnosyltransferase	0.76685673	0.050214614
M5005_Spy0609	M5005_Spy0609	phosphoglycerol transferase	-0.330537792	2.92346E-08
M5005_Spy0610	M5005_Spy0610	glycosyltransferase	-0.260047575	0.001332251
M5005_Spy0611	M5005_Spy0611	hypothetical protein	-0.413316355	0.000118696
M5005_Spy0623	M5005_Spy0623	methyltransferase	-0.270538786	0.057240245
M5005_Spy0624	aroD	3-dehydroquinate dehydratase	-0.790123126	0.069301282
M5005_Spy0625	aroF	chorismate synthase	0.164763093	0.06552743
M5005_Spy0628	folC2	folylpolyglutamate synthase/dihydrofolate synthase	0.302915937	0.004863907
M5005_Spy0630	nifS1	cysteine desulfhydrase	-0.785746308	8.07078E-16
M5005_Spy0631	thiI	thiamine biosynthesis protein	-0.891808276	5.69544E-27
M5005_Spy0632	capA	capsule biosynthesis protein	0.248789323	0.00199065
M5005_Spy0639	pyrR	pyrR bifunctional protein	0.288980515	0.080364615
M5005_Spy0641	pyrB	aspartate carbamoyltransferase	-0.34417952	3.8062E-06
M5005_Spy0642	carA	carbamoyl-phosphate synthase small chain	-0.482512662	9.85233E-10
M5005_Spy0643	carB	carbamoyl-phosphate synthase large chain	-0.504546485	3.92332E-27
M5005_Spy0644	M5005_Spy0644	periplasmic component of efflux system	-1.199493265	2.2698E-119
M5005_Spy0645	M5005_Spy0645	ABC transporter ATP-binding protein	-1.003725727	5.66253E-50
M5005_Spy0646	M5005_Spy0646	ABC transporter permease protein	-1.080793964	1.13135E-58
M5005_Spy0650	M5005_Spy0650	hypothetical protein	-0.568062657	0.019077018
M5005_Spy0651	M5005_Spy0651	cell surface protein	-0.146485771	0.018288297
M5005_Spy0654	M5005_Spy0654	transcriptional regulator, TetR family	-0.158974186	0.081707288
M5005_Spy0658	M5005_Spy0658	regulatory protein	0.250587274	0.003835445
M5005_Spy0659	apbA	2-dehydropantoate 2-reductase	-0.200916289	0.054447704
M5005_Spy0660	fruR	fructose repressor	-0.348845785	0.0019036
M5005_Spy0663	mur11	autolysin	-0.294166996	0.015606729
M5005_Spy0664	mur12	autolysin	-0.179426866	0.00226187
M5005_Spy0665	M5005_Spy0665	transposase	-0.54208821	9.04652E-05
M5005_Spy0667	M5005_Spy0667	exotoxin type C precursor	-0.317119474	0.03787804
M5005_Spy0675	M5005_Spy0675	hypothetical protein	-0.2935379	0.054896731
M5005_Spy0678	M5005_Spy0678	5'-nucleotidase	-0.126810121	0.076743411
M5005_Spy0681	M5005_Spy0681	two-component system histidine kinase	0.287256033	5.34132E-06
M5005_Spy0693	M5005_Spy0693	hypothetical protein	-0.278946483	4.92321E-08
M5005_Spy0697	arsC	arsenate reductase family protein	-0.886984646	0.015161945
M5005_Spy0700	cpsX	attenuator of transcription, LytR family regulator	0.19235965	0.080103876
M5005_Spy0701	cpsY	transcriptional regulator, LysR family	0.632360713	1.37813E-09
M5005_Spy0703	pyrF	orotidine 5'-phosphate decarboxylase	0.223381483	0.012394136
M5005_Spy0716	M5005_Spy0716	hypothetical protein	-0.249001769	0.054833279
M5005_Spy0719	M5005_Spy0719	glutathione S-transferase	0.189793287	0.02035695
M5005_Spy0720	M5005_Spy0720	putative exfoliative toxin	0.231721192	0.00226187
M5005_Spy0721	M5005_Spy0721	hypothetical protein	-0.490745007	0.00838298
M5005_Spy0722	miaA	tRNA delta(2)-isopentenylpyrophosphate transferase	-0.558768581	8.84514E-07
M5005_Spy0728	apt	adenine phosphoribosyltransferase	1.254715727	7.31515E-08
M5005_Spy0730	nth	endonuclease III	0.456694225	0.009967812
M5005_Spy0732	M5005_Spy0732	NIF3-related protein	0.300362579	0.064528984
M5005_Spy0736	cpsFQ	dTDP-glucose 4,6-dehydratase	-0.882496835	0.093633876
M5005_Spy0742	M5005_Spy0742	hypothetical protein	-0.315158479	0.067323966
M5005_Spy0743	M5005_Spy0743	ABC transporter substrate-binding protein	0.184722158	0.039844253
M5005_Spy0745	M5005_Spy0745	ABC transporter permease protein	0.222893679	0.073831208
M5005_Spy0749	M5005_Spy0749	hypothetical protein	-0.281725548	0.019110027
M5005_Spy0751	acoA	pyruvate dehydrogenase E1 component alpha subunit	1.346770979	8.82618E-12
M5005_Spy0752	acoB	pyruvate dehydrogenase E1 component beta subunit	1.30046369	2.18003E-05
M5005_Spy0763	femD	phosphoglucosamine mutase	0.434777412	0.035989381
M5005_Spy0767	M5005_Spy0767	4-nitrophenylphosphatase	0.311027581	0.000362317
M5005_Spy0772	M5005_Spy0772	hypothetical protein	-0.199714385	0.097893876
M5005_Spy0776	lepA	GTP-binding protein	0.218927122	0.059896994
M5005_Spy0779	M5005_Spy0779	putative membrane spanning protein	-0.116513698	0.051658108
M5005_Spy0780	M5005_Spy0780	PTS system, mannose/fructose family IIA component	0.365181175	0.012493001
M5005_Spy0781	ptsB	PTS system, mannose/fructose family IIB component	0.238802692	0.038826054
M5005_Spy0783	ptsD	PTS system, mannose/fructose family IID component	0.318703918	0.004175881
M5005_Spy0784	M5005_Spy0784	two-component sensor kinase	-0.165988277	0.010618701
M5005_Spy0785	M5005_Spy0785	two-component response regulator	0.176926985	0.089936639
M5005_Spy0790	gabD	succinate-semialdehyde dehydrogenase [NADP+]	-0.277852605	0.009302731
M5005_Spy0791	uvrC	excinuclease ABC subunit C	-0.490818006	9.94957E-15
M5005_Spy0792	M5005_Spy0792	NAD(P)H-dependent quinone reductase	-4.591449118	0.000403352
M5005_Spy0793	M5005_Spy0793	Xaa-His dipeptidase	0.49924245	1.44978E-05
M5005_Spy0797	M5005_Spy0797	hypothetical protein	-0.472591467	0.038826054
M5005_Spy0803	srtI	protein involved in lantibiotic production	-0.113585198	0.062580786
M5005_Spy0804	srtR	nisin biosynthesis two-component response regulator	-0.364818885	7.43452E-07
M5005_Spy0805	srtK	nisin biosynthesis sensor protein	-0.409132164	1.19398E-05
M5005_Spy0806	srtA	lantibiotic precursor	-0.30336635	0.005448808
M5005_Spy0807	srtT	ABC transporter (ATP binding)-lantibiotic associated	-0.172830249	0.028614514
M5005_Spy0808	srtF	lantibiotic transport ATP-binding protein	-0.147961448	0.034461155
M5005_Spy0809	srtE	lantibiotic transport permease protein	-0.195167556	0.035288119
M5005_Spy0810	srtG	lantibiotic transport permease protein	-0.419005932	0.000241508
M5005_Spy0817	dacA1	D-alanyl-D-alanine carboxypeptidase	-0.287211373	0.001332251
M5005_Spy0820	folC1	folylpolyglutamate synthase/dihydrofolate synthase	-4.717898063	0.003090914
M5005_Spy0829	M5005_Spy0829	spermidine/putrescine-binding protein	-0.477750882	0.053029593
M5005_Spy0830	dpiA	transcriptional regulatory protein	-0.165461731	0.023340543
M5005_Spy0832	malP	malate-sodium symport	0.129759918	0.047608087
M5005_Spy0835	M5005_Spy0835	class B acid phosphatase	-0.149166912	0.02332191
M5005_Spy0838	M5005_Spy0838	lipase/acylhydrolase family protein	-0.251130081	0.001983269

M5005_Spy0840	radC	DNA repair protein	-0.156673248	0.031269306
M5005_Spy0841	M5005_Spy0841	glutamine amidotransferase, class I	-0.317951282	0.028997701
M5005_Spy0847	M5005_Spy0847	GTP pyrophosphokinase	-0.638723899	0.03903346
M5005_Spy0852	M5005_Spy0852	short chain dehydrogenase	-0.30914668	0.008138086
M5005_Spy0858	xpt	xanthine phosphoribosyltransferase	-0.201371271	0.012493001
M5005_Spy0860	apbE	thiamine biosynthesis lipoprotein	-1.001686198	0.023885916
M5005_Spy0872	nox	NADH oxidase H2O-forming	0.174444631	0.015461712
M5005_Spy0874	gyrA	DNA gyrase subunit A	-0.987238492	0.021907198
M5005_Spy0876	M5005_Spy0876	lactoylglutathione lyase	-0.248641795	0.00183013
M5005_Spy0878	M5005_Spy0878	hypothetical protein	-0.191887855	0.045937634
M5005_Spy0884	smf	Smf	-0.161746767	0.043837925
M5005_Spy0886	M5005_Spy0886	transcriptional regulator, LysR family	-0.156037626	0.024270271
M5005_Spy0887	M5005_Spy0887	transcriptional regulator, LysR family	-0.344860984	0.000273569
M5005_Spy0892	satE	SatE	0.158971492	0.073831208
M5005_Spy0893	gid	glucose inhibited division protein A	0.191298547	0.023340543
M5005_Spy0894	oadA2	oxaloacetate decarboxylase alpha chain	0.136770458	0.068694134
M5005_Spy0895	M5005_Spy0895	putative membrane associated protein	-0.267701647	0.021926363
M5005_Spy0899	citG	transcriptional regulator, GntR family	-0.155940452	0.016254912
M5005_Spy0900	M5005_Spy0900	Mg2+/citrate complex secondary transporter	-0.112199025	0.086037306
M5005_Spy0902	M5005_Spy0902	biotin carboxyl carrier protein of oxaloacetate decarboxylase	0.204535818	0.065627356
M5005_Spy0903	oadB	oxaloacetate decarboxylase beta chain	0.306661003	0.001004456
M5005_Spy0906	citE	citrate lyase beta chain/citryl-CoA lyase subunit	0.152234138	0.021283451
M5005_Spy0907	citF	citrate lyase alpha chain/citrate CoA-transferase	0.291146128	2.75204E-06
M5005_Spy0908	citX	apo-citrate lyase phosphoribosyl-dephospho-CoA transferase	0.144659069	0.034413833
M5005_Spy0909	oadA1	oxaloacetate decarboxylase alpha chain	0.169435659	0.006628769
M5005_Spy0911	M5005_Spy0911	hypothetical protein	-0.141584281	0.078601573
M5005_Spy0914	M5005_Spy0914	phage transcriptional repressor	-0.177700247	0.009103104
M5005_Spy0918	M5005_Spy0918	hypothetical protein	-0.243234013	0.000729209
M5005_Spy0921	M5005_Spy0921	ABC transporter ATP-binding protein	-0.121444385	0.056554657
M5005_Spy0923	M5005_Spy0923	pyridoxine kinase	-0.216548825	0.001150693
M5005_Spy0925	rnhB	anaerobic ribonucleoside-triphosphate reductase	-0.354341446	0.003599192
M5005_Spy0926	M5005_Spy0926	cardiolipin synthetase	-2.584797561	4.41145E-12
M5005_Spy0929	M5005_Spy0929	SIR2 family protein	0.1953777	0.029218989
M5005_Spy0931	M5005_Spy0931	glycine cleavage system H protein	0.300217169	0.069753844
M5005_Spy0934	M5005_Spy0934	lipoate-protein ligase A	0.205622966	0.012216566
M5005_Spy0937	M5005_Spy0937	transporter	-1.570665731	0.001714623
M5005_Spy0938	pgmA	phosphoglucomutase/phosphomannomutase	-0.495373701	0.005765678
M5005_Spy0939	M5005_Spy0939	nucleoside transport system permease protein	0.762944266	1.16149E-24
M5005_Spy0940	M5005_Spy0940	nucleoside transport system permease protein	0.808308031	5.71726E-26
M5005_Spy0941	M5005_Spy0941	nucleoside transport ATP-binding protein	0.740973987	5.71726E-26
M5005_Spy0942	M5005_Spy0942	nucleoside-binding protein	0.517779639	1.17283E-11
M5005_Spy0943	cdd	cytidine deaminase	0.606962135	9.0459E-08
M5005_Spy0944	M5005_Spy0944	16S rRNA m(2)G 1207 methyltransferase	-0.46763997	3.28147E-13
M5005_Spy0947	ciaH	sensor protein	-0.289342227	0.000723319
M5005_Spy0949	pepN	lysyl aminopeptidase/alanine aminopeptidase	0.189947281	0.006829623
M5005_Spy0951	pstB	phosphate transport ATP-binding protein	-1.599727177	7.7655E-100
M5005_Spy0952	pstB2	phosphate transport ATP-binding protein	-1.85889123	5.07094E-53
M5005_Spy0953	pstA	phosphate transport system permease protein	-1.893478286	1.7075E-162
M5005_Spy0954	pstC	phosphate transport system permease protein	-1.934505396	1.37154E-65
M5005_Spy0955	pstS	phosphate-binding protein	-2.028958081	2.6174E-112
M5005_Spy0961	truB	tRNA pseudouridine synthase B	-1.471604692	0.003736548
M5005_Spy0970	M5005_Spy0970	NAD-dependent K+ or Na+ uptake system component	-1.421419043	0.002161896
M5005_Spy0971	M5005_Spy0971	general stress protein, Gls24 family	-0.729582942	9.50436E-05
M5005_Spy0972	M5005_Spy0972	hypothetical protein	-0.74247025	0.051969253
M5005_Spy0973	M5005_Spy0973	general stress protein, Gls24 family	-0.796444899	1.99619E-07
M5005_Spy0975	M5005_Spy0975	hypothetical protein	-0.929213758	4.86128E-06
M5005_Spy0980	M5005_Spy0980	cobalt-zinc-cadmium resistance protein	-0.213823413	0.038826054
M5005_Spy0981	cfa	cAMP factor	-0.248755992	0.000414249
M5005_Spy0982	M5005_Spy0982	histidine-binding protein	-0.186238825	0.023885916
M5005_Spy0986	glmS	glucosamine--fructose-6-phosphate aminotransferase (isomerizing)	5.006626723	0.058546555
M5005_Spy0987	sipC	signal peptidase I	-0.739674013	1.81062E-27
M5005_Spy0992	M5005_Spy0992	ABC transporter ATP-binding protein	-1.0011065	4.48554E-11
M5005_Spy0993	M5005_Spy0993	ABC transporter permease protein	-1.100487928	1.66809E-09
M5005_Spy0995	M5005_Spy0995	phage protein	-0.369379627	0.023340543
M5005_Spy0999	M5005_Spy0999	phage protein	0.192884927	0.020757093
M5005_Spy1000	M5005_Spy1000	phage protein	-0.220096914	0.001418424
M5005_Spy1001	M5005_Spy1001	phage-associated cell wall hydrolase	0.290821588	0.006171991
M5005_Spy1003	M5005_Spy1003	phage protein	-0.388171259	8.61926E-05
M5005_Spy1006	M5005_Spy1006	phage structural protein	-0.153974239	0.027782755
M5005_Spy1021	M5005_Spy1021	phage protein	-0.254826936	0.0001313
M5005_Spy1022	M5005_Spy1022	portal protein	-0.163801909	0.021926363
M5005_Spy1023	M5005_Spy1023	terminase large subunit	-0.241228951	0.000337722
M5005_Spy1044	M5005_Spy1044	phage protein	-0.352452909	0.011597364
M5005_Spy1046	M5005_Spy1046	phage protein	-0.184063525	0.093573571
M5005_Spy1050	M5005_Spy1050	phage transcriptional repressor	-0.871526387	0.001036328
M5005_Spy1051	M5005_Spy1051	phage protein	-0.200203681	0.019927209
M5005_Spy1055	glgP	glycogen phosphorylase	-0.390985413	3.2408E-15
M5005_Spy1056	malM	4-alpha-glucanotransferase	0.546280281	1.79676E-29
M5005_Spy1057	malR	transcriptional regulator, LacI family	0.470840295	8.3561E-12
M5005_Spy1058	malE	maltose/maltodextrin-binding protein	0.125967292	0.03903346
M5005_Spy1061	M5005_Spy1061	transcriptional regulator, LacI family	0.433144416	1.47207E-16
M5005_Spy1063	malD	maltodextrin transport system permease protein	0.147614539	0.079707588
M5005_Spy1064	malC	maltose transport system permease protein	-0.135381425	0.028997701

M5005_Spy1065	amyA	alpha-amylase	-0.150486893	0.020663041
M5005_Spy1066	amyB	neopullulanase/cyclomaltodextrinase/maltogenic alpha-amylase	-0.270234517	3.92313E-06
M5005_Spy1067	malX	maltose/maltodextrin-binding protein	-0.419161272	9.7197E-15
M5005_Spy1068	M5005_Spy1068	transposase	-0.595992535	2.14565E-09
M5005_Spy1069	M5005_Spy1069	esterase	-0.185810681	0.004770442
M5005_Spy1070	dltD	protein precursor	-2.754178196	0.000432419
M5005_Spy1075	uvrB	excinuclease ABC subunit B	-0.614268961	8.46179E-13
M5005_Spy1081	M5005_Spy1081	PTS system, cellobiose-specific IIA component	0.211330964	0.062755564
M5005_Spy1085	bglA2	beta-glucosidase	0.225321267	0.001714623
M5005_Spy1086	M5005_Spy1086	nicotinamide mononucleotide transporter	0.12560052	0.075730336
M5005_Spy1094	M5005_Spy1094	transporter, MFS superfamily	0.26784177	0.003823032
M5005_Spy1097	M5005_Spy1097	phosphorylase, Pnp/Udp family	0.241880353	0.020179413
M5005_Spy1099	M5005_Spy1099	transcriptional regulator, LytR family	-0.371112968	7.64254E-11
M5005_Spy1102	M5005_Spy1102	ribonuclease BN	-0.29608233	0.039666652
M5005_Spy1108	metK2	S-adenosylmethionine synthetase	-1.470981694	0.003090914
M5005_Spy1110	birA	biotin operon repressor/biotin--[acetyl-CoA-carboxylase] synthetase	-0.595948793	0.030466932
M5005_Spy1115	M5005_Spy1115	putative membrane spanning protein	-0.252840367	0.028614514
M5005_Spy1116	udk	uridine kinase	-0.286922827	0.012623158
M5005_Spy1119	gapN	NADP-dependent glyceraldehyde-3-phosphate dehydrogenase	5.807651869	0.020074681
M5005_Spy1120	pstI	phosphoenolpyruvate-protein phosphotransferase	-3.011272175	0.006719284
M5005_Spy1133	surA	peptidyl-prolyl cis-trans isomerase	-0.39763532	0.000388025
M5005_Spy1139	nagB	glucosamine-6-phosphate isomerase	-2.093827111	2.88467E-45
M5005_Spy1147	comEC	COME operon protein 3	0.148873576	0.01593488
M5005_Spy1151	M5005_Spy1151	hypothetical protein with endo/excinuclease domain	-0.261493392	0.00193561
M5005_Spy1152	M5005_Spy1152	kup system potassium uptake protein	0.128619751	0.028614514
M5005_Spy1155	prfC	bacterial peptide chain release factor 3 (RF-3)	0.357879816	0.009073766
M5005_Spy1157	murF	UDP-N-acetylmuramoylalanine-D-glutamyl-lysine-- D-alanyl-D-alanine ligase	6.719714196	0.013461377
M5005_Spy1159	recR	recombination protein	-1.280897205	0.096045264
M5005_Spy1167	M5005_Spy1167	lead, cadmium, zinc and mercury transporting ATPase	0.199057926	0.022427384
M5005_Spy1170	M5005_Spy1170	putative membrane associated protein	-0.340661019	8.2749E-05
M5005_Spy1173	M5005_Spy1173	phage protein	-0.510577256	0.04034404
M5005_Spy1184	M5005_Spy1184	phage protein	-0.297235957	0.065806857
M5005_Spy1188	M5005_Spy1188	phage protein	0.207693425	0.070456533
M5005_Spy1193	M5005_Spy1193	phage protein	-0.139853802	0.020393212
M5005_Spy1198	M5005_Spy1198	phage protein	-0.2460794	0.042029066
M5005_Spy1205	M5005_Spy1205	phage protein	-0.530447481	0.031023338
M5005_Spy1212	xis	excisionase	-0.283754226	0.001323835
M5005_Spy1215	M5005_Spy1215	phage protein	0.240319288	0.081897455
M5005_Spy1220	M5005_Spy1220	phage protein	-0.217194686	0.001032325
M5005_Spy1222	int2	integrase	-0.145201209	0.020774348
M5005_Spy1223	M5005_Spy1223	DNA-binding protein HU	-0.786453801	0.048158486
M5005_Spy1226	M5005_Spy1226	degV family protein	-0.166376836	0.083125274
M5005_Spy1229	argR1	arginine repressor	-0.287412646	0.070161556
M5005_Spy1238	artQ	arginine transport system permease protein	0.335345657	7.87538E-05
M5005_Spy1239	M5005_Spy1239	putative cytosolic protein	-5.991188884	8.52842E-06
M5005_Spy1240	clpE	ATP-dependent clp protease ATP-binding subunit	-0.311334502	2.68547E-05
M5005_Spy1241	mutT	mutator protein (7,8-dihydro-8-oxoguanine-triphosphatase)	0.306965042	1.40526E-06
M5005_Spy1242	M5005_Spy1242	putative cytosolic protein	-3.825808456	3.99866E-18
M5005_Spy1257	glcK	glucokinase/Xylose repressor	0.463168135	0.000616995
M5005_Spy1259	dpr	non-specific DNA-binding protein/iron-binding ferritin-like antioxidant protein	0.54163315	0.026754736
M5005_Spy1261	M5005_Spy1261	radical SAM family enzyme	-0.277455862	0.000143065
M5005_Spy1269	asnA	aspartate--ammonia ligase	-0.324396241	0.00022588
M5005_Spy1271	M5005_Spy1271	Xaa-His dipeptidase	0.355033327	6.96162E-06
M5005_Spy1272	M5005_Spy1272	arginine/ornithine antiporter	0.34567922	2.73948E-10
M5005_Spy1274	M5005_Spy1274	acetyltransferase	0.352489823	0.062755564
M5005_Spy1275	arcA	arginine deiminase	0.432105002	4.41244E-11
M5005_Spy1276	M5005_Spy1276	transcription regulator, crp family	0.242779764	0.001036328
M5005_Spy1277	ahrC2	arginine repressor	0.384121152	0.001365761
M5005_Spy1278	M5005_Spy1278	hypothetical protein	0.405186181	0.000362317
M5005_Spy1279	M5005_Spy1279	putative cytosolic protein	0.303346171	0.000263411
M5005_Spy1280	M5005_Spy1280	two-component sensor kinase	0.119234668	0.086037306
M5005_Spy1287	M5005_Spy1287	hypothetical protein	0.381899297	0.00033272
M5005_Spy1292	valS	valyl-tRNA synthetase	3.391011069	0.074713922
M5005_Spy1299	M5005_Spy1299	hypothetical protein	0.908935502	0.060043898
M5005_Spy1304	lacZ	beta-galactosidase	0.137980874	0.048679478
M5005_Spy1305	M5005_Spy1305	two-component response regulator	0.205269508	0.040222719
M5005_Spy1306	M5005_Spy1306	two-component sensor kinase	0.132984775	0.059241498
M5005_Spy1307	M5005_Spy1307	putative membrane spanning protein	0.368399167	0.046731974
M5005_Spy1308	M5005_Spy1308	sugar-binding protein	0.145471244	0.053891616
M5005_Spy1309	M5005_Spy1309	sugar transport system permease protein	0.354242186	0.000513027
M5005_Spy1314	hyl	hyaluronoglucosaminidase	-0.227328213	0.014696612
M5005_Spy1316	M5005_Spy1316	hypothetical protein	-0.128551022	0.085160895
M5005_Spy1317	M5005_Spy1317	alpha-mannosidase	-0.245505003	0.001387193
M5005_Spy1325	M5005_Spy1325	ribosome-associated factor Y	-0.221347596	0.041573651
M5005_Spy1326	comFC	COMF operon protein 3	-0.372110262	2.10923E-06
M5005_Spy1330	M5005_Spy1330	S1-type RNA-binding domain	-0.74692518	0.075490492
M5005_Spy1335	M5005_Spy1335	serine/threonine protein kinase	-6.585073438	0.006793532
M5005_Spy1337	sunL	16S rRNA m(5)C 967 methyltransferase	0.239694789	0.053003495
M5005_Spy1348	M5005_Spy1348	D-beta-hydroxybutyrate permease	0.14496096	0.047084545
M5005_Spy1349	luxS	autoinducer-2 production protein	0.343853044	0.004009047
M5005_Spy1355	pbp1A	multimodular transpeptidase-transglycosylase	-0.357965209	0.017686211
M5005_Spy1359	M5005_Spy1359	amino acid permease	-0.304658981	0.01702493
M5005_Spy1367	ftsL	cell division protein	-4.551689394	0.000108524

M5005_Spy1368	mraW	S-adenosyl-methyltransferase	1.67930909	0.090073585
M5005_Spy1372	proB	ABC transporter permease protein	0.133261314	0.058546555
M5005_Spy1373	M5005_Spy1373	ABC transporter ATP-binding protein	-0.356746012	3.41443E-06
M5005_Spy1375	tkt	transketolase	-2.51859695	3.09045E-34
M5005_Spy1380	glpO	alpha-glycerophosphate oxidase	0.291621409	0.001966032
M5005_Spy1381	glpK	glycerol kinase	-0.387105112	0.000176667
M5005_Spy1388	nagA	N-acetylglucosamine-6-phosphate deacetylase	-2.967080193	9.81928E-67
M5005_Spy1399	M5005_Spy1399	PTS system, galactose-specific IIC component	0.966600808	9.68348E-46
M5005_Spy1400	M5005_Spy1400	PTS system, galactose-specific IIB component	0.766318977	6.56928E-56
M5005_Spy1401	M5005_Spy1401	PTS system, galactose-specific IIA component	0.748829764	7.90408E-17
M5005_Spy1402	lacR1	lactose phosphotransferase system repressor	-0.553815332	4.66666E-15
M5005_Spy1407	M5005_Spy1407	esterase	0.19769693	0.000922219
M5005_Spy1415	sdaD2	phage-encoded streptodornase	-0.465311301	1.02888E-19
M5005_Spy1418	M5005_Spy1418	phage protein	-0.586820285	1.06578E-08
M5005_Spy1424	M5005_Spy1424	phage endopeptidase	-0.24846314	0.005541929
M5005_Spy1429	M5005_Spy1429	phage protein	0.723358181	0.037713588
M5005_Spy1432	M5005_Spy1432	phage protein	-0.242009298	0.067313007
M5005_Spy1434	M5005_Spy1434	phage protein	-0.134451994	0.064042121
M5005_Spy1439	M5005_Spy1439	portal protein	-1.046023558	2.75952E-94
M5005_Spy1440	M5005_Spy1440	terminase large subunit	-0.26903225	0.01702493
M5005_Spy1444	M5005_Spy1444	adenine-specific methyltransferase	-0.483602884	1.22193E-05
M5005_Spy1445	M5005_Spy1445	phage protein	-3.11670764	0.0019036
M5005_Spy1447	M5005_Spy1447	phage-related DNA helicase	-0.36668461	0.001549324
M5005_Spy1450	M5005_Spy1450	phage-encoded DNA polymerase	-0.392703831	0.001036328
M5005_Spy1452	M5005_Spy1452	phage protein	-1.357985539	0.002521384
M5005_Spy1453	M5005_Spy1453	phage protein	-0.52535912	0.000867075
M5005_Spy1456	M5005_Spy1456	phage protein	-5.865683189	0.007275635
M5005_Spy1459	M5005_Spy1459	phage protein	4.948664617	0.070190509
M5005_Spy1462	M5005_Spy1462	phage protein	-3.37461592	0.002345239
M5005_Spy1467	int3	integrase	0.162128269	0.028614514
M5005_Spy1468	M5005_Spy1468	tRNA (m(7)G46) methyltransferase	-4.531276109	2.1863E-104
M5005_Spy1472	hit	bis(5'-nucleosyl)-tetraphosphatase (asymmetrical)	-0.749819225	3.43726E-29
M5005_Spy1473	M5005_Spy1473	hypothetical protein	-0.416631681	0.093032464
M5005_Spy1479	manL	PTS system, mannose-specific IIAB component	-0.297216685	0.003410052
M5005_Spy1481	manN	PTS system, mannose-specific IID component	-0.23871645	0.003859713
M5005_Spy1485	accA	acetyl-coenzyme A carboxylase carboxyl transferase subunit beta	5.150732112	0.053003495
M5005_Spy1498	dnaK	chaperone protein	-2.761056382	0.073631479
M5005_Spy1505	M5005_Spy1505	hypothetical protein	0.231450181	0.019244545
M5005_Spy1512	codY	transcription pleiotropic repressor	-2.430113697	0.046340261
M5005_Spy1513	M5005_Spy1513	aspartate aminotransferase	-0.291579792	5.6449E-05
M5005_Spy1515	M5005_Spy1515	hydrolase, HAD superfamily	0.151260986	0.035734291
M5005_Spy1516	asnB	L-asparaginase	0.266714114	0.047444226
M5005_Spy1519	recG	ATP-dependent DNA helicase	-1.196217506	7.00861E-22
M5005_Spy1527	M5005_Spy1527	ferrichrome transport system permease protein	0.362243739	0.007434617
M5005_Spy1528	M5005_Spy1528	ferrichrome-binding protein	0.225701691	0.035989381
M5005_Spy1531	isp2	immunogenic secreted protein	0.147449983	0.042690936
M5005_Spy1538	pmi	mannose-6-phosphate isomerase	0.549596975	0.000337974
M5005_Spy1543	scrB	sucrose-6-phosphate hydrolase	0.58307602	0.008441112
M5005_Spy1544	scrR	sucrose operon repressor	0.424628928	1.48977E-09
M5005_Spy1546	M5005_Spy1546	general stress protein, Gls24 family	4.610387445	0.096298826
M5005_Spy1554	ssb3	phage single-strand DNA binding protein	-2.883450906	0.016433127
M5005_Spy1556	M5005_Spy1556	hypothetical protein	-0.295605182	0.078666556
M5005_Spy1558	M5005_Spy1558	transcriptional regulator	-0.183045694	0.055279479
M5005_Spy1560	M5005_Spy1560	phosphatidylglycerophosphatase B	-0.601488049	4.68211E-12
M5005_Spy1561	mutS2	DNA mismatch repair protein	-0.318372284	1.31956E-06
M5005_Spy1566	recD	exodeoxyribonuclease V alpha chain	-0.198012638	0.003992761
M5005_Spy1569	pfl	formate acetyltransferase	0.219644111	0.051658108
M5005_Spy1571	M5005_Spy1571	c3-degrading proteinase	0.158398807	0.012028133
M5005_Spy1575	norA	quinolone resistance protein	0.288128283	0.070372434
M5005_Spy1583	M5005_Spy1583	putative cytosolic protein	-0.689740143	0.06131078
M5005_Spy1585	deoC	deoxyribose-phosphate aldolase	-0.176406289	0.008518949
M5005_Spy1586	nupC	nucleoside permease	0.210194156	0.00127592
M5005_Spy1595	M5005_Spy1595	Zn-dependent hydrolase	4.136269425	0.046803536
M5005_Spy1607	fba	fructose-bisphosphate aldolase	3.187168475	0.068861964
M5005_Spy1608	M5005_Spy1608	alpha/beta hydrolase	-0.305972667	0.025041831
M5005_Spy1611	rpoE	DNA-directed RNA polymerase delta chain	-1.062584944	8.28587E-39
M5005_Spy1612	ropA	trigger factor, ppiase	0.537905124	0.005470417
M5005_Spy1617	M5005_Spy1617	tRNA pseudouridine synthase A	-1.687984388	0.000853296
M5005_Spy1620	M5005_Spy1620	glycerate kinase	0.186255087	0.074439808
M5005_Spy1625	salR	transcriptional regulatory protein	-0.213017119	0.004571132
M5005_Spy1626	M5005_Spy1626	sensory transduction protein kinase	-0.201910317	0.025361034
M5005_Spy1628	M5005_Spy1628	ABC transporter ATP-binding protein	-0.155640457	0.068694134
M5005_Spy1636	lacC2	tagatose-6-phosphate kinase	-0.235748911	0.001214377
M5005_Spy1637	lacB2	galactose-6-phosphate isomerase lacB subunit	-0.184879789	0.0884185
M5005_Spy1638	lacA2	galactose-6-phosphate isomerase lacA subunit	-0.466057228	1.1464E-09
M5005_Spy1639	lacR2	lactose phosphotransferase system repressor	0.442227619	3.26984E-16
M5005_Spy1641	M5005_Spy1641	putative cytosolic protein	-0.193258928	0.030466932
M5005_Spy1653	M5005_Spy1653	putative membrane associated protein	-0.178210109	0.056205149
M5005_Spy1654	M5005_Spy1654	hypothetical protein	0.218946555	0.027782755
M5005_Spy1658	cysE	serine acetyltransferase	0.284746722	0.021035449
M5005_Spy1660	M5005_Spy1660	polyribonucleotide nucleotidyltransferase	1.21659417	0.007300065
M5005_Spy1661	M5005_Spy1661	transaldolase	0.320638041	0.012028133
M5005_Spy1664	M5005_Spy1664	transcription antiterminator, BglG family	0.129232287	0.033876806

M5005_Spy1666	M5005_Spy1666	SSU ribosomal protein S15P	1.26756869	0.044442389
M5005_Spy1678	M5005_Spy1678	thioredoxin	0.532943513	0.042744784
M5005_Spy1683	lrp	leucine rich protein	0.26514934	0.054447704
M5005_Spy1684	ska	streptokinase	-0.167414356	0.00396604
M5005_Spy1692	M5005_Spy1692	PTS system, glucose-specific IIABC component	0.22754629	0.004130506
M5005_Spy1695	M5005_Spy1695	ribosomal protein L11 methyltransferase	0.378942638	0.039960084
M5005_Spy1699	M5005_Spy1699	ATPase, AAA family	0.438937934	3.33963E-07
M5005_Spy1701	flaR	DNA topology modulation protein flar-related protein	0.219567905	0.002521455
M5005_Spy1704	dppA	dipeptide-binding protein	0.169272735	0.016433127
M5005_Spy1706	dppC	dipeptide transport system permease protein	0.311227603	0.000724689
M5005_Spy1709	M5005_Spy1709	hypothetical protein	0.609290595	0.076397832
M5005_Spy1716	M5005_Spy1716	transposase	0.285978288	0.000215362
M5005_Spy1723	isp	immunogenic secreted protein	0.187114534	0.014814538
M5005_Spy1726	M5005_Spy1726	ABC transporter permease protein	0.41960124	1.14975E-10
M5005_Spy1727	M5005_Spy1727	ABC transporter ATP-binding protein	0.456661447	3.48731E-11
M5005_Spy1728	M5005_Spy1728	periplasmic component of efflux system	0.597386975	1.89224E-25
M5005_Spy1733	M5005_Spy1733	hypothetical protein	-0.327011341	0.00396604
M5005_Spy1734	spi	streptopain protease inhibitor	-1.306111591	0.055924712
M5005_Spy1737	rgg	transcriptional regulator	-0.287939227	5.13485E-05
M5005_Spy1741	gldA	glycerol dehydrogenase	0.301455488	0.006304499
M5005_Spy1744	M5005_Spy1744	PTS system, cellobiose-specific IIC component	0.318130964	5.43431E-06
M5005_Spy1747	M5005_Spy1747	sorbitol operon regulator	0.173643909	0.04604258
M5005_Spy1748	M5005_Spy1748	transcriptional regulator, DeoR family	-0.229936107	0.003190623
M5005_Spy1749	M5005_Spy1749	pyruvate formate-lyase activating enzyme	0.184752698	0.081288026
M5005_Spy1753	pbp2A	multimodular transpeptidase-transglycosylase	-1.489990102	1.67134E-39
M5005_Spy1754	M5005_Spy1754	translation initiation inhibitor	0.679504841	1.75313E-20
M5005_Spy1758	M5005_Spy1758	putative dipeptidase B	0.166753885	0.020774348
M5005_Spy1770	hutI	imidazolonepropionase	-0.394015218	1.09926E-06
M5005_Spy1771	hutU	urocanate hydratase	0.265960905	0.000724689
M5005_Spy1772	M5005_Spy1772	glutamate formiminotransferase	0.276809073	4.05381E-06
M5005_Spy1779	M5005_Spy1779	transcriptional regulator, LuxR family	0.261823917	8.61926E-05
M5005_Spy1783	dexS	trehalose-6-phosphate hydrolase	-0.412219696	0.031653124
M5005_Spy1784	M5005_Spy1784	PTS system, trehalose-specific IIBC component	0.199426863	0.012515326
M5005_Spy1787	M5005_Spy1787	glyoxalase family protein	0.184634433	0.004600307
M5005_Spy1788	yaaA	protein yaaA	0.271256921	0.001549324
M5005_Spy1794	M5005_Spy1794	putative membrane spanning protein	-0.249876842	0.011433773
M5005_Spy1795	M5005_Spy1795	putative cytosolic protein	-1.000465879	0.01862386
M5005_Spy1799	recA	RecA	-0.69730192	0.089030926
M5005_Spy1802	ruvA	holliday junction DNA helicase	-2.752251314	0.02362407
M5005_Spy1805	mutS	DNA mismatch repair protein	-0.172856862	0.016835327
M5005_Spy1807	argR2	arginine repressor	0.540279525	0.001549324
M5005_Spy1808	argS	arginyl-tRNA synthetase	-0.434441451	0.011862309
M5005_Spy1810	M5005_Spy1810	putative membrane spanning protein	-0.57868553	5.24865E-20
M5005_Spy1811	M5005_Spy1811	putative membrane spanning protein	-0.37349165	0.053447917
M5005_Spy1813	aspS	aspartyl-tRNA synthetase	-4.834405166	0.059571138
M5005_Spy1815	rpmF	LSU ribosomal protein L32P	-6.916976722	0.001147592
M5005_Spy1817	cadD	cadmium resistance protein	0.199311254	0.023764653
M5005_Spy1818	cadC	cadmium efflux system accessory protein	0.586704766	6.96365E-09
M5005_Spy1820	M5005_Spy1820	ftsK/SpoIIIE family	0.308328803	0.002005886
M5005_Spy1828	M5005_Spy1828	phage infection protein	0.386702347	1.36339E-05
M5005_Spy1834	M5005_Spy1834	hypothetical protein	-4.003417166	0.015006992
M5005_Spy1837	M5005_Spy1837	phosphoesterase, DHH family protein	-4.225291276	6.28895E-17
M5005_Spy1842	sdhA	L-serine dehydratase	0.371627958	4.40495E-09
M5005_Spy1843	M5005_Spy1843	transglycosylase SLT domain family protein	0.177933795	0.060683246
M5005_Spy1849	M5005_Spy1849	zinc protease	0.186953924	0.042739332
M5005_Spy1850	M5005_Spy1850	zinc protease	0.206005312	0.028349113
M5005_Spy1851	hasA	hyaluronan synthase	0.173996812	0.005514679
M5005_Spy1854	M5005_Spy1854	putative cytosolic protein	0.426090392	0.05897268
M5005_Spy1856	M5005_Spy1856	glucose uptake protein-like protein	0.137265613	0.079117191
M5005_Spy1857	guaB	inosine-5'-monophosphate dehydrogenase	0.414805974	0.000169353
M5005_Spy1862	M5005_Spy1862	ABC transporter, permease protein	-0.254089421	0.004770442
M5005_Spy1863	M5005_Spy1863	transposase	0.394988955	0.027103422
M5005_Spy1866	parB	chromosome partitioning protein	-0.179182668	0.03784202

* fitness score is defined as log2 fold changes in sequence read counts between input and output pools. Only genes with statistically significant change (q value < 0.1) are listed.
