# Supplementary material for: Novel Genes Required for the Fitness of Streptococcus pyogenes in Human Saliva
Source: mSphere. 2017 Nov 1;2(6):e00460-17. doi: 10.1128/mSphereDirect.00460-17 (PMC5663985; doi:10.1128/mSphereDirect.00460-17)
Supplement: TABLE S6 [file sph006172393st7.pdf]

**TABLE S6 Fitness score of each gene in the M1 GAS genome after 48 hour saliva incubation.**

| locus_tag | gene_name | function | Fitness score* | q.value |
|---|---|---|---|---|
| M5005_Spy0004 | M5005_Spy0004 | GTP-binding protein | -0.80121639 | 0.026443415 |
| M5005_Spy0009 | M5005_Spy0009 | hypothetical protein | -6.467121039 | 0.00052947 |
| M5005_Spy0013 | ftsH | cell division protein | -0.989293892 | 0.002280046 |
| M5005_Spy0014 | M5005_Spy0014 | amino acid permease | -0.244946209 | 0.005182553 |
| M5005_Spy0017 | sibA | secreted protein | -6.628150267 | 3.95358E-05 |
| M5005_Spy0019 | recO | DNA repair protein | -0.810889535 | 0.060294579 |
| M5005_Spy0022 | M5005_Spy0022 | phosphoribosylaminoimidazole-succinocarboxamide synthase | 0.463506302 | 1.74504E-08 |
| M5005_Spy0023 | M5005_Spy0023 | phosphoribosylformylglycinamidine synthase | 0.193846214 | 0.01281588 |
| M5005_Spy0024 | purF | amidophosphoribosyltransferase | 0.696199804 | 3.81893E-22 |
| M5005_Spy0025 | purM | phosphoribosylformylglycinamidine cyclo-ligase | 0.320996766 | 0.000314478 |
| M5005_Spy0033 | purB | adenylosuccinate lyase | 0.362710759 | 2.66928E-05 |
| M5005_Spy0035 | ruvB | holliday junction DNA helicase | -5.388536847 | 0.005013446 |
| M5005_Spy0038 | M5005_Spy0038 | acyltransferase family | -5.077912819 | 0.034134532 |
| M5005_Spy0040 | adhA | alcohol dehydrogenase | 0.487378041 | 0.000153791 |
| M5005_Spy0041 | M5005_Spy0041 | Na+ driven multidrug efflux pump | 0.266866586 | 0.003751017 |
| M5005_Spy0065 | adk | adenylate kinase | -2.475594045 | 0.041836533 |
| M5005_Spy0075 | M5005_Spy0075 | 4-diphosphocytidyl-2-C-methyl-D-erythritol kinase | 0.277208137 | 0.040827722 |
| M5005_Spy0076 | M5005_Spy0076 | 4-diphosphocytidyl-2-C-methyl-D-erythritol kinase | 0.325246039 | 0.055392029 |
| M5005_Spy0077 | adcR | transcriptional regulator, MarR family | 0.900675715 | 0.000103813 |
| M5005_Spy0078 | adcC | high-affinity zinc uptake system ATP-binding protein | 0.282251128 | 0.000187604 |
| M5005_Spy0079 | adcB | high-affinity zinc uptake system membrane protein | 0.263326621 | 0.00125906 |
| M5005_Spy0084 | rpoC | DNA-directed RNA polymerase beta' chain | 0.457230017 | 0.095575452 |
| M5005_Spy0085 | M5005_Spy0085 | putative DNA binding protein | -1.201021059 | 0.085926054 |
| M5005_Spy0089 | M5005_Spy0089 | comG operon protein 4 | -0.373618704 | 0.016956917 |
| M5005_Spy0092 | M5005_Spy0092 | comG operon protein 6 | 0.210116192 | 0.079110579 |
| M5005_Spy0097 | pepA | glutamyl aminopeptidase | 0.235203908 | 0.015581204 |
| M5005_Spy0098 | M5005_Spy0098 | hypothetical protein | 0.42939843 | 0.068725633 |
| M5005_Spy0101 | M5005_Spy0101 | tRNA binding domain protein | 0.373718916 | 2.65764E-05 |
| M5005_Spy0102 | M5005_Spy0102 | single-strand DNA binding protein | 0.212853957 | 0.073437748 |
| M5005_Spy0103 | M5005_Spy0103 | deoxyadenosine kinase/deoxyguanosine kinase | 0.408374505 | 0.000123443 |
| M5005_Spy0104 | M5005_Spy0104 | tRNA-dihydrouridine synthase | -1.724483441 | 1.81124E-32 |
| M5005_Spy0105 | M5005_Spy0105 | 33 kDa chaperonin | -1.051651544 | 1.8081E-08 |
| M5005_Spy0107 | M5005_Spy0107 | fibronectin-binding protein | 0.280174385 | 0.000326299 |
| M5005_Spy0109 | M5005_Spy0109 | fibronectin-binding protein | 0.19814323 | 0.01281588 |
| M5005_Spy0112 | M5005_Spy0112 | transposase | 0.336676534 | 4.05576E-07 |
| M5005_Spy0113 | M5005_Spy0113 | transposase | 0.293925013 | 0.000829247 |
| M5005_Spy0116 | atoE | short-chain fatty acids transporter | 0.22101831 | 0.002562473 |
| M5005_Spy0119 | M5005_Spy0119 | acetyl-CoA acetyltransferase | 0.370482102 | 4.89397E-06 |
| M5005_Spy0121 | M5005_Spy0121 | acetyl-CoA:acetoacetyl-CoA transferase beta subunit | 0.393715791 | 0.011829084 |
| M5005_Spy0122 | M5005_Spy0122 | putative DNA-binding protein | -1.227186208 | 7.96851E-14 |
| M5005_Spy0124 | sloR | transcriptional regulator | 0.383862604 | 1.61404E-06 |
| M5005_Spy0131 | ntpA | V-type sodium ATP synthase subunit A | 0.162451945 | 0.091708441 |
| M5005_Spy0132 | ntpB | V-type sodium ATP synthase subunit B | 0.254761582 | 0.014345671 |
| M5005_Spy0136 | purA | adenylosuccinate synthetase | 0.438147889 | 0.023036067 |
| M5005_Spy0139 | nga | NAD glycohydrolase | 0.173657187 | 0.025149723 |
| M5005_Spy0142 | M5005_Spy0142 | hypothetical protein | 0.420822992 | 0.010120335 |
| M5005_Spy0143 | M5005_Spy0143 | hypothetical protein | 0.485076154 | 0.073437748 |
| M5005_Spy0144 | M5005_Spy0144 | hypothetical protein | -0.559263662 | 0.000266686 |
| M5005_Spy0147 | leuS | leucyl-tRNA synthetase | -5.800450033 | 0.003807562 |
| M5005_Spy0148 | M5005_Spy0148 | PTS system, 3-keto-L-gulonate specific IIC component | 0.236265258 | 0.02858806 |
| M5005_Spy0150 | M5005_Spy0150 | PTS system, 3-keto-L-gulonate specific IIA component | 0.38901477 | 0.042981232 |
| M5005_Spy0153 | araD | L-ribulose-5-phosphate 4-epimerase | 0.641949316 | 1.27144E-05 |
| M5005_Spy0158 | opuABC | glycine betaine-binding protein/glycine betaine transport system permease protein | 0.258706707 | 0.031763716 |
| M5005_Spy0160 | M5005_Spy0160 | CoA binding protein | 0.598663149 | 0.076120107 |
| M5005_Spy0161 | perR | ferric uptake regulation protein | 0.408142232 | 0.034134532 |
| M5005_Spy0169 | M5005_Spy0169 | malonate permease | 0.471727932 | 1.24953E-11 |
| M5005_Spy0175 | tgt | queuine tRNA-ribosyltransferase | 0.262396654 | 0.023202172 |
| M5005_Spy0176 | M5005_Spy0176 | zinc finger protein | 0.294550917 | 0.011970461 |
| M5005_Spy0177 | M5005_Spy0177 | bioY protein | 0.182170037 | 0.073437748 |
| M5005_Spy0178 | M5005_Spy0178 | metal-dependent hydrolase | 0.411832661 | 0.000131456 |
| M5005_Spy0180 | M5005_Spy0180 | S-layer homology domain | 0.226477301 | 0.002553307 |
| M5005_Spy0182 | speG | exotoxin type G precursor | -0.306100467 | 0.003242166 |
| M5005_Spy0183 | M5005_Spy0183 | putative membrane associated protein | 0.475894873 | 1.63466E-05 |
| M5005_Spy0196 | M5005_Spy0196 | multidrug resistance ABC transporter ATP-binding and permease protein | 1.033497918 | 7.29201E-17 |
| M5005_Spy0197 | M5005_Spy0197 | multidrug resistance ABC transporter ATP-binding and permease protein | 0.722724919 | 6.83084E-10 |
| M5005_Spy0198 | M5005_Spy0198 | putative cytosolic protein | 0.363978533 | 0.027759554 |
| M5005_Spy0199 | M5005_Spy0199 | deoxyuridine 5'-triphosphate nucleotidohydrolase | 1.015124317 | 0.050478912 |
| M5005_Spy0200 | radA | DNA repair protein | 0.293524189 | 0.020591145 |
| M5005_Spy0201 | M5005_Spy0201 | carbonic anhydrase | 0.884250218 | 2.11032E-06 |
| M5005_Spy0205 | fasC | sensory transduction protein kinase | -0.163852014 | 0.073437748 |
| M5005_Spy0208 | M5005_Spy0208 | 60 kDa inner membrane protein YIDC | 0.492884799 | 0.001013129 |
| M5005_Spy0209 | M5005_Spy0209 | jag protein | 0.508362754 | 0.033839732 |
| M5005_Spy0212 | M5005_Spy0212 | N-acetylmannosamine-6-phosphate 2-epimerase | 0.455620545 | 0.009955499 |
| M5005_Spy0213 | M5005_Spy0213 | N-acetylneuraminate-binding protein | 0.397488606 | 4.46295E-05 |
| M5005_Spy0214 | M5005_Spy0214 | N-acetylneuraminate transport system permease protein | 0.322255752 | 9.78105E-05 |
| M5005_Spy0217 | nanH | N-acetylneuraminate lyase | 0.504756214 | 0.000115354 |
| M5005_Spy0218 | M5005_Spy0218 | N-acetylmannosamine kinase | 0.601263302 | 0.001769122 |
| M5005_Spy0224 | rpe | ribulose-phosphate 3-epimerase | -2.083383598 | 6.64893E-05 |
| M5005_Spy0225 | M5005_Spy0225 | thiamin pyrophosphokinase | -2.337037891 | 4.48623E-08 |
| M5005_Spy0229 | prgA | surface exclusion protein | 0.347619408 | 0.005855709 |
| M5005_Spy0240 | M5005_Spy0240 | undecaprenyl-phosphate alpha-N-acetylglucosaminephosphotransferase | -1.515247992 | 1.76367E-08 |
| M5005_Spy0241 | rgpG | putative cytosolic protein | -0.884096547 | 4.26258E-09 |

| | | | | |
|---|---|---|---|---|
| M5005_Spy0246 | M5005_Spy0246 | ABC transporter-associated protein | 0.406650426 | 0.02192047 |
| M5005_Spy0247 | M5005_Spy0247 | D-alanyl-D-alanine carboxypeptidase | 0.778085762 | 5.14257E-26 |
| M5005_Spy0249 | oppA | oligopeptide-binding protein | -0.79729279 | 0.018141862 |
| M5005_Spy0251 | oppC | oligopeptide transport system permease protein | -1.003417326 | 0.001602549 |
| M5005_Spy0253 | oppF | oligopeptide transport ATP-binding protein | -0.701962833 | 0.0177399 |
| M5005_Spy0267 | M5005_Spy0267 | putative cytosolic protein | 0.27203195 | 0.000324183 |
| M5005_Spy0268 | M5005_Spy0268 | hypothetical protein | -0.431157455 | 0.0336946 |
| M5005_Spy0270 | M5005_Spy0270 | ABC transporter substrate-binding protein | -1.283741621 | 0.037893538 |
| M5005_Spy0271 | M5005_Spy0271 | ABC transporter substrate-binding protein | 0.708441769 | 1.69254E-27 |
| M5005_Spy0272 | M5005_Spy0272 | ABC transporter ATP-binding protein | 0.589353106 | 7.40574E-11 |
| M5005_Spy0273 | M5005_Spy0273 | ABC transporter permease protein | 0.610197338 | 3.01508E-16 |
| M5005_Spy0274 | braB | branched-chain amino acid transport system carrier protein | 0.421796002 | 2.67003E-12 |
| M5005_Spy0280 | M5005_Spy0280 | heat shock protein | 0.4537848 | 0.030400319 |
| M5005_Spy0284 | M5005_Spy0284 | putative regulatory protein | 0.716586026 | 0.094935422 |
| M5005_Spy0292 | M5005_Spy0292 | aminodeoxychorismate lyase family | -3.178649659 | 0.001602549 |
| M5005_Spy0293 | greA | transcription elongation factor | -3.671640444 | 0.000647673 |
| M5005_Spy0300 | M5005_Spy0300 | hydrolase, HAD superfamily | 1.219908623 | 4.68589E-08 |
| M5005_Spy0301 | M5005_Spy0301 | integral membrane protein | -0.639476931 | 0.00292796 |
| M5005_Spy0306 | M5005_Spy0306 | CBS domain containing protein | -0.4484368 | 0.099476807 |
| M5005_Spy0308 | M5005_Spy0308 | putative cytosolic protein | -0.454277169 | 0.070987323 |
| M5005_Spy0312 | M5005_Spy0312 | 23S rRNA methyltransferase | 0.458124661 | 0.001962543 |
| M5005_Spy0314 | M5005_Spy0314 | phosphatidylglycerophosphatase B-like protein | -0.470570153 | 9.93959E-06 |
| M5005_Spy0316 | M5005_Spy0316 | SAM-dependent methyltransferase | 0.333100392 | 0.039741746 |
| M5005_Spy0322 | fhuB | ferrichrome transport system permease protein | 0.217962872 | 0.046276251 |
| M5005_Spy0326 | M5005_Spy0326 | export protein for polysaccharides and teichoic acids | -0.789552653 | 0.001909207 |
| M5005_Spy0327 | upp | uracil phosphoribosyltransferase | -3.760793283 | 3.56E-47 |
| M5005_Spy0330 | tmk | thymidylate kinase | -5.474524159 | 0.005126851 |
| M5005_Spy0336 | M5005_Spy0336 | putative membrane associated protein | 0.227719554 | 0.066639105 |
| M5005_Spy0339 | exoA | exodeoxyribonuclease III | -0.521764974 | 0.001201726 |
| M5005_Spy0347 | nrdF1 | ribonucleoside-diphosphate reductase beta chain | 0.414548993 | 0.01183171 |
| M5005_Spy0349 | nrdE1 | ribonucleoside-diphosphate reductase alpha chain | 0.239585762 | 0.00231244 |
| M5005_Spy0355 | M5005_Spy0355 | hypothetical protein | -0.413641052 | 0.033509226 |
| M5005_Spy0356 | speJ | exotoxin type J precursor | -0.531416664 | 8.08557E-06 |
| M5005_Spy0360 | M5005_Spy0360 | NAD-dependent oxidoreductase | 0.560109837 | 0.073777816 |
| M5005_Spy0362 | gcaD | glucosamine-1-phosphate acetyltransferase/UDP-N-acetylglucosamine pyrophosphorylase | -2.819901279 | 0.027283575 |
| M5005_Spy0382 | msrA2 | peptide methionine sulfoxide reductase | -0.450389941 | 0.063082975 |
| M5005_Spy0386 | phoH | phoH protein | 0.251675584 | 0.008742256 |
| M5005_Spy0392 | M5005_Spy0392 | hypothetical protein | -0.275849052 | 0.097700924 |
| M5005_Spy0401 | M5005_Spy0401 | putative cytosolic protein | -0.745819735 | 0.023429239 |
| M5005_Spy0402 | M5005_Spy0402 | hypothetical protein | 0.212113026 | 0.045483892 |
| M5005_Spy0403 | M5005_Spy0403 | hypothetical protein | 0.345460377 | 0.011247255 |
| M5005_Spy0404 | M5005_Spy0404 | hypothetical protein | 0.333799383 | 0.015896016 |
| M5005_Spy0408 | fpg | formamidopyrimidine-DNA glycosylase | 0.77133238 | 0.000104062 |
| M5005_Spy0410 | M5005_Spy0410 | ATPase | 0.371146259 | 0.005101339 |
| M5005_Spy0413 | secG | protein translocase subunit | -5.454139643 | 0.010122892 |
| M5005_Spy0414 | M5005_Spy0414 | exoribonuclease II | 0.658883991 | 1.06752E-10 |
| M5005_Spy0416 | M5005_Spy0416 | glutaminyl-peptide cyclotransferase | 0.146665752 | 0.087758496 |
| M5005_Spy0418 | M5005_Spy0418 | permease | 1.195384928 | 0.012376206 |
| M5005_Spy0421 | gloA | lactoylglutathione lyase | -0.348835937 | 0.055819524 |
| M5005_Spy0422 | M5005_Spy0422 | NAD(P)H-dependent quinone reductase | 0.318253322 | 0.089536422 |
| M5005_Spy0424 | ccpA | catabolite control protein A | 5.44809319 | 0.085908597 |
| M5005_Spy0427 | thrS | threonyl-tRNA synthetase | 0.743032579 | 0.059036931 |
| M5005_Spy0430 | M5005_Spy0430 | ABC transporter permease protein | 0.324537781 | 0.038329821 |
| M5005_Spy0436 | vicK | two-component sensor histidine kinase | -0.409116947 | 0.097553316 |
| M5005_Spy0441 | aroE2 | shikimate 5-dehydrogenase | -0.240832379 | 0.00420541 |
| M5005_Spy0442 | M5005_Spy0442 | putative cytosolic protein | -0.503253364 | 0.000112234 |
| M5005_Spy0447 | M5005_Spy0447 | glycosyltransferase involved in cell wall biogenesis | -0.18654923 | 0.050478912 |
| M5005_Spy0448 | M5005_Spy0448 | hypothetical protein | -0.864731121 | 0.002763543 |
| M5005_Spy0449 | M5005_Spy0449 | UDP-glucose 6-dehydrogenase | -0.251396648 | 0.02086061 |
| M5005_Spy0450 | mefE | macrolide-efflux protein | -0.246618577 | 0.00473631 |
| M5005_Spy0459 | M5005_Spy0459 | portal protein | 0.199630433 | 0.042981232 |
| M5005_Spy0460 | M5005_Spy0460 | hypothetical protein | 0.359757774 | 0.008286162 |
| M5005_Spy0462 | M5005_Spy0462 | hypothetical protein | -0.3496676 | 0.011235629 |
| M5005_Spy0463 | M5005_Spy0463 | putative cytosolic protein | -0.319129954 | 0.016477617 |
| M5005_Spy0465 | M5005_Spy0465 | hypothetical protein | -0.350703968 | 0.055819524 |
| M5005_Spy0466 | M5005_Spy0466 | hypothetical protein | -0.559105426 | 1.95809E-08 |
| M5005_Spy0476 | bglA | 6-phospho-beta-glucosidase | 0.139432888 | 0.074138854 |
| M5005_Spy0480 | M5005_Spy0480 | transcription accessory protein | 0.254495367 | 0.003921628 |
| M5005_Spy0485 | lgt | prolipoprotein diacylglyceryl transferase | -1.514213018 | 2.90969E-13 |
| M5005_Spy0486 | M5005_Spy0486 | hypothetical protein | -1.335010684 | 1.60421E-48 |
| M5005_Spy0491 | M5005_Spy0491 | peptidase family U32 | 0.195959898 | 0.035799135 |
| M5005_Spy0495 | lysS | lysyl-tRNA synthetase | 5.556998795 | 0.075621278 |
| M5005_Spy0497 | M5005_Spy0497 | phosphoglycerate mutase | 0.24300318 | 0.042251224 |
| M5005_Spy0500 | M5005_Spy0500 | N-acetylmuramoyl-L-alanine amidase | 2.009170538 | 0.003074125 |
| M5005_Spy0504 | pepF | oligoendopeptidase F | 0.231640968 | 0.012708056 |
| M5005_Spy0513 | M5005_Spy0513 | putative cytosolic protein | -0.399169698 | 3.83489E-05 |
| M5005_Spy0517 | regR | transcriptional regulator, LacI family | 0.150030807 | 0.09943065 |
| M5005_Spy0518 | M5005_Spy0518 | oligohyaluronate lyase | 0.173131629 | 0.013228998 |
| M5005_Spy0520 | M5005_Spy0520 | PTS system, N-acetylgalactosamine-specific IIC component | 0.204210298 | 0.036622746 |
| M5005_Spy0522 | M5005_Spy0522 | unsaturated glucuronyl hydrolase | 0.150878671 | 0.095827026 |
| M5005_Spy0524 | idnO | gluconate 5-dehydrogenase | -0.248533492 | 0.037022899 |
| M5005_Spy0525 | M5005_Spy0525 | galactose-6-phosphate isomerase LacB subunit | -0.486542277 | 0.004543723 |
| M5005_Spy0536 | dinG | ATP-dependent helicase | 0.860612947 | 1.64063E-51 |
| M5005_Spy0539 | M5005_Spy0539 | ATP-binding protein | 1.216549546 | 0.000218713 |
| M5005_Spy0542 | pepD | dipeptidase | 0.17046382 | 0.066246537 |

| | | | | |
|---|---|---|---|---|
| M5005_Spy0545 | agaS | galactosamine-6-phosphate deaminase (isomerizing) | 0.187969276 | 0.045050329 |
| M5005_Spy0547 | M5005_Spy0547 | phosphoesterase, DHH family protein | -3.021810625 | 0.083049626 |
| M5005_Spy0554 | M5005_Spy0554 | septation ring formation regulator | 1.590734331 | 0.009463789 |
| M5005_Spy0560 | M5005_Spy0560 | transcriptional regulator | -0.16372523 | 0.055481599 |
| M5005_Spy0561 | epf | putative extracellular matrix binding protein | -0.130974269 | 0.052587559 |
| M5005_Spy0566 | sagE | streptolysin S putative self-immunity protein | -0.886178576 | 5.0364E-08 |
| M5005_Spy0567 | sagF | streptolysin S biosynthesis protein | -0.646069417 | 1.02794E-09 |
| M5005_Spy0568 | sagG | streptolysin S export ATP-binding protein | -3.591807255 | 1.01347E-10 |
| M5005_Spy0569 | sagH | streptolysin S export transmembrane protein | -9.550753286 | 3.97519E-45 |
| M5005_Spy0570 | sagI | streptolysin S export transmembrane protein | -3.535902059 | 0.000896931 |
| M5005_Spy0582 | atpC | ATP synthase epsilon chain | -3.619434894 | 0.035600914 |
| M5005_Spy0583 | M5005_Spy0583 | putative membrane associated protein | -0.482292413 | 0.008864751 |
| M5005_Spy0585 | epuA | epuA protein | -3.431252691 | 0.010310805 |
| M5005_Spy0588 | pheT | phenylalanyl-tRNA synthetase beta chain | 3.374952552 | 0.048432699 |
| M5005_Spy0591 | M5005_Spy0591 | ABC transporter permease protein | -0.356290488 | 0.000625482 |
| M5005_Spy0601 | M5005_Spy0601 | putative cytosolic protein | 0.450310749 | 0.018874614 |
| M5005_Spy0606 | rgpDc | polysaccharide export ATP-binding protein | -2.056855802 | 0.020685526 |
| M5005_Spy0609 | M5005_Spy0609 | phosphoglycerol transferase | -0.208803679 | 0.006397367 |
| M5005_Spy0611 | M5005_Spy0611 | hypothetical protein | -0.741203306 | 1.43172E-08 |
| M5005_Spy0623 | M5005_Spy0623 | methyltransferase | -0.337974689 | 0.062642017 |
| M5005_Spy0627 | gor | glutathione reductase | -0.140512191 | 0.042358766 |
| M5005_Spy0628 | folC2 | folylpolyglutamate synthase/dihydrofolate synthase | 0.258789027 | 0.061712435 |
| M5005_Spy0630 | nifS1 | cysteine desulfhydrase | -4.486916794 | 4.85637E-16 |
| M5005_Spy0631 | thiI | thiamine biosynthesis protein | -4.590172489 | 1.08352E-65 |
| M5005_Spy0632 | capA | capsule biosynthesis protein | 0.184018221 | 0.051535364 |
| M5005_Spy0641 | pyrB | aspartate carbamoyltransferase | -0.288579385 | 0.015988407 |
| M5005_Spy0642 | carA | carbamoyl-phosphate synthase small chain | -0.631877871 | 9.22589E-08 |
| M5005_Spy0643 | carB | carbamoyl-phosphate synthase large chain | -0.706332579 | 1.17224E-30 |
| M5005_Spy0644 | M5005_Spy0644 | periplasmic component of efflux system | -1.541440308 | 4.1128E-99 |
| M5005_Spy0645 | M5005_Spy0645 | ABC transporter ATP-binding protein | -1.44457496 | 8.63787E-42 |
| M5005_Spy0646 | M5005_Spy0646 | ABC transporter permease protein | -1.584508519 | 1.48656E-95 |
| M5005_Spy0647 | M5005_Spy0647 | glycerophosphoryl diester phosphodiesterase | -0.185580246 | 0.017530482 |
| M5005_Spy0650 | M5005_Spy0650 | hypothetical protein | -1.15478348 | 4.72271E-06 |
| M5005_Spy0651 | M5005_Spy0651 | cell surface protein | -0.17118654 | 0.013345994 |
| M5005_Spy0656 | trmD | tRNA (guanine-N(1)-)-methyltransferase | -1.153304497 | 0.043052855 |
| M5005_Spy0659 | apbA | 2-dehydropantoate 2-reductase | -0.240377318 | 0.062493676 |
| M5005_Spy0662 | fruA | PTS system, fructose-specific IIABC component | 0.491315856 | 0.001298911 |
| M5005_Spy0665 | M5005_Spy0665 | transposase | -0.658091204 | 2.65764E-05 |
| M5005_Spy0667 | M5005_Spy0667 | exotoxin type C precursor | -0.513485415 | 0.008731135 |
| M5005_Spy0668 | mac | IgG-degrading protease | -0.138720984 | 0.086878004 |
| M5005_Spy0678 | M5005_Spy0678 | 5'-nucleotidase | -0.158651615 | 0.022373836 |
| M5005_Spy0681 | M5005_Spy0681 | two-component system histidine kinase | 0.497114507 | 2.31717E-14 |
| M5005_Spy0693 | M5005_Spy0693 | hypothetical protein | -0.682978656 | 7.96851E-14 |
| M5005_Spy0694 | clpL | ATP-dependent protease ATP-binding subunit | -0.203670826 | 0.012695731 |
| M5005_Spy0697 | arsC | arsenate reductase family protein | -1.103526379 | 0.039499256 |
| M5005_Spy0701 | cpsY | transcriptional regulator, LysR family | 0.451992162 | 0.000726224 |
| M5005_Spy0702 | M5005_Spy0702 | hypothetical protein | -0.537928537 | 0.00578271 |
| M5005_Spy0703 | pyrF | orotidine 5'-phosphate decarboxylase | 0.227778564 | 0.032126133 |
| M5005_Spy0715 | M5005_Spy0715 | SSU ribosomal protein S1P | 2.815455688 | 0.029015736 |
| M5005_Spy0717 | M5005_Spy0717 | rhodanese-related sulfurtransferase | -0.193034028 | 0.03428942 |
| M5005_Spy0718 | M5005_Spy0718 | hypothetical protein | -0.66768531 | 0.015455493 |
| M5005_Spy0719 | M5005_Spy0719 | glutathione S-transferase | 0.326122642 | 0.001070897 |
| M5005_Spy0720 | M5005_Spy0720 | putative exfoliative toxin | 0.374509326 | 0.000854963 |
| M5005_Spy0722 | miaA | tRNA delta(2)-isopentenylpyrophosphate transferase | -0.897445585 | 1.79528E-06 |
| M5005_Spy0723 | hflX | GTP-binding protein | 0.282690168 | 0.083792854 |
| M5005_Spy0728 | apt | adenine phosphoribosyltransferase | -1.269625217 | 0.011695203 |
| M5005_Spy0732 | M5005_Spy0732 | NIF3-related protein | 0.47300846 | 0.007023517 |
| M5005_Spy0736 | cpsFQ | dTDP-glucose 4,6-dehydratase | -1.388990268 | 0.050082517 |
| M5005_Spy0739 | M5005_Spy0739 | tetratricopeptide repeat family protein | -0.220365503 | 0.01052467 |
| M5005_Spy0743 | M5005_Spy0743 | ABC transporter substrate-binding protein | 0.19731534 | 0.079479868 |
| M5005_Spy0745 | M5005_Spy0745 | ABC transporter permease protein | 0.428030981 | 0.023036067 |
| M5005_Spy0757 | hylA | hyaluronate lyase precursor | -0.184658465 | 0.018862339 |
| M5005_Spy0759 | M5005_Spy0759 | putative membrane associated protein | -2.577094924 | 0.007148767 |
| M5005_Spy0767 | M5005_Spy0767 | 4-nitrophenylphosphatase | 0.308412199 | 0.004047161 |
| M5005_Spy0769 | M5005_Spy0769 | putative cytosolic protein | -0.274591418 | 3.75238E-05 |
| M5005_Spy0770 | M5005_Spy0770 | putative cytosolic protein | -0.23246321 | 0.015180111 |
| M5005_Spy0772 | M5005_Spy0772 | hypothetical protein | -0.436729708 | 3.75238E-05 |
| M5005_Spy0775 | M5005_Spy0775 | nucleoside diphosphate kinase | -0.552164962 | 0.00646147 |
| M5005_Spy0777 | M5005_Spy0777 | collagen-like surface protein | -0.458186186 | 0.001668183 |
| M5005_Spy0779 | M5005_Spy0779 | putative membrane spanning protein | -0.163138512 | 0.041869067 |
| M5005_Spy0780 | M5005_Spy0780 | PTS system, mannose/fructose family IIA component | 0.454006654 | 0.037326927 |
| M5005_Spy0781 | ptsB | PTS system, mannose/fructose family IIB component | 0.611672318 | 7.63204E-06 |
| M5005_Spy0782 | ptsC | PTS system, mannose/fructose family IIC component | 0.376551959 | 0.000569725 |
| M5005_Spy0783 | ptsD | PTS system, mannose/fructose family IID component | 0.331255596 | 0.019975057 |
| M5005_Spy0790 | gabD | succinate-semialdehyde dehydrogenase [NADP+] | -0.284347021 | 0.02086061 |
| M5005_Spy0791 | uvrC | excinuclease ABC subunit C | -0.445433229 | 2.20381E-05 |
| M5005_Spy0793 | M5005_Spy0793 | Xaa-His dipeptidase | 0.560703965 | 0.000100943 |
| M5005_Spy0802 | M5005_Spy0802 | relaxase | -0.381411232 | 0.06865672 |
| M5005_Spy0803 | srtI | protein involved in lantibiotic production | -0.319651566 | 4.02023E-06 |
| M5005_Spy0804 | srtR | nisin biosynthesis two-component response regulator | -0.509964755 | 9.90401E-11 |
| M5005_Spy0805 | srtK | nisin biosynthesis sensor protein | -0.593047294 | 3.99619E-12 |
| M5005_Spy0806 | srtA | lantibiotic precursor | -0.302245633 | 0.031801553 |
| M5005_Spy0807 | srtT | ABC transporter (ATP binding)-lantibiotic associated | -0.287940337 | 0.001915724 |
| M5005_Spy0808 | srtF | lantibiotic transport ATP-binding protein | -0.217577442 | 0.009049016 |
| M5005_Spy0809 | srtE | lantibiotic transport permease protein | -0.310343937 | 0.00050868 |

| | | | | |
|---|---|---|---|---|
| M5005_Spy0810 | srtG | lantibiotic transport permease protein | -0.732801902 | 1.81742E-10 |
| M5005_Spy0817 | dacA1 | D-alanyl-D-alanine carboxypeptidase | -0.646428424 | 2.21266E-11 |
| M5005_Spy0826 | potA | spermidine/putrescine transport system ATP-binding protein | -1.626790119 | 0.001197982 |
| M5005_Spy0829 | M5005_Spy0829 | spermidine/putrescine-binding protein | -0.961070303 | 0.002665322 |
| M5005_Spy0830 | dpiA | transcriptional regulatory protein | -0.20632639 | 0.024495548 |
| M5005_Spy0834 | M5005_Spy0834 | Zn-dependent alcohol dehydrogenase | -0.212902628 | 0.006286012 |
| M5005_Spy0835 | M5005_Spy0835 | class B acid phosphatase | -0.201184949 | 0.009524881 |
| M5005_Spy0837 | M5005_Spy0837 | chloride channel protein | -0.185223605 | 0.039954176 |
| M5005_Spy0838 | M5005_Spy0838 | lipase/acylhydrolase family protein | -0.55727827 | 4.33802E-06 |
| M5005_Spy0840 | radC | DNA repair protein | -0.227820748 | 0.005101339 |
| M5005_Spy0841 | M5005_Spy0841 | glutamine amidotransferase, class I | -0.707558395 | 0.000125584 |
| M5005_Spy0852 | M5005_Spy0852 | short chain dehydrogenase | -0.256792483 | 0.090248662 |
| M5005_Spy0858 | xpt | xanthine phosphoribosyltransferase | -0.268954242 | 0.018862339 |
| M5005_Spy0872 | nox | NADH oxidase H2O-forming | -0.59864277 | 7.62002E-09 |
| M5005_Spy0873 | ldh | L-lactate dehydrogenase | -0.477887123 | 0.019613879 |
| M5005_Spy0874 | gyrA | DNA gyrase subunit A | -0.977681224 | 0.029248331 |
| M5005_Spy0876 | M5005_Spy0876 | lactoylglutathione lyase | -0.25003312 | 0.038977515 |
| M5005_Spy0877 | M5005_Spy0877 | hypothetical protein | -0.473604404 | 0.031736637 |
| M5005_Spy0878 | M5005_Spy0878 | hypothetical protein | -0.234939345 | 0.080297955 |
| M5005_Spy0879 | M5005_Spy0879 | hypothetical protein | -0.261789326 | 0.019374036 |
| M5005_Spy0891 | satD | SatD | 0.617325194 | 0.045483892 |
| M5005_Spy0893 | gid | glucose inhibited division protein A | -0.259273813 | 0.048432699 |
| M5005_Spy0895 | M5005_Spy0895 | putative membrane associated protein | -0.389309652 | 0.064991662 |
| M5005_Spy0898 | M5005_Spy0898 | 2-(5''-triphosphoribosyl)-3'-dephosphocoenzyme-A synthase | -0.188588206 | 0.043052855 |
| M5005_Spy0899 | citG | transcriptional regulator, GntR family | -0.337749688 | 1.58332E-05 |
| M5005_Spy0902 | M5005_Spy0902 | biotin carboxyl carrier protein of oxaloacetate decarboxylase | 0.366176653 | 0.012105096 |
| M5005_Spy0903 | oadB | oxaloacetate decarboxylase beta chain | 0.307217067 | 0.011247255 |
| M5005_Spy0907 | citF | citrate lyase alpha chain/citrate CoA-transferase | 0.171550105 | 0.050478912 |
| M5005_Spy0909 | oadA1 | oxaloacetate decarboxylase alpha chain | 0.216740059 | 0.00420541 |
| M5005_Spy0910 | citC | (citrate [pro-3S]-lyase)-ligase | -0.333546645 | 2.57351E-05 |
| M5005_Spy0911 | M5005_Spy0911 | hypothetical protein | -0.277163007 | 0.003043587 |
| M5005_Spy0912 | M5005_Spy0912 | hypothetical protein | -0.856426057 | 0.079110579 |
| M5005_Spy0914 | M5005_Spy0914 | phage transcriptional repressor | -0.346738044 | 9.02856E-06 |
| M5005_Spy0917 | M5005_Spy0917 | transcriptional regulator, GntR family | -3.369711849 | 0.00210549 |
| M5005_Spy0918 | M5005_Spy0918 | hypothetical protein | -0.397583243 | 0.000129472 |
| M5005_Spy0919 | guaA | GMP synthase (glutamine-hydrolyzing) | -2.644873012 | 0.079479868 |
| M5005_Spy0921 | M5005_Spy0921 | ABC transporter ATP-binding protein | -0.177911404 | 0.025445763 |
| M5005_Spy0922 | pdxK | putative membrane spanning protein | -0.204543515 | 0.064346731 |
| M5005_Spy0923 | M5005_Spy0923 | pyridoxine kinase | -0.255226652 | 0.005013446 |
| M5005_Spy0924 | M5005_Spy0924 | transcriptional regulator, GntR family | -0.206589577 | 0.003163037 |
| M5005_Spy0925 | rnhB | anaerobic ribonucleoside-triphosphate reductase | -0.367195648 | 0.015540508 |
| M5005_Spy0926 | M5005_Spy0926 | cardiolipin synthetase | -4.518570737 | 1.87905E-05 |
| M5005_Spy0933 | M5005_Spy0933 | putative NADH-dependent flavin oxidoreductase | -0.269177519 | 0.018819632 |
| M5005_Spy0934 | M5005_Spy0934 | lipoate-protein ligase A | 0.172382542 | 0.091852654 |
| M5005_Spy0937 | M5005_Spy0937 | transporter | -1.497022595 | 0.03428942 |
| M5005_Spy0938 | pgmA | phosphoglucomutase/phosphomannomutase | -0.704754428 | 0.020334567 |
| M5005_Spy0939 | M5005_Spy0939 | nucleoside transport system permease protein | 1.423525894 | 4.01779E-91 |
| M5005_Spy0940 | M5005_Spy0940 | nucleoside transport system permease protein | 1.522236924 | 7.0464E-117 |
| M5005_Spy0941 | M5005_Spy0941 | nucleoside transport ATP-binding protein | 1.466779159 | 9.4611E-102 |
| M5005_Spy0942 | M5005_Spy0942 | nucleoside-binding protein | 1.11332223 | 7.93514E-49 |
| M5005_Spy0943 | cdd | cytidine deaminase | 1.143622623 | 1.06445E-31 |
| M5005_Spy0944 | M5005_Spy0944 | 16S rRNA m(2)G 1207 methyltransferase | -0.620482362 | 7.22081E-11 |
| M5005_Spy0945 | coaA | pantothenate kinase | -5.221473982 | 0.022902762 |
| M5005_Spy0947 | ciaH | sensor protein | -0.474748144 | 3.00238E-06 |
| M5005_Spy0951 | pstB | phosphate transport ATP-binding protein | -2.978192892 | 2.6596E-117 |
| M5005_Spy0952 | pstB2 | phosphate transport ATP-binding protein | -3.039122372 | 8.19558E-91 |
| M5005_Spy0953 | pstA | phosphate transport system permease protein | -3.66352396 | 3.76273E-82 |
| M5005_Spy0954 | pstC | phosphate transport system permease protein | -3.731565246 | 2.41396E-27 |
| M5005_Spy0955 | pstS | phosphate-binding protein | -3.418296477 | 1.20833E-65 |
| M5005_Spy0961 | truB | tRNA pseudouridine synthase B | -2.988818399 | 0.008611858 |
| M5005_Spy0962 | M5005_Spy0962 | hypothetical protein | -0.334517118 | 0.009641165 |
| M5005_Spy0970 | M5005_Spy0970 | NAD-dependent K+ or Na+ uptake system component | -2.489910656 | 0.003229991 |
| M5005_Spy0971 | M5005_Spy0971 | general stress protein, Gls24 family | -1.314912395 | 7.44134E-07 |
| M5005_Spy0972 | M5005_Spy0972 | hypothetical protein | -1.147445632 | 0.025445763 |
| M5005_Spy0973 | M5005_Spy0973 | general stress protein, Gls24 family | -0.928622544 | 1.00356E-06 |
| M5005_Spy0975 | M5005_Spy0975 | hypothetical protein | -1.850186941 | 2.2057E-06 |
| M5005_Spy0977 | pcrA | DNA helicase II | -6.171575411 | 0.000202935 |
| M5005_Spy0980 | M5005_Spy0980 | cobalt-zinc-cadmium resistance protein | -0.484768716 | 5.37384E-06 |
| M5005_Spy0981 | cfa | cAMP factor | -0.490437364 | 6.58601E-08 |
| M5005_Spy0982 | M5005_Spy0982 | histidine-binding protein | -0.197637301 | 0.052582081 |
| M5005_Spy0987 | sipC | signal peptidase I | -1.84149263 | 1.3023E-51 |
| M5005_Spy0992 | M5005_Spy0992 | ABC transporter ATP-binding protein | -1.509443047 | 6.36708E-09 |
| M5005_Spy0993 | M5005_Spy0993 | ABC transporter permease protein | -1.477594628 | 7.95215E-08 |
| M5005_Spy0995 | M5005_Spy0995 | phage protein | -0.377219036 | 0.050065503 |
| M5005_Spy0998 | M5005_Spy0998 | phage protein | -0.262429119 | 0.074605928 |
| M5005_Spy1000 | M5005_Spy1000 | phage protein | -0.274441447 | 0.001201726 |
| M5005_Spy1001 | M5005_Spy1001 | phage-associated cell wall hydrolase | 0.424829892 | 0.000451039 |
| M5005_Spy1002 | M5005_Spy1002 | N-acetylmuramoyl-L-alanine amidase | -0.305979042 | 0.026600297 |
| M5005_Spy1021 | M5005_Spy1021 | phage protein | -0.624056329 | 2.90969E-13 |
| M5005_Spy1022 | M5005_Spy1022 | portal protein | -0.473504708 | 9.63722E-09 |
| M5005_Spy1023 | M5005_Spy1023 | terminase large subunit | -0.635879032 | 2.22189E-16 |
| M5005_Spy1044 | M5005_Spy1044 | phage protein | -0.319188699 | 0.085908597 |
| M5005_Spy1050 | M5005_Spy1050 | phage transcriptional repressor | -1.354754083 | 3.5842E-05 |
| M5005_Spy1051 | M5005_Spy1051 | phage protein | -0.280017355 | 0.001109135 |
| M5005_Spy1055 | glgP | glycogen phosphorylase | -0.163806986 | 0.022373836 |

| | | | | |
|---|---|---|---|---|
| M5005_Spy1056 | malM | 4-alpha-glucanotransferase | 0.936798644 | 4.06672E-54 |
| M5005_Spy1057 | malR | transcriptional regulator, LacI family | 0.278212091 | 0.000168493 |
| M5005_Spy1058 | malE | maltose/maltodextrin-binding protein | 0.313247685 | 6.64893E-05 |
| M5005_Spy1059 | malF | maltose transport system permease protein | 0.367270557 | 1.46348E-07 |
| M5005_Spy1060 | malG | maltose transport system permease protein | 0.258151957 | 0.004640916 |
| M5005_Spy1061 | M5005_Spy1061 | transcriptional regulator, LacI family | 0.618085185 | 1.3616E-21 |
| M5005_Spy1065 | amyA | alpha-amylase | -0.274386065 | 2.62039E-05 |
| M5005_Spy1066 | amyB | neopullulanase/cyclomaltodextrinase/maltogenic alpha-amylase | -0.579233429 | 4.25792E-15 |
| M5005_Spy1067 | malX | maltose/maltodextrin-binding protein | -0.923463181 | 2.79824E-34 |
| M5005_Spy1068 | M5005_Spy1068 | transposase | -0.915354586 | 1.02556E-10 |
| M5005_Spy1069 | M5005_Spy1069 | esterase | -0.236876084 | 0.018862339 |
| M5005_Spy1075 | uvrB | excinuclease ABC subunit B | -0.578960835 | 6.39446E-09 |
| M5005_Spy1076 | glnH | transporter | 4.977975137 | 0.034134532 |
| M5005_Spy1078 | M5005_Spy1078 | hypothetical protein | -2.257774081 | 0.039513967 |
| M5005_Spy1081 | M5005_Spy1081 | PTS system, cellobiose-specific IIA component | 0.359129573 | 0.022636552 |
| M5005_Spy1083 | M5005_Spy1083 | transcription antiterminator, BglG family | 0.161968481 | 0.015915663 |
| M5005_Spy1084 | M5005_Spy1084 | outer surface protein | 0.21339749 | 0.013905271 |
| M5005_Spy1085 | bglA2 | beta-glucosidase | 0.480840127 | 2.64073E-08 |
| M5005_Spy1086 | M5005_Spy1086 | nicotinamide mononucleotide transporter | 0.56485107 | 8.0465E-15 |
| M5005_Spy1087 | M5005_Spy1087 | putative cytosolic protein | 0.884612135 | 0.068385305 |
| M5005_Spy1094 | M5005_Spy1094 | transporter, MFS superfamily | 0.259696899 | 0.008742256 |
| M5005_Spy1095 | M5005_Spy1095 | putative cytosolic protein | -0.20114759 | 0.088303192 |
| M5005_Spy1097 | M5005_Spy1097 | phosphorylase, Pnp/Udp family | 0.235713751 | 0.050203386 |
| M5005_Spy1099 | M5005_Spy1099 | transcriptional regulator, LytR family | -0.584428981 | 1.33375E-18 |
| M5005_Spy1102 | M5005_Spy1102 | ribonuclease BN | -0.447898576 | 0.03208786 |
| M5005_Spy1108 | metK2 | S-adenosylmethionine synthetase | -2.665666795 | 0.000187604 |
| M5005_Spy1110 | birA | biotin operon repressor/biotin--[acetyl-CoA-carboxylase] synthetase | -1.40299824 | 0.003061224 |
| M5005_Spy1115 | M5005_Spy1115 | putative membrane spanning protein | -0.386317245 | 0.001229649 |
| M5005_Spy1119 | gapN | NADP-dependent glyceraldehyde-3-phosphate dehydrogenase | 6.441865579 | 0.025186074 |
| M5005_Spy1129 | M5005_Spy1129 | CAAX amino terminal protease family | -0.220385518 | 0.01058867 |
| M5005_Spy1130 | M5005_Spy1130 | putative membrane spanning protein | -0.183858675 | 0.05984632 |
| M5005_Spy1133 | surA | peptidyl-prolyl cis-trans isomerase | -0.551867045 | 0.000192463 |
| M5005_Spy1137 | M5005_Spy1137 | putative competence protein/transcription factor | -0.267886336 | 0.071907398 |
| M5005_Spy1139 | nagB | glucosamine-6-phosphate isomerase | -4.193665393 | 1.07975E-14 |
| M5005_Spy1147 | comEC | COME operon protein 3 | 0.217555709 | 0.001629878 |
| M5005_Spy1151 | M5005_Spy1151 | hypothetical protein with endo/excinuclease domain | -0.354245713 | 0.000873811 |
| M5005_Spy1157 | murF | UDP-N-acetylmuramoylalanine-D-glutamyl-lysine-- D-alanyl-D-alanine ligase | 5.556998795 | 0.075621278 |
| M5005_Spy1165 | pyrD | dihydroorotate dehydrogenase | -0.281950298 | 0.006286012 |
| M5005_Spy1167 | M5005_Spy1167 | lead, cadmium, zinc and mercury transporting ATPase | 0.276071356 | 0.011358486 |
| M5005_Spy1170 | M5005_Spy1170 | putative membrane associated protein | -0.494774633 | 2.98226E-06 |
| M5005_Spy1171 | M5005_Spy1171 | phage-associated cell wall hydrolase | 0.210612451 | 0.024969828 |
| M5005_Spy1174 | M5005_Spy1174 | phage protein | 0.385565848 | 0.020320816 |
| M5005_Spy1182 | M5005_Spy1182 | phage protein | -0.731757122 | 0.008286162 |
| M5005_Spy1188 | M5005_Spy1188 | phage protein | 0.290216255 | 0.045335182 |
| M5005_Spy1197 | M5005_Spy1197 | phage protein | -0.456008396 | 0.003538408 |
| M5005_Spy1203 | M5005_Spy1203 | phage protein | 0.389134188 | 0.001668183 |
| M5005_Spy1215 | M5005_Spy1215 | phage protein | 0.495964939 | 0.002686892 |
| M5005_Spy1220 | M5005_Spy1220 | phage protein | -0.22678558 | 0.005190239 |
| M5005_Spy1222 | int2 | integrase | -0.148565939 | 0.041659261 |
| M5005_Spy1223 | M5005_Spy1223 | DNA-binding protein HU | -2.174913445 | 0.048130396 |
| M5005_Spy1226 | M5005_Spy1226 | degV family protein | -0.630511253 | 1.32692E-07 |
| M5005_Spy1235 | M5005_Spy1235 | phosphoglucomutase | 0.225470105 | 0.023982661 |
| M5005_Spy1236 | phr | deoxyribodipyrimidine photolyase | -0.16361355 | 0.07534827 |
| M5005_Spy1237 | artP | arginine transport ATP-binding protein | -0.190283184 | 0.065660389 |
| M5005_Spy1238 | artQ | arginine transport system permease protein | 0.259577814 | 0.027982524 |
| M5005_Spy1240 | clpE | ATP-dependent clp protease ATP-binding subunit | -0.323440964 | 0.000274659 |
| M5005_Spy1242 | M5005_Spy1242 | putative cytosolic protein | -7.608444821 | 1.51381E-33 |
| M5005_Spy1255 | typA | GTP-binding protein | -4.575433519 | 0.000830429 |
| M5005_Spy1269 | asnA | aspartate--ammonia ligase | -0.251033505 | 0.020141515 |
| M5005_Spy1270 | arcC | carbamate kinase | -0.482305522 | 0.039741746 |
| M5005_Spy1271 | M5005_Spy1271 | Xaa-His dipeptidase | 0.502225237 | 4.92346E-07 |
| M5005_Spy1272 | M5005_Spy1272 | arginine/ornithine antiporter | 0.161242981 | 0.054643931 |
| M5005_Spy1273 | arcB | ornithine carbamoyltransferase | -1.319030567 | 1.8134E-11 |
| M5005_Spy1279 | M5005_Spy1279 | putative cytosolic protein | 0.429928638 | 7.50195E-07 |
| M5005_Spy1280 | M5005_Spy1280 | two-component sensor kinase | 0.224361638 | 0.008122161 |
| M5005_Spy1287 | M5005_Spy1287 | hypothetical protein | 0.472912603 | 0.000537535 |
| M5005_Spy1293 | M5005_Spy1293 | hypothetical protein | -0.334049905 | 0.049929831 |
| M5005_Spy1297 | M5005_Spy1297 | phospho-2-dehydro-3-deoxyheptonate aldolase | -0.540603823 | 0.084059288 |
| M5005_Spy1301 | M5005_Spy1301 | putative cytosolic protein | 0.378482422 | 0.000125584 |
| M5005_Spy1304 | lacZ | beta-galactosidase | 0.408229233 | 5.58481E-08 |
| M5005_Spy1305 | M5005_Spy1305 | two-component response regulator | 0.4667239 | 0.000112969 |
| M5005_Spy1306 | M5005_Spy1306 | two-component sensor kinase | 0.488308793 | 2.92151E-09 |
| M5005_Spy1307 | M5005_Spy1307 | putative membrane spanning protein | 0.840468479 | 3.92899E-08 |
| M5005_Spy1308 | M5005_Spy1308 | sugar-binding protein | 0.361881858 | 1.17645E-05 |
| M5005_Spy1309 | M5005_Spy1309 | sugar transport system permease protein | 0.546001949 | 0.000169225 |
| M5005_Spy1310 | M5005_Spy1310 | sugar transport system permease protein | 0.553540433 | 5.52214E-07 |
| M5005_Spy1317 | M5005_Spy1317 | alpha-mannosidase | -0.301693882 | 9.69997E-05 |
| M5005_Spy1318 | rocA | sensory transduction protein kinase | 1.398347665 | 0.084124674 |
| M5005_Spy1319 | M5005_Spy1319 | tRNA (uracil-5-)-methyltransferase | -0.132432433 | 0.099630691 |
| M5005_Spy1320 | recX | regulatory protein | -0.194478815 | 0.022641037 |
| M5005_Spy1325 | M5005_Spy1325 | ribosome-associated factor Y | -1.099584576 | 4.04882E-23 |
| M5005_Spy1326 | comFC | COMF operon protein 3 | -0.405790057 | 4.83685E-06 |
| M5005_Spy1328 | M5005_Spy1328 | Xaa-Pro dipeptidase | -0.231903753 | 0.070341295 |
| M5005_Spy1329 | cysM | cysteine synthase | 0.288006618 | 0.000647673 |
| M5005_Spy1333 | yvqE | two-component sensor protein | 0.366821548 | 0.013230485 |

| | | | | |
|---|---|---|---|---|
| M5005_Spy1344 | atoB | acetyl-CoA acetyltransferase | -0.444354546 | 0.015527665 |
| M5005_Spy1348 | M5005_Spy1348 | D-beta-hydroxybutyrate permease | 0.218834135 | 0.015540508 |
| M5005_Spy1349 | luxS | autoinducer-2 production protein | 0.29427768 | 0.018862339 |
| M5005_Spy1355 | pbp1A | multimodular transpeptidase-transglycosylase | -0.561682942 | 0.013925526 |
| M5005_Spy1356 | pepC | aminopeptidase C | 0.169469759 | 0.064346731 |
| M5005_Spy1359 | M5005_Spy1359 | amino acid permease | -0.561029009 | 0.002763543 |
| M5005_Spy1366 | M5005_Spy1366 | division specific D,D-transpeptidase/cell division protein ftsI | 3.371976055 | 0.097918619 |
| M5005_Spy1372 | proB | ABC transporter permease protein | 0.233415478 | 0.002429291 |
| M5005_Spy1373 | M5005_Spy1373 | ABC transporter ATP-binding protein | -0.202126916 | 0.093583862 |
| M5005_Spy1375 | tkt | transketolase | -3.962639533 | 6.29298E-36 |
| M5005_Spy1377 | M5005_Spy1377 | trans-acting positive regulator | -0.477973153 | 0.000888004 |
| M5005_Spy1380 | glpO | alpha-glycerophosphate oxidase | 0.240273589 | 0.035634592 |
| M5005_Spy1381 | glpK | glycerol kinase | -0.433757478 | 0.002733999 |
| M5005_Spy1382 | M5005_Spy1382 | putative membrane associated protein | -0.344693195 | 0.080866813 |
| M5005_Spy1388 | nagA | N-acetylglucosamine-6-phosphate deacetylase | -7.212465764 | 5.2677E-157 |
| M5005_Spy1389 | M5005_Spy1389 | sodium-dependent phosphate transporter | -0.148806118 | 0.066687474 |
| M5005_Spy1391 | M5005_Spy1391 | degV family protein | 1.736095736 | 0.052582081 |
| M5005_Spy1399 | M5005_Spy1399 | PTS system, galactose-specific IIC component | 0.784935252 | 7.88432E-18 |
| M5005_Spy1400 | M5005_Spy1400 | PTS system, galactose-specific IIB component | 0.490815896 | 1.04592E-13 |
| M5005_Spy1401 | M5005_Spy1401 | PTS system, galactose-specific IIA component | 0.383376922 | 0.003497479 |
| M5005_Spy1402 | lacR1 | lactose phosphotransferase system repressor | -1.366480206 | 7.25676E-31 |
| M5005_Spy1407 | M5005_Spy1407 | esterase | 0.226700319 | 0.001668183 |
| M5005_Spy1415 | sdaD2 | phage-encoded streptodornase | -0.253198709 | 0.009657764 |
| M5005_Spy1418 | M5005_Spy1418 | phage protein | -0.497771868 | 0.000246481 |
| M5005_Spy1419 | M5005_Spy1419 | phage protein | 0.424467921 | 0.018862339 |
| M5005_Spy1420 | M5005_Spy1420 | phage protein | -0.846504521 | 0.005417691 |
| M5005_Spy1423 | M5005_Spy1423 | hyaluronoglucosaminidase | 0.275804483 | 0.001902523 |
| M5005_Spy1431 | M5005_Spy1431 | phage protein | 0.67520532 | 0.018862339 |
| M5005_Spy1438 | M5005_Spy1438 | phage protein | -0.281395691 | 0.000229437 |
| M5005_Spy1439 | M5005_Spy1439 | portal protein | -1.439983846 | 8.4564E-127 |
| M5005_Spy1440 | M5005_Spy1440 | terminase large subunit | -0.412153157 | 0.006194723 |
| M5005_Spy1444 | M5005_Spy1444 | adenine-specific methyltransferase | -0.311112327 | 0.039954176 |
| M5005_Spy1445 | M5005_Spy1445 | phage protein | -4.664231618 | 0.001085592 |
| M5005_Spy1449 | M5005_Spy1449 | DNA primase | 0.217251909 | 0.085116271 |
| M5005_Spy1456 | M5005_Spy1456 | phage protein | -4.990186237 | 0.042220946 |
| M5005_Spy1462 | M5005_Spy1462 | phage protein | -5.267644344 | 1.66913E-05 |
| M5005_Spy1465 | M5005_Spy1465 | phage protein | -0.424735962 | 0.001915724 |
| M5005_Spy1467 | int3 | integrase | 0.180163013 | 0.06865672 |
| M5005_Spy1468 | M5005_Spy1468 | tRNA (m(7)G46) methyltransferase | -13.00104577 | 3.2463E-165 |
| M5005_Spy1472 | hit | bis(5'-nucleosyl)-tetraphosphatase (asymmetrical) | -1.211067462 | 1.12156E-35 |
| M5005_Spy1473 | M5005_Spy1473 | hypothetical protein | -0.974893093 | 1.96627E-05 |
| M5005_Spy1479 | manL | PTS system, mannose-specific IIAB component | -0.702215056 | 1.36384E-08 |
| M5005_Spy1480 | manM | PTS system, mannose-specific IIC component | -0.937929851 | 2.97938E-06 |
| M5005_Spy1481 | manN | PTS system, mannose-specific IID component | -0.83889263 | 1.93122E-17 |
| M5005_Spy1498 | dnaK | chaperone protein | -5.236670412 | 0.022641037 |
| M5005_Spy1500 | hrcA | heat-inducible transcription repressor | -3.599239831 | 0.005039853 |
| M5005_Spy1504 | M5005_Spy1504 | putative membrane spanning protein | 0.366307101 | 0.02192047 |
| M5005_Spy1509 | M5005_Spy1509 | pyruvate, phosphate dikinase | 0.817043773 | 0.027317056 |
| M5005_Spy1512 | codY | transcription pleiotropic repressor | -2.959789517 | 0.039513967 |
| M5005_Spy1513 | M5005_Spy1513 | aspartate aminotransferase | -0.368993456 | 0.000189285 |
| M5005_Spy1515 | M5005_Spy1515 | hydrolase, HAD superfamily | 0.172917017 | 0.060336408 |
| M5005_Spy1516 | asnB | L-asparaginase | 0.455509504 | 0.000307381 |
| M5005_Spy1519 | recG | ATP-dependent DNA helicase | -1.11657301 | 6.08348E-11 |
| M5005_Spy1527 | M5005_Spy1527 | ferrichrome transport system permease protein | 0.618036991 | 5.33413E-05 |
| M5005_Spy1538 | pmi | mannose-6-phosphate isomerase | 0.628777823 | 0.00138895 |
| M5005_Spy1539 | scrK | fructokinase | 0.414684224 | 0.010664926 |
| M5005_Spy1550 | uvrA | excinuclease ABC subunit A | -0.236775662 | 0.06826675 |
| M5005_Spy1558 | M5005_Spy1558 | transcriptional regulator | -0.301444678 | 0.001966332 |
| M5005_Spy1560 | M5005_Spy1560 | phosphatidylglycerophosphatase B | -1.2080037 | 5.63962E-24 |
| M5005_Spy1561 | mutS2 | DNA mismatch repair protein | -0.584722062 | 3.65797E-09 |
| M5005_Spy1567 | M5005_Spy1567 | hypothetical protein | -0.286061264 | 0.090397294 |
| M5005_Spy1568 | dinP | DNA polymerase IV | 0.202610587 | 0.030632061 |
| M5005_Spy1573 | glpF2 | aquaporin | 1.611622487 | 0.050478912 |
| M5005_Spy1577 | pepXP | Xaa-Pro dipeptidyl-peptidase | 0.340952 | 0.004979884 |
| M5005_Spy1582 | dnaQ | DNA polymerase III, epsilon chain | 0.307848822 | 0.009955499 |
| M5005_Spy1584 | M5005_Spy1584 | NAD(FAD)-utilizing dehydrogenase | 0.441960562 | 0.000317167 |
| M5005_Spy1585 | deoC | deoxyribose-phosphate aldolase | -0.233869603 | 0.02175804 |
| M5005_Spy1586 | nupC | nucleoside permease | 0.223529451 | 0.009561789 |
| M5005_Spy1587 | udp | uridine phosphorylase | 0.270827675 | 0.012740525 |
| M5005_Spy1595 | M5005_Spy1595 | Zn-dependent hydrolase | 5.022470538 | 0.064532535 |
| M5005_Spy1602 | M5005_Spy1602 | putative kinase related to hydroxyacetone kinase | -2.352861447 | 0.080939509 |
| M5005_Spy1608 | M5005_Spy1608 | alpha/beta hydrolase | -0.580446044 | 0.003265802 |
| M5005_Spy1610 | pyrG | CTP synthase | -0.142100121 | 0.052582081 |
| M5005_Spy1611 | rpoE | DNA-directed RNA polymerase delta chain | -1.41963475 | 2.98726E-16 |
| M5005_Spy1612 | ropA | trigger factor, ppiase | 0.70095093 | 0.002163563 |
| M5005_Spy1616 | thiD | phosphomethylpyrimidine kinase/hydroxymethylpyrimidine kinase | -2.492894758 | 0.095126277 |
| M5005_Spy1617 | M5005_Spy1617 | tRNA pseudouridine synthase A | -2.283340962 | 0.0177399 |
| M5005_Spy1625 | salR | transcriptional regulatory protein | -0.20136515 | 0.02883937 |
| M5005_Spy1626 | M5005_Spy1626 | sensory transduction protein kinase | -0.194677778 | 0.054116435 |
| M5005_Spy1632 | lacG | 6-phospho-beta-galactosidase | 0.258338563 | 0.000218513 |
| M5005_Spy1636 | lacC2 | tagatose-6-phosphate kinase | -0.404226621 | 0.000283646 |
| M5005_Spy1638 | lacA2 | galactose-6-phosphate isomerase lacA subunit | -0.328668168 | 0.012289529 |
| M5005_Spy1639 | lacR2 | lactose phosphotransferase system repressor | 0.494478436 | 1.25242E-14 |
| M5005_Spy1640 | M5005_Spy1640 | DNA-damage-inducible protein J | 0.283527932 | 0.015794251 |
| M5005_Spy1649 | M5005_Spy1649 | putative membrane spanning protein | 0.355707135 | 0.00206804 |

| | | | | |
|---|---|---|---|---|
| M5005_Spy1651 | M5005_Spy1651 | putative cytosolic protein | 0.259398798 | 0.081912993 |
| M5005_Spy1659 | M5005_Spy1659 | hypothetical protein | 0.453523068 | 0.073437748 |
| M5005_Spy1660 | M5005_Spy1660 | polyribonucleotide nucleotidyltransferase | 1.245338181 | 0.020348967 |
| M5005_Spy1664 | M5005_Spy1664 | transcription antiterminator, BglG family | 0.362199458 | 5.73456E-06 |
| M5005_Spy1669 | def | peptide deformylase | 3.26014419 | 0.051298786 |
| M5005_Spy1680 | pulA | pullulanase | 0.224386848 | 0.013551182 |
| M5005_Spy1682 | msmK | multiple sugar transport ATP-binding protein | 0.797553942 | 2.07391E-22 |
| M5005_Spy1683 | lrp | leucine rich protein | 0.737409202 | 1.98177E-09 |
| M5005_Spy1685 | M5005_Spy1685 | D-tyrosyl-tRNA(Tyr) deacylase | 0.53485458 | 0.005929472 |
| M5005_Spy1692 | M5005_Spy1692 | PTS system, glucose-specific IIABC component | 0.597116861 | 2.77651E-07 |
| M5005_Spy1695 | M5005_Spy1695 | ribosomal protein L11 methyltransferase | 0.559880437 | 0.022636552 |
| M5005_Spy1697 | M5005_Spy1697 | para-aminobenzoate synthetase component I/4-amino-4-deoxychorismate lyase | 0.239293096 | 0.012376206 |
| M5005_Spy1699 | M5005_Spy1699 | ATPase, AAA family | 0.683634362 | 4.99451E-13 |
| M5005_Spy1700 | M5005_Spy1700 | acetyltransferase | 0.278100883 | 0.070666156 |
| M5005_Spy1701 | flaR | DNA topology modulation protein flar-related protein | 0.309190756 | 0.000449019 |
| M5005_Spy1702 | smeZ | mitogenic exotoxin Z | -0.256644386 | 0.01183171 |
| M5005_Spy1704 | dppA | dipeptide-binding protein | 0.177291251 | 0.084144004 |
| M5005_Spy1705 | dppB | dipeptide transport system permease protein | 0.295792923 | 0.001602549 |
| M5005_Spy1706 | dppC | dipeptide transport system permease protein | 0.416414853 | 0.000248447 |
| M5005_Spy1707 | dppD | dipeptide transport ATP-binding protein | 0.326891016 | 0.000693831 |
| M5005_Spy1708 | dppE | dipeptide transport ATP-binding protein | 0.264083142 | 0.020320816 |
| M5005_Spy1710 | M5005_Spy1710 | streptococcal histidine triad protein | 0.185034073 | 0.029788019 |
| M5005_Spy1716 | M5005_Spy1716 | transposase | 0.393492466 | 3.31866E-05 |
| M5005_Spy1719 | emm10 | M protein | -0.607432361 | 1.24953E-11 |
| M5005_Spy1720 | mga | trans-acting positive regulator | -0.416490412 | 1.91766E-11 |
| M5005_Spy1723 | isp | immunogenic secreted protein | 0.2862176 | 0.002852784 |
| M5005_Spy1726 | M5005_Spy1726 | ABC transporter permease protein | 0.696430374 | 1.04083E-20 |
| M5005_Spy1727 | M5005_Spy1727 | ABC transporter ATP-binding protein | 0.594670157 | 7.12678E-14 |
| M5005_Spy1728 | M5005_Spy1728 | periplasmic component of efflux system | 0.830880895 | 4.61118E-26 |
| M5005_Spy1730 | M5005_Spy1730 | hypothetical protein | 0.331111849 | 0.001354501 |
| M5005_Spy1733 | M5005_Spy1733 | hypothetical protein | -0.571358682 | 0.001602549 |
| M5005_Spy1735 | speB | streptococcal pyrogenic exotoxin B | -0.448614901 | 1.46348E-07 |
| M5005_Spy1737 | rgg | transcriptional regulator | -0.60803916 | 1.01158E-12 |
| M5005_Spy1741 | gldA | glycerol dehydrogenase | 0.477242048 | 0.001350488 |
| M5005_Spy1744 | M5005_Spy1744 | PTS system, cellobiose-specific IIC component | 0.332225662 | 3.53777E-05 |
| M5005_Spy1749 | M5005_Spy1749 | pyruvate formate-lyase activating enzyme | 0.273188698 | 0.036267549 |
| M5005_Spy1753 | pbp2A | multimodular transpeptidase-transglycosylase | -1.701073932 | 1.21918E-38 |
| M5005_Spy1754 | M5005_Spy1754 | translation initiation inhibitor | 0.578133079 | 8.59764E-09 |
| M5005_Spy1758 | M5005_Spy1758 | putative dipeptidase B | 0.224893912 | 0.012922985 |
| M5005_Spy1763 | clpC | negative regulator of genetic competence | 0.527358057 | 0.002822606 |
| M5005_Spy1764 | ctsR | transcriptional regulator | -2.112263538 | 0.017323405 |
| M5005_Spy1769 | ahpF | peroxiredoxin reductase (NAD(P)H) | -1.608013058 | 4.78705E-08 |
| M5005_Spy1770 | hutI | imidazolonepropionase | -0.658878789 | 7.49962E-08 |
| M5005_Spy1771 | hutU | urocanate hydratase | 0.223761645 | 0.025186074 |
| M5005_Spy1772 | M5005_Spy1772 | glutamate formiminotransferase | 0.535045256 | 1.59808E-11 |
| M5005_Spy1773 | M5005_Spy1773 | formiminotetrahydrofolate cyclodeaminase | 0.452499942 | 0.000502414 |
| M5005_Spy1774 | fhs2 | formate--tetrahydrofolate ligase | 0.293682962 | 0.092294308 |
| M5005_Spy1776 | M5005_Spy1776 | amino acid permease | 0.287974874 | 0.054082194 |
| M5005_Spy1778 | hutG | formiminoglutamase | 0.311724949 | 0.056344786 |
| M5005_Spy1779 | M5005_Spy1779 | transcriptional regulator, LuxR family | 0.36470297 | 8.17407E-06 |
| M5005_Spy1781 | M5005_Spy1781 | protein translation elongation factor Ts | -5.108954746 | 0.012376206 |
| M5005_Spy1782 | pepO | neutral endopeptidase | -0.229625995 | 0.05278458 |
| M5005_Spy1783 | dexS | trehalose-6-phosphate hydrolase | -1.191324288 | 1.63034E-05 |
| M5005_Spy1787 | M5005_Spy1787 | glyoxalase family protein | 0.160235378 | 0.079360043 |
| M5005_Spy1788 | yaaA | protein yaaA | 0.255043352 | 0.01895794 |
| M5005_Spy1790 | M5005_Spy1790 | acetyltransferase | 0.449027849 | 0.047012372 |
| M5005_Spy1795 | M5005_Spy1795 | putative cytosolic protein | -1.386113921 | 0.015769961 |
| M5005_Spy1797 | M5005_Spy1797 | putative cytosolic protein | 0.729447051 | 0.079431343 |
| M5005_Spy1807 | argR2 | arginine repressor | 0.732316047 | 5.52603E-05 |
| M5005_Spy1808 | argS | arginyl-tRNA synthetase | -0.673288887 | 0.001422107 |
| M5005_Spy1809 | uviB | bacteriocin | -0.291216386 | 0.016747596 |
| M5005_Spy1810 | M5005_Spy1810 | putative membrane spanning protein | -1.971145625 | 5.58352E-60 |
| M5005_Spy1811 | M5005_Spy1811 | putative membrane spanning protein | -0.472299452 | 0.062886601 |
| M5005_Spy1817 | cadD | cadmium resistance protein | 0.249493028 | 0.041999538 |
| M5005_Spy1820 | M5005_Spy1820 | ftsK/SpoIIIE family | 0.386963026 | 0.001421108 |
| M5005_Spy1823 | M5005_Spy1823 | integral membrane protein | -0.916375635 | 0.019816437 |
| M5005_Spy1828 | M5005_Spy1828 | phage infection protein | 0.268573588 | 0.020320816 |
| M5005_Spy1834 | M5005_Spy1834 | hypothetical protein | -5.17957345 | 0.018862339 |
| M5005_Spy1837 | M5005_Spy1837 | phosphoesterase, DHH family protein | -7.356812768 | 3.8605E-29 |
| M5005_Spy1840 | trmU | tRNA (5-methylaminomethyl-2-thiouridylate)-methyltransferase | -5.815019933 | 0.010094839 |
| M5005_Spy1842 | sdhA | L-serine dehydratase | 0.557134673 | 3.0844E-06 |
| M5005_Spy1843 | M5005_Spy1843 | transglycosylase SLT domain family protein | 0.434316506 | 0.000318608 |
| M5005_Spy1848 | M5005_Spy1848 | putative membrane associated protein | 4.837011797 | 0.065629818 |
| M5005_Spy1851 | hasA | hyaluronan synthase | 0.214319893 | 0.001422107 |
| M5005_Spy1852 | hasB | UDP-glucose 6-dehydrogenase | -0.233693097 | 0.001201726 |
| M5005_Spy1853 | hasC | UTP--glucose-1-phosphate uridylyltransferase | 0.324775405 | 5.05641E-05 |
| M5005_Spy1856 | M5005_Spy1856 | glucose uptake protein-like protein | -0.22040789 | 0.015259435 |
| M5005_Spy1857 | guaB | inosine-5'-monophosphate dehydrogenase | 0.882414252 | 2.46744E-24 |
| M5005_Spy1862 | M5005_Spy1862 | ABC transporter, permease protein | -0.5200987 | 2.46798E-09 |
| M5005_Spy1864 | M5005_Spy1864 | putative cytosolic protein | -0.253255289 | 0.027283575 |
| M5005_Spy1866 | parB | chromosome partitioning protein | -0.305944422 | 0.006097699 |

* fitness score is defined as log2 fold changes in sequence read counts between input and output pools. Only genes with statistically significant change (q value < 0.1) are listed.
