# Supplementary figures and images for: Novel Genes Required for the Fitness of Streptococcus pyogenes in Human Saliva
Source: mSphere. 2017 Nov 1;2(6):e00460-17. doi: 10.1128/mSphereDirect.00460-17 (PMC5663985; doi:10.1128/mSphereDirect.00460-17)

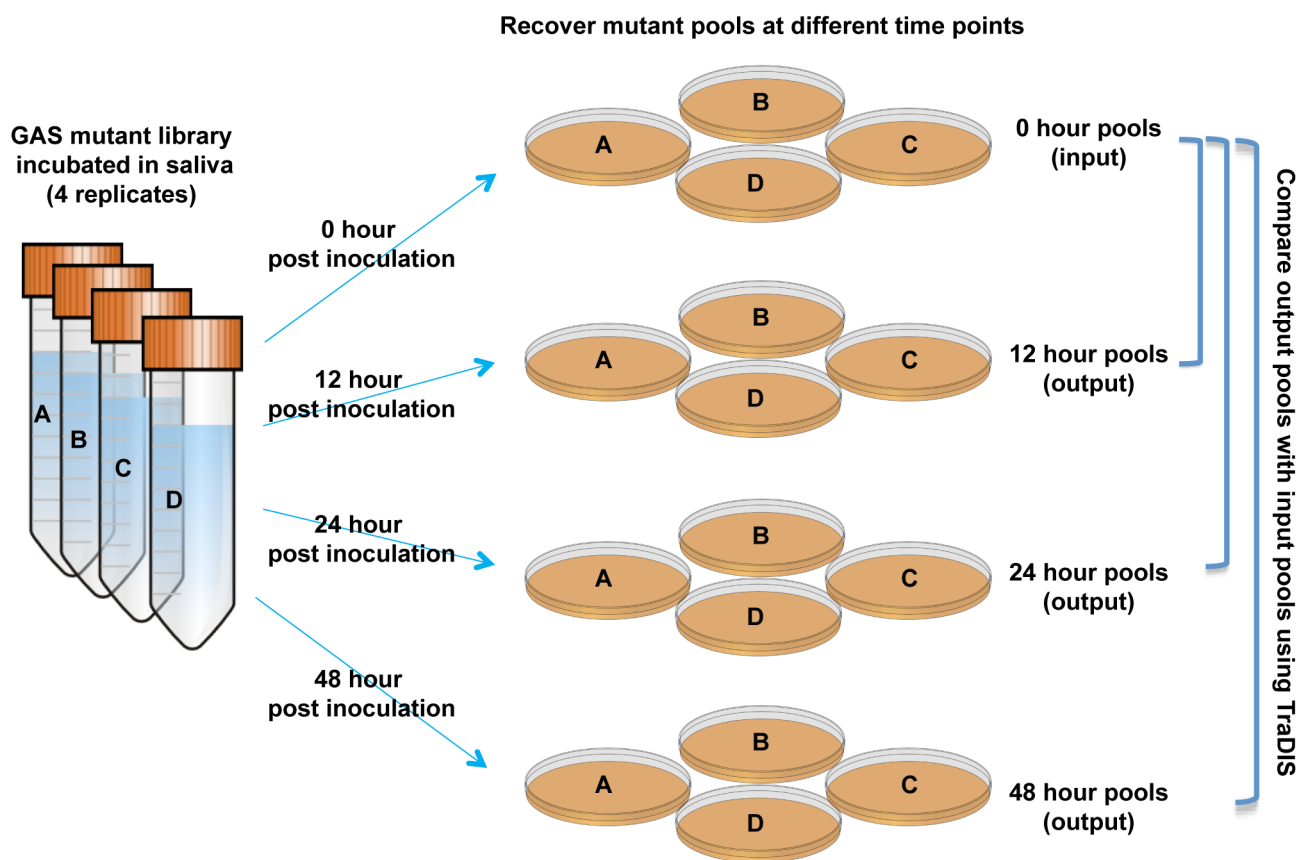

**FIG S1 Experimental strategy for TraDIS mutant screens in human saliva.**

Supplement: FIG S1 [file sph006172393sf1.pdf]
