# Supplementary material for: Novel Genes Required for the Fitness of Streptococcus pyogenes in Human Saliva
Source: mSphere. 2017 Nov 1;2(6):e00460-17. doi: 10.1128/mSphereDirect.00460-17 (PMC5663985; doi:10.1128/mSphereDirect.00460-17)
Supplement: TABLE S7 [file sph006172393st8.pdf]

**TABLE S7 Primers used in this study.**

| Primer name | Sequence | Remarks |
|---|---|---|
| Spy0644-1 | GCGTGGATCCCAGTCAGCTTATGATACTGCTGTTA | BamHI site is underlined |
| Spy0644-2 | GCGTGGATCCCTGGTTGAGAGGACTTATAATATCA | BamHI site is underlined |
| lacR.1-1 | GTCCGGATCCCTGATATGACAGTTCGTAGAGACTT | BamHI site is underlined |
| lacR.1-2 | GTCCGGATCCAGCTAGCTTTTGAATCTCTCCTTCA | BamHI site is underlined |
| Spy0646-1 | GTCCGGATCCCACGAAGTGAAGCCAGAGTGGTTA | BamHI site is underlined |
| Spy0646-2 | GTCCGGATCCTTCTCTAGTTCGCTCTGTGACAGAT | BamHI site is underlined |
| carB-1 | GTCCGGATCCTTGGTGGAACTGGTGGAGGAATATG | BamHI site is underlined |
| carB-2 | GTCCGGATCCATCAATCTTGGTCAAGCTCTCAAGT | BamHI site is underlined |
| nifS1-1 | GTCCGGATCCGCGTCTCGCATTTTAGAAGCTTCC | BamHI site is underlined |
| nifS1-2 | GTCCGGATCCATCTGGGTAAGCGGACAAAGATTTG | BamHI site is underlined |
| pstS-1 | GTCCGGATCCCTACAACCCTTAGTAGAAGCAGTAG | BamHI site is underlined |
| pstS-2 | GTCCGGATCCATTTGCCTTAAACCCATTTAATTGC | BamHI site is underlined |
| Indexing PCR primer 1 | CAAGCAGAAGACGGCATACGAGATCGGTTCGCCTTAACACTCTTTCCCTACACGACGCTCTTCCGATCT | Index sequence is underlined |
| Indexing PCR primer 2 | CAAGCAGAAGACGGCATACGAGATCGGTCTAGTACGACACTCTTTCCCTACACGACGCTCTTCCGATCT | Index sequence is underlined |
| Indexing PCR primer 4 | CAAGCAGAAGACGGCATACGAGATCGGTGCTCAGGAACACTCTTTCCCTACACGACGCTCTTCCGATCT | Index sequence is underlined |
| Indexing PCR primer 3 | CAAGCAGAAGACGGCATACGAGATCGGTTTCTGCCTACACTCTTTCCCTACACGACGCTCTTCCGATCT | Index sequence is underlined |
| Indexing PCR primer 5 | CAAGCAGAAGACGGCATACGAGATCGGTAGGAGTCCACACTCTTTCCCTACACGACGCTCTTCCGATCT | Index sequence is underlined |
| Indexing PCR primer 6 | CAAGCAGAAGACGGCATACGAGATCGGTCATGCCTAACACTCTTTCCCTACACGACGCTCTTCCGATCT | Index sequence is underlined |
| Indexing PCR primer 7 | CAAGCAGAAGACGGCATACGAGATCGGTGTAGAGAGACACTCTTTCCCTACACGACGCTCTTCCGATCT | Index sequence is underlined |
| Indexing PCR primer 8 | CAAGCAGAAGACGGCATACGAGATCGGTCCTCTCTGACACTCTTTCCCTACACGACGCTCTTCCGATCT | Index sequence is underlined |
| Adaptor primer 1 | P-GATCGGAAGAGCACACGTCT | P = Phosphorylation |
| Adaptor primer 2 | ACACTCTTTCCCTACACGACGCTCTTCCGATC*T | * = Phosphorothioate bond |
| Specific ISS1 primer | AATGATACGGCGACCACCGAGATCTACACGTTCATTGATATATCCTCGCTG | |
| Custom read 1 sequencing primer | GTTCATTGATATATCCTCGCTGTCATTTTTATTCATTTTACACTAAAATAGACTTAT | |
| Custom index read sequencing primer | AGATCGGAAGAGCGTCGTGTAGGGAAAGAGTGT | |

Note: Adaptor primer 1 and Adaptor primer 2 (Oligonucleotide sequences © 2007 - 2012 Illumina, Inc. all rights reserved)
